# Disruptions of neurological services, its causes and mitigation strategies during COVID-19: a global review

**DOI:** 10.1007/s00415-021-10588-5

**Published:** 2021-05-22

**Authors:** David García-Azorín, Katrin M. Seeher, Charles R. Newton, Njideka U. Okubadejo, Andrea Pilotto, Deanna Saylor, Andrea Sylvia Winkler, Chahnez Charfi Triki, Matilde Leonardi

**Affiliations:** 1 Headache Unit, Department of Neurology. Hospital, Clínico Universitario de Valladolid, Avenida Ramón y Cajal 3, 47005 Valladolid, Spain; 2 Department of Mental Health and Substance Use, World Health Organization, Geneve, Switzerland; 3 Department of Psychiatry, University of Oxford, Oxford, UK; 4 Neurology Unit, Department of Medicine, College of Medicine, University of Lagos, Lagos, Nigeria; 5 Department of Clinical and Experimental Sciences, Neurology Unit, University of Brescia, Brescia, Italy; 6 Department of Neurology, Johns Hopkins University School of Medicine, Baltimore, MD USA; 7 Centre for Global Health, Department of Neurology, Technical University of Munich, Munich, Germany; 8 Centre for Global Health, Institute of Health and Society, University of Oslo, Oslo, Norway; 9 Child neurology department-Hedi Chaker Hospital, LR19ES 15-Sfax University, Sfax, Tunisia; 10 Neurology, Public Health, Disability Unit, Fondazione IRCCS Istituto Neurologico Carlo Besta, Milan, Italy

**Keywords:** Nervous system diseases, Neurology, Health services administration, Telemedicine, COVID-19

## Abstract

**Background:**

The COVID-19 pandemic leads to disruptions of health services worldwide. To evaluate the particular impact on neurological services a rapid review was conducted.

**Methods:**

Studies reporting the provision of neurological services during the pandemic and/or adopted mitigation strategies were included in this review. PubMed and World Health Organization’s (WHO) COVID-19 database were searched. Data extraction followed categories used by WHO COVID-19 pulse surveys and operational guidelines on maintaining essential health services during COVID-19.

**Findings:**

The search yielded 1101 articles, of which 369 fulfilled eligibility criteria, describing data from 210,419 participants, being adults (81%), children (11.4%) or both (7.3%). Included articles reported data from 105 countries and territories covering all WHO regions and World Bank income levels (low income: 1.9%, lower middle: 24.7%, upper middle: 29.5% and high income; 44.8%). Cross-sectoral services for neurological disorders were most frequently disrupted (62.9%), followed by emergency/acute care (47.1%). The degree of disruption was at least moderate for 75% of studies. Travel restrictions due to lockdowns (81.7%) and regulatory closure of services (65.4%) were the most commonly reported causes of disruption. Authors most frequently described telemedicine (82.1%) and novel dispensing approaches for medicines (51.8%) as mitigation strategies. Evidence for the effectiveness of these measures is largely missing.

**Interpretation:**

The COVID-19 pandemic affects all aspects of neurological care. Given the worldwide prevalence of neurological disorders and the potential long-term neurological consequences of COVID-19, service disruptions are devastating. Different strategies such as telemedicine might mitigate the negative effects of the pandemic, but their efficacy and acceptability remain to be seen.

**Supplementary Information:**

The online version contains supplementary material available at 10.1007/s00415-021-10588-5.

## Background

The Coronavirus disease 2019 (COVID-19) pandemic has caused a substantial number of deaths worldwide, surpassing three million casualties as of April 2021 [1]. However, this number does not even begin to quantify the hidden toll of the pandemic—the collateral damage it has caused. Among these are the excess deaths associated with COVID-19 [2, 3] which are at least partly due to disruptions in the healthcare systems, including the discontinuation of emergency and acute care, difficulty accessing routine outpatient services, and difficulties related to accessing essential medications and other therapies such as childhood vaccination programmes contributing to increased mortality and disability [4]. Chronic diseases requiring regular healthcare are particularly affected by the discontinuation and/or reduced capacities of health services and the impact on noncommunicable diseases (NCDs) [5], of which neurological disorders represent the largest part [6–8], still remains to be seen.

The World Health Organization (WHO), as part of its *COVID-19 strategic preparedness and response plan* released operational guidance on maintaining essential services during COVID-19 [9], containing recommendations for mental, neurological and substance use (MNS) disorders focusing on maintaining emergency/acute care, treatment and care in outpatient settings, residential care, cross-sectoral service delivery such as for example community services or inclusive schooling and mental and brain health promotion. Patient associations and scientific societies have also published guidelines and conducted surveys in the first months of the pandemic [10, 11]. A rapid assessment of MNS services conducted by WHO with 130 Ministries of Health worldwide during June–August 2020 highlighted the disruption of essential MNS services in most countries [4]. While the rapid assessment did not specifically focus on neurological services (in fact, often information on neurological services was not readily available to Ministries of Health despite the fact that neurological disorders, including dementia and stroke, represent the leading cause of disability-adjusted life years [6, 15]), it nevertheless demonstrated that surgery for neurological patients was disrupted in 1 out of 3 countries, emergency care for neurological patients was at least slightly disrupted, and outpatient neurological care was severely disrupted in most countries. The WHO authors concluded that valid data and better evidence, especially regarding the use of routine and innovative forms of information and communications technology, such as telehealth or mobile phone apps [12] were needed to mitigate the effects of the pandemic on service disruptions [4].

Telemedicine, defined as the use of information and communication technologies to improve patient outcomes by increasing access to care and medical information, was coined in the 1970’s [13]. However, before the pandemic, the service provision rate of telemedicine was just over 33% in a survey conducted by the Global Observatory for eHealth in 114 countries in 2009 [14]. In the context of COVID-19, WHO defines telemedicine as “solutions (including) clinical consultations conducted via video, chat or text message, staffed helplines, e-pharmacies and mobile clinics with remote connections to health facilities for timely access to patient data such as medication lists and diagnostic test results” [9].

To what extent these and other mitigation strategies are used, and their effectiveness monitored during the pandemic remains unknown. To this end, we conducted a rapid review of the published evidence regarding the impact of the COVID-19 pandemic on disruptions of neurological services and the mitigation strategies implemented for the care of patients with neurological disorders.

## Materials and methods

To evaluate the impact of the COVID-19 pandemic on disruptions of neurological services and the implemented mitigation strategies, WHO commissioned this rapid review on the topic. The literature search was conducted according to the Preferred Reporting Items for Systematic Reviews and Meta-analyses (PRISMA) [16]. A standardized data extraction sheet was designed in line with the categories used in WHO’s COVID-19 Pulse Surveys as well as the Rapid Assessment of MNS disorders [4]. The study was done in parallel with a Global Survey on disruptions and mitigation strategies coordinated by the European Federation of Neurological Associations (EFNA) in collaboration with 34 scientific and patient associations related to neurology in support of WHO’s Neurology and COVID-19 Global Forum working group on Essential Neurological Services. We used the same variables, to allow future comparability between the results of the survey and the present study.

### Selection criteria

Studies were included if they addressed the impact of the COVID-19 pandemic on the provision of neurological services, adopted or proposed mitigation strategies, or both. Studies were excluded in the following cases: (i) publication before November 2019; (ii) lacking original data; (iii) publication in a language other than English, Spanish, French, Italian, Portuguese or German; (iv) focus on basic science or preclinical aspects of the infection; and (v) focus on clinical aspects, diagnosis or therapeutics only.

### Search strategy

Two databases were screened, PubMed and the WHO COVID-19 database, a curated database of all COVID-19 related published articles and pre-publications. The search was conducted on February 18, 2021 and updated on February 28, 2021. The search string was developed together with a WHO librarian combining terms on three axes: (1) COVID-19 related terms, (2) neurological categories and (3) outcomes related to service disruption and mitigation strategies [17]. The full detail on the search is available in the supplementary appendix.

### Study selection criteria

A single author (D GA) screened all search results to identify studies meeting inclusion criteria. The studies were ordered chronologically and included in a spreadsheet. Both the title and the abstract of the studies were reviewed. Whenever eligibility could not be determined by the title and abstract alone, the full articles were screened for eligibility. When the study did not fulfil eligibility criteria, the reason for exclusion was described in the database.

### Data extraction process and extracted information

The method of data extraction was automatic from the PubMed database for the following variables: title, authors, citation, journal, digital object identifier (DOI) and date of creation in PubMed; the remaining variables were manually extracted. For the WHO database, all data were manually extracted using a standardized form. The extracted information included the publication date, the studied population (adult, children or both), the subspecialty of neurology, language of publication, country, where the study took place, study design, and study setting (inpatient, emergency care, outpatient, or a combination). The full list of subspecialties and study designs is available in the supplementary appendix.

The sample size was also described, and in those studies that accounted for patients from 2020 and historical controls, we only included patients studied in 2020. When the study analyzed specialties of medicine other than neurology, only the neurological patients were included in the sample size. If the study described the opinion of healthcare providers, caregivers or students, the number of participants interviewed was included as the sample size.

### Specific variables evaluated for service disruption and mitigation strategies

Data extraction followed the same categories of services, causes for disruption, and mitigation strategies as used by WHO’s COVID-19 Pulse Surveys, the Rapid Assessment of MNS services as well as WHO’s operational guidelines on maintaining essential health services during COVID-19 (chapter on MNS disorders) [4, 9], with additional delineations as and when necessary.

First, we extracted whether the study described any degree of interruption of the following categories: (1) emergency and acute care for neurological disorders; (2) investigations (including neuroimaging, neurophysiology, lab diagnostics, and others); (3) treatment and care for neurological disorders (including interventions and therapies, such as planned surgeries and access to medicines); (4) neurorehabilitation, inclusive of physiotherapy, speech therapy, occupational therapy, cognitive rehabilitation, and psychology/counselling; (5) cross-sectoral service delivery for neurological disorders, including community-based services, residential long-term care, adult/child day care, special/inclusive school educational programmes for children, interventions for caregivers, and services/programmes delivered by non-governmental organizations; (6) promotion of brain health and prevention of neurological disorders, in addition to implementation activities of national prevention plan and neurology advocacy; (7) training of residents, PhD students or other educational activities; (8) research.

The causes of service disruptions were assessed and classified into: (1) closure of inpatient or outpatient services or consultations as per health authority directive; (2) decrease in outpatient volume due to patients not presenting for care; (3) decreased volume of patients due to cancellation of elective care; (4) inpatient services/hospital beds not available due to saturation; (5) insufficient staff to provide services (e.g., due to quarantine/self-isolation of health-care providers due to COVID-19); (6) clinical staff shifted to provide COVID-19 clinical management or emergency support; (7) insufficient Personal Protective Equipment (PPE) available for health care professionals to provide services; (8) disruption of supply chains resulting in unavailability or stock out of essential medicines, medical diagnostics or other health products at health facilities; (9) travel restrictions hindering access to the health facilities for patients.

The degree of service disruption was graded into no disruption, mild, moderate, or severe, based on the study findings as per the authors judgment. In case the level of disruption was not explicitly reported, the degree was approximated based on the change respective to the baseline period or with other similar studies, as mild (1–39%), moderate (40–69%) or severe (70% or higher).

Mitigation strategies were classified into the following categories: (1) triaging of neurological patients to identify priorities; (2) redirection of patients to alternate care sites (e.g., primary care), reorientation of referral pathways or integration of several services into a single visit; (3) telemedicine deployment to replace in-person consults or other teleconsultation formats; (4) self-care interventions, provision of home-based care, or helplines for patients and caregivers; (5) catch-up campaigns for missed appointments; (6) task-shifting or role delegation; (7) recruitment of additional staff, novel supply chain management and logistics approaches; (8) novel dispensing approaches for medicines, novel prescribing approaches (e.g., tele-prescription, extended drug prescriptions); (9) community communications (e.g., informing on changes to service delivery, addressing misinformation and community fears) to ensure that all citizens are aware and informed of continuity of services and that routine care can always be sought; and (10) government removal of user fees.

### Risk of bias, summary measures and synthesis of results

Since this review was not focused on the results of a therapeutic or diagnostic intervention, whenever any information regarding service disruption or mitigation strategies was present, the study was included in the review. The results were summarized as numbers and percentage of studies per category, over the total of included studies. Traditional tools for the evaluation of bias were not appropriate for the purpose of the study, and, therefore, were not used. Although not necessarily considered a bias in itself, we analyzed whether studies were published in international journals versus national or regional journals for the most frequently studies countries.

### Additional analyses

We classified the represented countries according to the Gross National Income (GNI) per capita, according to the 2019 World Bank atlas [17] criteria, into low income, lower middle, upper middle and high income. The full criteria are available in the supplementary appendix.

## Results

**Fig. 1:**
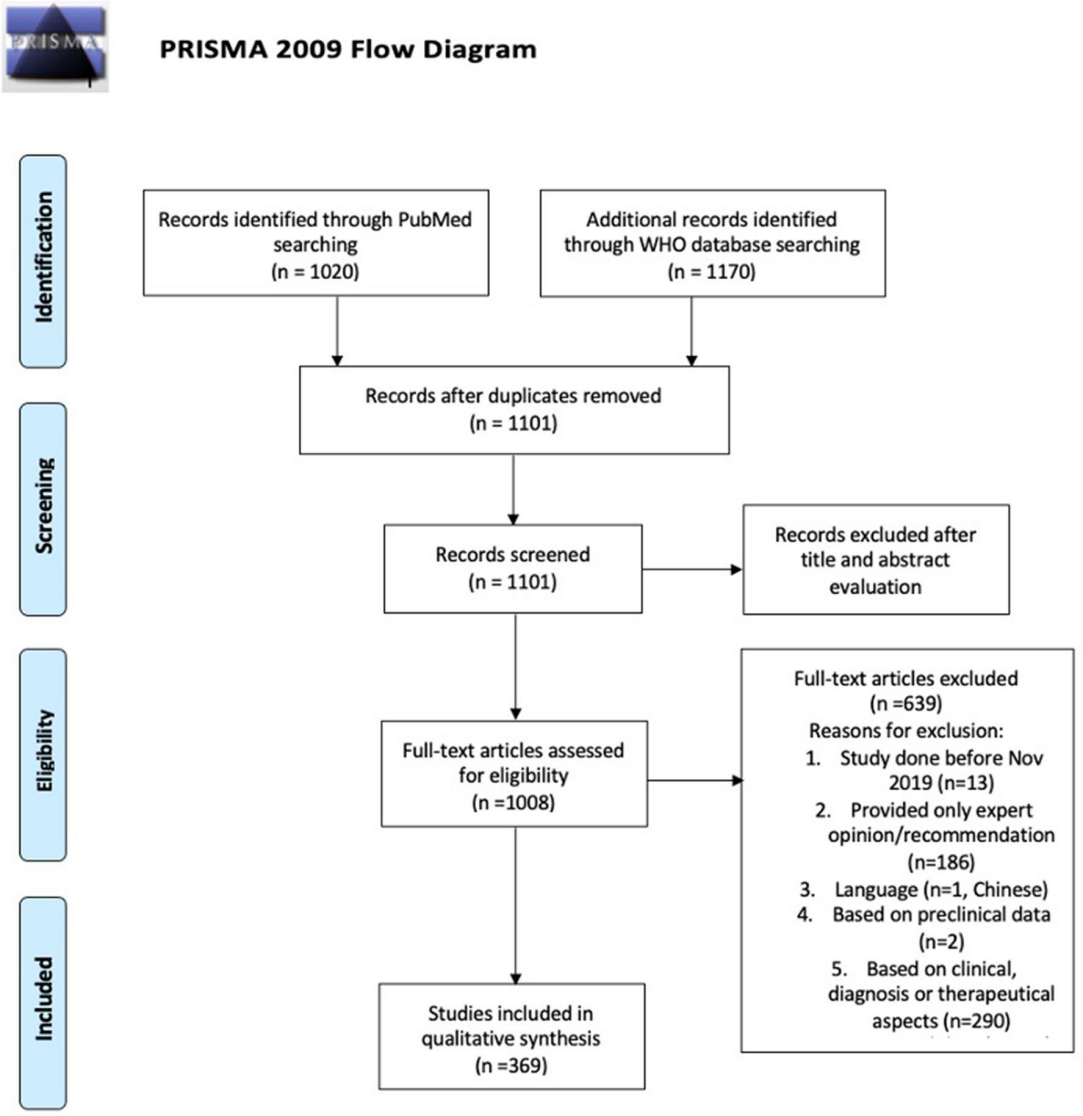
PRISMA flow diagram of screened, included and excluded studies

The search yielded 1,020 matches in PubMed and 1,170 matches in the WHO COVID-19 database. Figure 1 describes the flow diagram of the study selection, including the number of studies identified, screened, included and excluded. Ultimately, 369 articles fulfilled eligibility criteria and provided valid data. [Fig. 1 near here].

The included studies described data from 210,419 participants, with a median number of 127 (IQR: 48–324) participants. The studied population was adults in 295 (81.0%) studies, children in 42 (11.4%) studies, both adults and children in 27 (7.3%) studies and unclear in five (1.4%) studies.

### Descriptive analysis of included studies

#### Publication date

The manuscripts were published in January 2020 in one (0.3%) case, April in 12 (3.2%), May in 47 (12.7%), June in 32 (8.7%), July in 33 (8.9%), August in 48 (13.0%), September in 49 (13.3%), October in 44 (11.9%), November in 40 (10.8%), December in 34 (9.2%), January 2021 in 22 (5.9%) and February 2021 in seven (1.9%) cases.

#### Represented countries

**Fig. 2:**
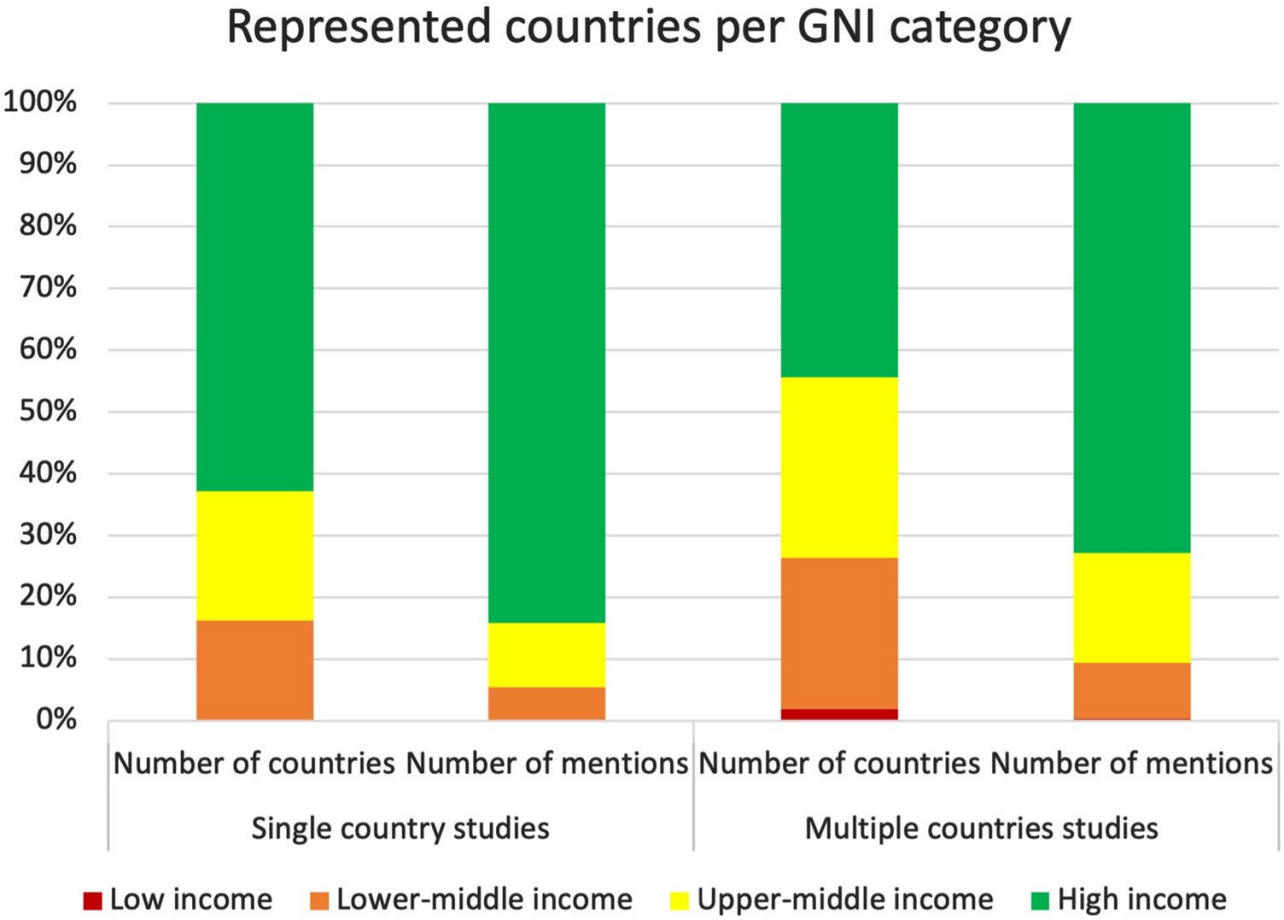
Countries represented in the including studies according to the Gross National Income category

Thirty-five studies represented data from multiple countries (9.5%), while the remaining 334 studies described the situation in a total of 42 different countries (Fig. 2). The most frequently studied country was the United States of America (USA) (*n* = 94 studies, 25.5%), followed by Italy (*n* = 53 studies, 14.4%), the United Kingdom (UK) (*n* = 31 studies, 8.4%), and Spain (*n* = 26 studies, 7.0%). Supplementary Fig. 1 presents the percentage of studies published in national journals for the most frequently studied countries.

When counting studies capturing both single and multiple countries, 105 countries and territories covering all WHO regions were included in the review. Table 1 lists all countries that were represented in the review.

**Table 1:** Countries represented in the study

Country	GNI group	Number of multiple-countries studies	Number of single-country studies	Percentage over the total of single-country studies
Angola	Lower middle income	1	0	0
Argentina	Upper middle income	12	3	0.8
Aruba	High income	1	0	0
Australia	High income	13	4	1.1
Austria	High income	6	1	0.3
Azerbaijan	Upper middle income	1	0	0
Bangladesh	Lower middle income	1	0	0
Belarus	Upper middle income	1	0	0
Belgium	High income	13	2	0.5
Bosnia and Herzegovina	Upper middle income	1	0	0
Brazil	Upper middle income	14	4	1.1
Bulgaria	Upper middle income	2	0	0
Bhutan	Low income	1	0	0
Cameroon	Lower middle income	1	0	0
Canada	High income	29	14	3.8
Chile	High income	4	1	0.3
China	Upper middle income	25	17	4.6
Hong Kong	Lower middle income	3	3	0.8
Taiwan, China	Low income	1	0	0
Colombia	Upper middle income	7	0	0
Costa Rica	Upper middle income	3	0	0
Croatia	High income	7	0	0
Cyprus	High income	2	0	0
Czech Republic	High income	5	0	0
Denmark	High income	9	0	0
Ecuador	Upper middle income	2	0	0
Egypt	Lower middle income	5	0	0
Estonia	High income	3	0	0
Finland	High income	7	1	0.3
France	High income	23	9	2.4
Georgia	Upper middle income	2	0	0
Germany	High income	27	15	4.1
Ghana	Lower middle income	1	1	0.3
Greece	High income	7	0	0
Guatemala	Upper middle income	1	0	0
Honduras	Lower middle income	1	0	0
Hungary	High income	3	0	0
India	Lower middle income	21	11	3
Indonesia	Upper middle income	5	2	0.5
Iran	Upper middle income	6	4	1.1
Iraq	Upper middle income	2	0	0
Ireland	High income	13	5	1.4
Israel	High income	4	0	0
Italy	High income	68	53	14.4
Jamaica	Upper middle income	1	0	0
Japan	High income	6	2	0.5
Kazakhstan	Upper middle income	3	0	0
Kenya	Lower middle income	1	0	0
Kosovo	Upper middle income	1	0	0
Kuwait	High income	3	1	0.3
Kyrgyzstan	Lower middle income	2	0	0
Laos	Lower middle income	1	0	0
Latvia	High income	3	0	0
Lebanon	Upper middle income	1	0	0
Lithuania	High income	6	1	0.3
Luxembourg	High income	1	0	0
Malaysia	Upper middle income	7	2	0.5
Maldives	Upper middle income	1	0	0
Malta	High income	3	0	0
Mexico	Upper middle income	7	0	0
Moldova	Lower middle income	1	0	0
Montenegro	Upper middle income	1	1	0.3
Myanmar	Lower middle income	2	0	0
Nepal	Lower middle income	1	0	0
New Zealand	High income	3	1	0.3
Nigeria	Lower middle income	4	0	0
North Macedonia	High income	4	0	0
Norway	High income	9	3	0.8
Oman	High income	3	1	0.3
Pakistan	Lower middle income	4	1	0.3
Panama	High income	1	0	0
Peru	Upper middle income	2	0	0
Philippines	Lower middle income	6	2	0.5
Poland	High income	9	1	0.3
Portugal	High income	10	0	0
Qatar	High income	1	0	0
Romania	High income	6	0	0
Russian federation	Upper middle income	5	0	0
Samoa	Upper middle income	1	0	0
Saudi Arabia	High income	9	4	1.1
Serbia	Upper middle income	3	0	0
Singapore	High income	5	2	0.5
Slovakia	High income	3	0	0
Slovenia	High income	1	0	0
South Africa	Upper middle income	6	0	0
South Korea	Upper middle income	5	1	0.3
Spain	High income	44	26	7
Sri Lanka	Lower middle income	2	1	0.3
Sweden	High income	10	1	0.3
Switzerland	High income	8	1	0.3
Tanzania	Lower middle income	1	1	0.3
Thailand	Upper middle income	3	1	0.3
The Netherlands	High income	12	3	0.8
Trinidad and Tobago	High income	1	0	0
Tunisia	Lower middle income	2	0	0
Turkey	Upper middle income	5	1	0.3
Ukraine	Lower middle income	3	0	0
United Arab Emirates	High income	3	0	0
United Kingdom	High income	47	31	8.4
United States of America	High income	114	94	25.5
Uruguay	High income	1	0	0
Venezuela	Lower middle income	2	0	0
Viet Nam	Lower middle income	2	0	0
Zambia	Lower middle income	1	1	0.3
Zimbabwe	Lower middle income	1	0	0
Multiple countries	NA	0	35	6.2

NA: Not applicable

Of all the represented countries, only two were low-income countries (1.9%); the majority were high-income countries (*n* = 47, 44.8%), followed by upper-middle-income countries (*n* = 31, 29.5%) and lower-middle-income countries in 26 (24.7%). Figure 2 represents the proportion of studies per GNI category

#### Studied subspecialties

**Fig. 3:**
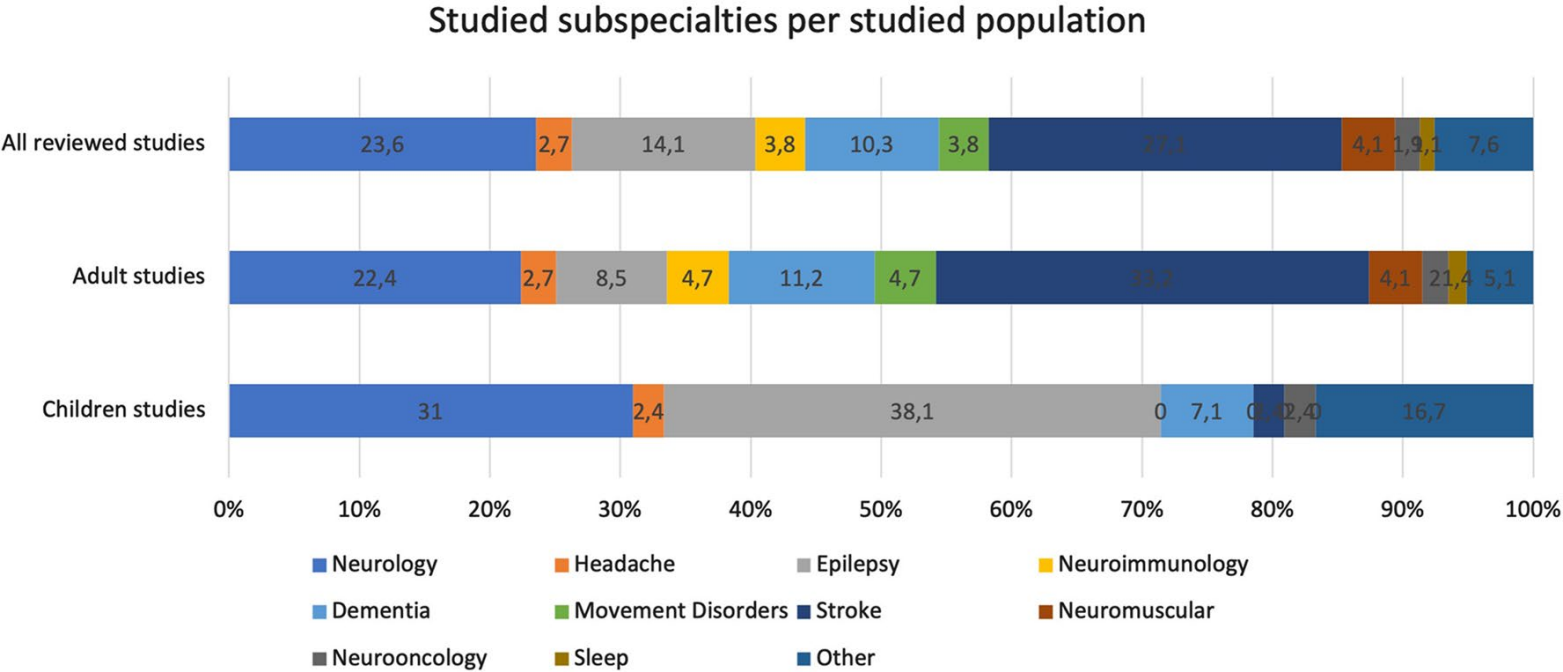
Neurological subspecialties studied (expressed as a proportion of all studies), broken down by all included studies versus subsamples of studies focussing on adult and children’s populations

There were 87 (23.6%) studies that described results across all neurological subspecialties (Fig. 3). Of studies focusing on a single subspecialty, vascular neurology was the most frequently studied (*n* = 100 studies, 27.1%), followed by epilepsy (*n* = 52 studies, 14.1%), and cognitive neurology (*n* = 38 studies, 10.3%). There were 169 different journals, with *Epilepsy Behaviour* being the most represented (*n* = 28 publications, 7.6% of total publications), followed by *Journal of Stroke and Cerebrovascular Diseases* (*n* = 21 publications, 5.7%), and *Stroke* (*n* = 16, 4.3%). The full list of journals is available in the supplementary Table 1.

#### Study aim, design and setting

One hundred and forty-five (39.3%) publications focused on service disruptions, 129 (35.0%) on mitigation strategies and 95 publications (25.7%) on both. The most frequent study design was cross-sectional (*n* = 103, 27.9% publications), followed by the description of an implemented protocol (*n* = 99, 26.8%), before–after studies (*n* = 97, 26.3%), case series (*n* = 57, 15.4%), prospective cohort studies (*n* = 10, 2.7%), and retrospective cohort studies (*n* = 3, 0.8%). The most common study setting was the outpatient setting (*n* = 187, 50.7%), followed by emergency care (*n* = 105, 28.5%), inpatient setting (*n* = 38, 10.3%), and multiple settings (*n* = 38, 10.3%). The setting was unclear in one (0.3%) study.

### Analyses of reported service disruption, causes of disruption and mitigation strategies

#### Service disruption

**Fig. 4:**
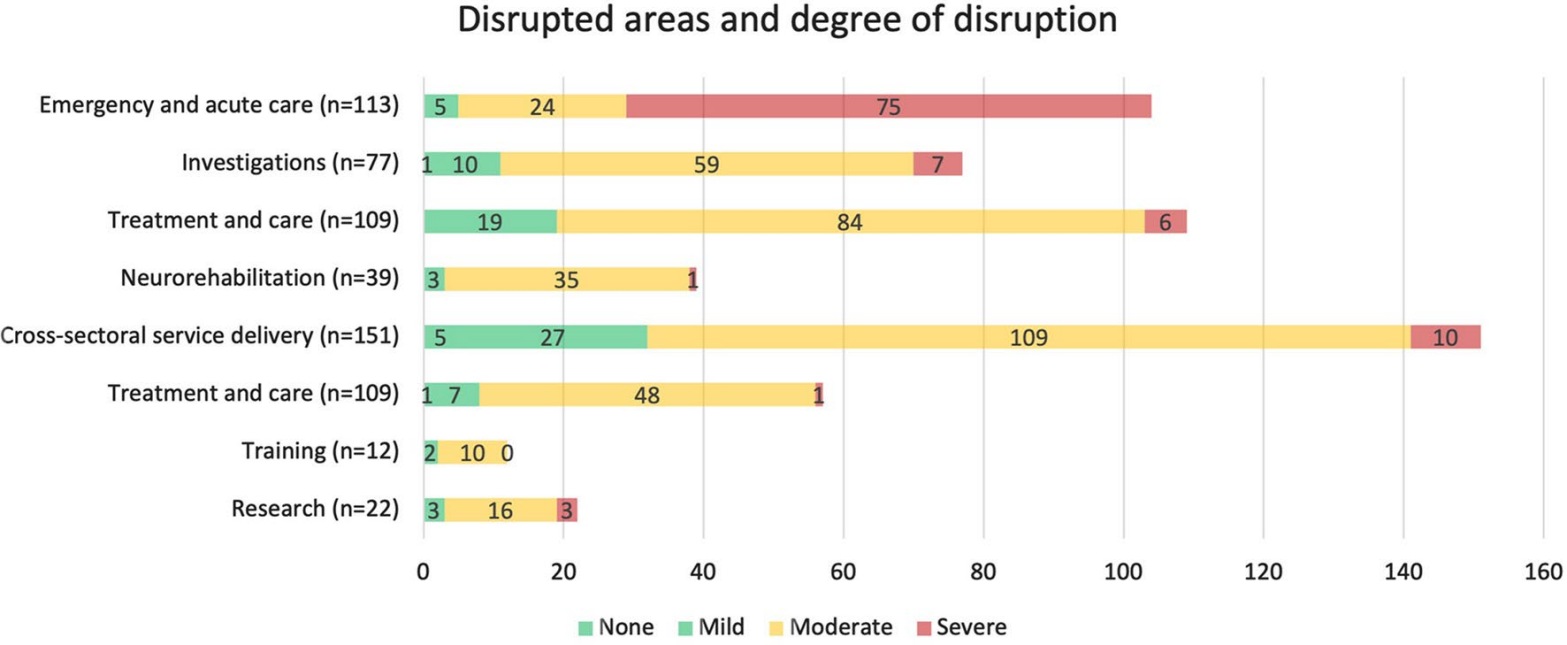
Number of studies that analysed each area of disruption and the described level of disruption per study:

The most frequently reported disruptions occurred for cross-sectoral service delivery for neurological disorders, which was assessed in 151 of 240 studies (62.9%), followed by emergency and acute care for neurological disorders (*n* = 113, 47.1%), and treatment and care for neurological disorders (*n* = 109, 45.4%). The degree of disruption of neurological services was described in 188 studies and was most frequently classified as moderate disruption (*n* = 131, 69.7%), followed by mild disruption (*n* = 40, 21.3%), severe disruption (*n* = 10, 5.3%), and non-disrupted (*n* = 7, 3.7%). Figure 4 depicts the number of studies per analyzed area of disruption, according to the degree of disruption that they described.

#### Causes of disruption

The most frequently described reasons for service disruption were travel restrictions due to lockdowns, national guidelines or local restriction policies (*n* = 196, 81.7%), closure of inpatient and outpatient services or consultations as per health authority directive (*n* = 157, 65.4%), and decrease in outpatient volume due to patients not presenting (*n* = 135, 56.2%).

Table 2 describes the main reasons for disruption and the percentage over the total of studies assessing disruption (n = 240), disaggregated also for high- versus low- and middle-income countries.

**Table 2:** Causes of service disruption described in the studies

Reason of the disruption	Number of studies (*n* = 240) (%)	Studies focused on adults (*n* = 184) (%)	Studies focused on children (*n* = 28) (%)	Studies from HICs (*n* = 180) (%)	Studies from LMICs (*n* = 30) (%)
Travel restrictions hindering patient access to health facilities	196 (81.7%)	149 (81.0%)	26 (92.9%)	146 (81.1%)	25 (83.3%)
Closure of inpatient and outpatient services or consultations as per health authority directive	157 (65.4%)	120 (65.2%)	23 (82.1%)	114 (63.3%)	20 (66.7%)
Decrease in outpatient volume due to patients not presenting	135 (56.2%)	113(61.4%)	13 (46.4%)	112 (62.2%)	15 (50%)
Decreased volume of patients due to cancellation of elective care	109 (45.4%)	79 (42.9%)	17 (60.7%)	77 (42.8%)	11 (36.7%)
Inpatient services and or hospital beds not available	52 (21.7%)	37 (20.1%)	7 (25.0%)	30 (16.7%)	7 (23.3%)
Clinical staff deployed and tasks shifted to provide COVID-19 clinical management or emergency support	40 (16.7%)	31 (16.8%)	3 (10.7%)	25 (19.2%)	5 (16.7%)
Unavailability or stock out of essential medicines, medical diagnostics or other health products at health facilities	40 (16.7%)	29 (15.8%)	3 (1.07%)	22 (12.2%)	7 (23.3%)
Insufficient PPE available for health care providers to provide services	22 (9.2%)	18 (9.8%)	1 (3.6%)	11 (6.1%)	3 (10%)
Insufficient staff to provide services due to staff illness/quarantine	11 (4.6%)	8 (4.3%)	1 (3.6%)	5 (2.8%)	2 (6.7%)

Percentages are calculated over the 240 total studies that analyzed disruption of neurological services, and over the total number of studies that assessed only adults (*n* = 184) or only children (*n* = 28); and over the total number of studies from high-income countries (HICs) (*n* = 180) or low–middle-income countries (LMICs) (*n* = 30)

### Mitigation strategies

The most frequently described mitigation strategies across 224 studies were telemedicine and other telehealth formats (*n* = 184, 82.1%), novel dispensing approaches for medicines (*n* = 116, 51.8%), and redirection of patients (*n* = 95, 42.4%). Table 3 lists the different mitigation strategies and the number of studies reporting each across all studies and disaggregated for high- and low- and middle-income countries.

**Table 3:** Mitigation strategies reported in included studies

Mitigation strategies	Number of studies (*n* = 224) (%)	Studies focused on adults (*n* = 173) (%)	Studies focused on children (*n* = 26) (%)	Studies from HICs (*n* = 164) (%)	Studies from LMICs (*n* = 32) (%)
Telemedicine deployment to replace in-person consults or other teleconsultation formats	184 (82.1%)	140 (80.1%)	25 (96.1%)	136 (82.9%)	28 (87.5%)
Novel dispensing approaches for medicines, novel prescribing approaches	116 (51.8%)	86 (49.7%)	18 (69.2%)	84 (51.2%)	17 (53.1%)
Redirection of patients to alternate care sites, reorientation of referral pathways or integration of several services into a single visit	95 (42.4%)	74 (42.8%)	13 (50%)	68 (41.5%)	14 (43.7%)
Catch-up campaigns for missed appointments	83 (37.1%)	55 (31.8%)	18 (69.2%)	59 (36.0%)	11 (34.4%)
Triaging of neurological patients to identify priorities	57 (25.4%)	45 (26.0%)	6 (23.1%)	42 (25.6%)	9 (28.1%)
Self-care interventions, provision of home-based care or helplines for patients and caregivers	84 (37.5%)	56 (32.4%)	20 (76.9% =	60 (36.6%	14 (43.7%)
Task-shifting or role delegation	44 (19.6%)	34 (19.6%)	5 (19.2%)	34 (18.3%)	4 (12.5%)
Recruitment of additional staff, novel supply chain management and logistics approaches	34 (15.2%)	26 (15.0%)	4 (15.4%)	27 (16.5%)	2 (6.2%)
Community communications to ensure all citizens were aware and informed of continuity of services and that routine care could always be sought	23 (10.3%)	15 (8.7%)	7 (26.9%)	19 (11.6%)	3 (9.4%)
Government removal of user fees	12 (5.4%)	8 (4.6%)	3 (11.5%)	9 (5.5%)	0 (0%)

Percentages are calculated over the 224 studies that described mitigation strategies and over the total number of studies that assessed only adults (*n* = 173) or only children (*n* = 26); and over the total number of studies from high-income countries (HICs) (*n* = 164) or low–middle-income countries (LMICs) (*n* = 32)

## Discussion

This is the first global review of the published evidence regarding the impact the COVID-19 pandemic on the care for people with neurological disorders and the mitigation strategies put in place at policy, system, service level to compensate for service disruptions. The number of studies that addressed these two areas is significant but represents only a small fraction of the total number of studies on COVID-19 and neurology published to date (> 11,000).

We conducted this review in view of existing WHO guidance on maintaining essential services and surveys on service disruption to allow for comparisons and triangulation of results [4, 9]. Despite clear WHO guidance, this extensive review shows that several services and areas of neurology were affected during the pandemic with a deep impact for the care of neurological patients across all areas of service delivery. Service disruptions were particular prominent in cross-sectoral service delivery and emergency and acute neurology care, which was supported by more than 100 studies from different countries and health care system scenarios. Indeed, more than 75% of these studies indicated severe disruption of essential services in acute care—the result of the emerging impact of the first wave of pandemic in different areas of the world. Of interest, also cross-sectoral service delivery, treatment and investigations were affected by the pandemic—with a different impact for patients with chronic neurological conditions, including epilepsy, dementia, neuromuscular, and neuroimmunological disorders.

With respect to causes of service disruptions, the pandemic, on the one hand, introduced heavy travel restrictions for patients and sparked fears of possible infection if attending a healthcare facility—resulting in decreases of patient volumes—reported in more than half of studies. On the other hand, two thirds of studies indicated closure of services due to health authorities and cancellation of elective care. Only a minority of studies reported the need of neurological staff being directly involved in COVID-19 clinical management and emergency support.

Most studies described telemedicine as one of the most important mitigation strategies adopted—but the wide heterogeneity of reports did not allow a specific comparison of applicability and efficacy of different telemedicine approaches in both acute and chronic care. About half of the reviewed studies indicated that novel approaches for drug dispensing and care provision were implemented, such as virtual reality-based rehabilitation or mobile app-based monitoring of patients, but only few studies evaluated the real impact of the mitigation strategies on patient care.

One hundred and five countries and territories are represented in the review. However, the representativeness of the study across the globe, and even within the same country, was limited. To date, no study reported on the situation in a low-income country individually, while four high-income countries (USA, Italy, UK and Spain) accounted for more than 60% of all single-country studies included in this review. This could be due to the fact that the majority of studies focused on the first wave of the pandemic during which these four countries were severely affected [19]. However, the underrepresentation of low- and lower-middle-income countries hinders the analysis of the results based on the income level of the countries. To this end, global studies and surveys are needed to systematically assess the effects of the pandemic and compare the results across different resource settings in an attempt to reduce existing health inequities. After all, what never existed cannot be disrupted, and access to neurological services was lacking in many countries and territories even before the pandemic.

Besides geographic differences, service disruptions and mitigation strategies have notably varied over the course and different waves of the pandemic [20]. So far, the most comparable period is the first wave experienced globally by most countries [21]. After that, the evolution of the pandemic followed different pathways in each country [22], so studies accounting for a single cross-sectional evaluation may not be fully representative of such a dynamic situation [21]. Thus, we were not able to analyze the specific phase of the pandemic wave that studies were evaluating. However, in the present study, 72% of the studies had already been published by the end of October 2020, which could be considered the end of the first wave in most territories according to the WHO observatory [23].

For an adequate interpretation of the results, it should be taken into account the common selective reporting bias [24]. Indeed, the absence of evidence did not equate to the evidence of absence [25] regarding those potentially disrupted areas, but rather reflect the areas that were most frequently prioritized and covered by the published studies. Particularly, neurorehabilitation for both children and adults deserves further investigation. Surprisingly, disruptions of neurorehabilitation services were reported by only 39 of 240 studies. There might be several reasons for this. First, this could be related to the different subspecialties represented in included articles and the timing of the pandemic considered as reporting period [26]. Many studies addressed the issue of vascular neurology, where neurorehabilitation is a keystone in the recovery of patients, but most studies were focused on the disruption related to the acute phase of COVID-19, with very limited information shared about the sub-acute and post-acute phases of care [27]. Second, in many countries, rehabilitation may not be considered within the neurological sub-specialties, being part of different departments, and, therefore, not listed within the affected areas of neurological care, with researchers inadvertently focusing on the aspects of care that they deliver and not always on other areas of their multidisciplinary teamwork [28]. Third, the number of studies that assessed pediatric population was also low, which could also influence the underrepresentation of this service particularly in pediatric populations.

The heterogeneous designs of the works included in this review unfortunately precluded the comparison between the different studies. Less than 10% of the studies were multinational, which also decreases the potential for comparison between different countries and, potentially, their generalizability. There were many publications focusing on guidance and recommendations about *what to do* in terms of alleviating and mitigating the disruption of the neurological services [29, 30], but little consensus on which items should be included in studies systematically evaluating this topic and how the results should be presented. An important lesson from this review is that future studies should clearly define how representative of the studied population and territory the data is, as well as, if possible, how the situation was before the pandemic. In most countries and particularly so in LMICs, neurological services were already very limited before the pandemic [31]. In this specific case, the effects of the pandemic might play a disrupting role on already lacking neurological policies, systems and services and hence the difference between before and after the pandemic may be small, giving a false impression. The uninterrupted access to free medication is essential for many people with neurological disorders worldwide, but especially in LMICs, health policies must ensure the access to them even during the toughest periods of the pandemic.

### Outlook and future recommendations

Our comprehensive approach including all types of neurological disorders and every possible area of disruption gives a broad understanding of how neurological services in general were disrupted by the COVID-19 pandemic. Neurological signs/symptoms are frequent manifestations during the acute and more chronic phases of COVID-19; hence healthcare systems must be functional to accommodate patients with neurological disorders related or unrelated to COVID-19 [29].

There is a need for guidance on how to evaluate disruptions and which mitigation strategies should be taken. The methodological structure and results of this review can provide a template for future studies to enhance reproducibility, comparability, and generalizability of results, particularly with regards to specific outcome measurements. This review should also encourage researchers, public health officials and other relevant stakeholders from LMICs to collect and publish data on service delivery and mitigation strategies in case of disruption to overcome data scarcity and to bring the vulnerabilities and subsequent needs for clinical neurological services in LMICs to the forefront of local, regional and global decision makers. The impact of service disruption on mortality or disability needs also further evaluation. The mortality rates clearly exceeded these from preceding years (2, 3), which gives an estimation of the consequences of the pandemic; however, the total burden attributable to the service disruption still needs specific analyses.

## Conclusion

The COVID-19 pandemic has severely affected all aspects of care of patients with neurological disorders, be it in acute, post-acute, or long-term settings, diagnostic, therapeutic, or rehabilitative. Most of the published evidence describes a moderate to severe disruption of specific neurological services. Given the large number of people living with neurological conditions worldwide, this finding is devastating. The impact of the pandemic on neurological services and neurological disorders may be explained by travel restrictions for patients, fear of infections or closure of inpatient and outpatient services as per health authority directive, amongst others. Authors described various potential strategies to mitigate the effects of the pandemic, with telemedicine being the most frequently used mitigation strategy but evidence of their effectiveness in managing neurological disorders remains largely lacking.

## Supplementary Information

Below is the link to the electronic supplementary material.

Supplementary file1 (DOCX 148 kb)

## Data Availability

The full database is available for other researchers upon request to the corresponding author.

## Code availability

The database is coded in SPSS and can be extracted to Excel.
