# Supplementary material for: Disruptions of neurological services, its causes and mitigation strategies during COVID-19: a global review
Source: J Neurol. 2021 May 22;268(11):3947–60. doi: 10.1007/s00415-021-10588-5 (PMC8140556; doi:10.1007/s00415-021-10588-5)
Supplement: Supplementary file 1 — Supplementary file1 (DOCX 148 kb) [file 415_2021_10588_MOESM1_ESM.docx]

**Supplementary appendix:**

1. Search strategy.
2. Study clasification.
3. Supplementary figure 1: Percentage of studies over the total of studies that was published in national journals in the most represented nations.
4. Supplementary table 1: Full list of Journals, as of February 28, 2020.
5. Full list of references.
6. Search strategy:

The outcome terms that were selected for exploring disruption were “disruption related”, “service”, “discontinuation”, “access” and “limitation”; and regarding mitigation, we employed “mitigation strategies”, “telehealth”, “task shifting”, “training”, “workforce” and “remote”. The full list of terms is available in the supplementary appendix. In the case of PubMed, we used the following Medical Subject Headings (MeSH): “Nervous system diseases OR neurology” and “health services administration” which includes all relevant sub-headings for service disruption and mitigation strategies, including topics such as health facility administration, patient care management, delivery of health care, telemedicine, and health services accessibility, among others^17^.

((neurology) OR (nervous system diseases)) AND (health services administration) AND (COVID-19)

((tw:(neuro*)) OR (tw:(neurolog*/$)) OR (tw:(brain)) OR (tw:(CNS)) OR (tw:(nervous system)) OR (tw:(nerv*/$)) OR (tw:(neurological conditions)) OR (tw:(dementia)) OR (tw:(epilepsy)) OR (tw:(stroke)) OR (tw:(multiple sclerosis)) OR (tw:(parkinson's disease)) OR (tw:(movement disorders)) OR (tw:(neurodevelopmental)) OR (tw:(autism)) OR (tw:(migraine)) OR (tw:(headache disorders)) OR (tw:(headache*/$))) AND ((tw:(disruption related)) OR (tw:(service)) OR (tw:(disruption)) OR (tw:(discontinuation)) OR (tw:(access)) OR (tw:(barrier)) OR (tw:(limitation)))

((tw:(neuro*)) OR (tw:(neurology*/$)) OR (tw:(brain)) OR (tw:(CNS)) OR (tw:((central)nervous system)) OR (tw:(nerv*/$)) OR (tw:(neurological conditions)) OR (tw:(dementia)) OR (tw:(epilepsy)) OR (tw:(stroke)) OR (tw:(multiple sclerosis)) OR (tw:(parkinson's disease)) OR (tw:(movement disorders)) OR (tw:(neurodevelopmental)) OR (tw:(autism)) OR (tw:(migraine)) OR (tw:(headache disorders)) OR (tw:(headache*/$))) AND ((tw:(mitigation strategies)) OR (tw:(telemedicine)) OR (tw:(telehealth)) OR (tw:(task shifting)) OR (tw:(training)) OR (tw:(workforce)) OR (tw:(remote)))

1. Methods:

The subspecialty of neurology was classified including neurological disorders such as headache disorders, epilepsy, neuroimmunology, cognitive disorders including dementia or neurodevelopmental disabilities, movement disorders, vascular neurology, neuromuscular diseases, neuro-oncology, sleep medicine and others.

The study design was categorized into prospective cohort; retrospective cohort; “before-after studies”, which included studies comparing a historical control with the same dates from a specific period of 2020; cross-sectional studies; case series, and “protocols'' which included studies that described disruption or mitigation strategies that had been already implemented in a narrative way when the study design was not better accounted for by any other aforementioned category.

1. *Additional analyses:*

We classified the represented countries according to the Gross National Income (GNI) per capita, according to the 2019 World Bank atlas^1^ criteria, into low-income, countries if GNI is $1,035 or less in 2019; lower middle-income economies if GNI per capita is between $1,036 and $4,045; upper middle-income economies are those with a GNI per capita between $4,046 and $12,535; high-income economies are those with a GNI per capita of $12,536 or more.

1. The World Bank Country and Lending Groups. Available on:<https://datahelpdesk.worldbank.org/knowledgebase/articles/906519-world-bank-country-and-lending-groups>. Accessed on April 10, 2021.
2. Supplementary figure 1: Percentage of studies over the total of studies that was published in national journals in the most represented nations.


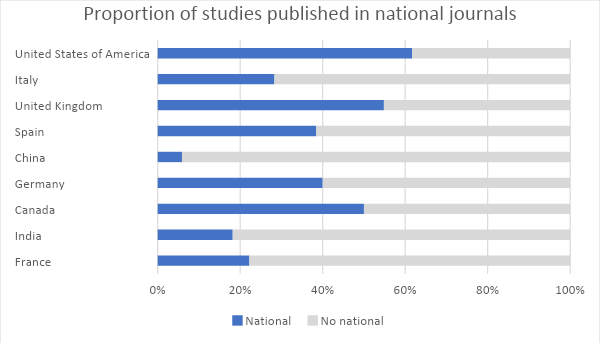
Supplementary table 1: Full list of Journals, as of February 28, 2020, in descending order as of:

|  | Frequency | Percent |
| --- | --- | --- |
| Total | 369 | 100 |
| Epilepsy Behav | 28 | 7.6 |
| J Stroke Cerebrovasc Dis | 21 | 5.7 |
| Stroke | 16 | 4.3 |
| Neurol Sci | 13 | 3.5 |
| Neurology | 11 | 3 |
| J Med Internet Res | 10 | 2.7 |
| Neurologia | 10 | 2.7 |
| World Neurosurg | 9 | 2.4 |
| J Neurointerv Surg | 7 | 1.9 |
| Seizure | 7 | 1.9 |
| Am J Geriatr Psychiatry | 5 | 1.4 |
| Can J Neurol Sci | 5 | 1.4 |
| Eur J Neurol | 5 | 1.4 |
| J Neurol Sci | 5 | 1.4 |
| Mult Scler Relat Disord | 5 | 1.4 |
| Clin Neuropsychol | 4 | 1.1 |
| J Neurol | 4 | 1.1 |
| J Parkinsons Dis | 4 | 1.1 |
| PLoS One | 4 | 1.1 |
| AJNR Am J Neuroradiol | 3 | 0.8 |
| BMJ Open | 3 | 0.8 |
| Epilepsia | 3 | 0.8 |
| Front Neurol | 3 | 0.8 |
| Headache | 3 | 0.8 |
| J Alzheimers Dis | 3 | 0.8 |
| J Intellect Disabil Res | 3 | 0.8 |
| J Neurosurg Anesthesiol | 3 | 0.8 |
| Stroke Vasc Neurol | 3 | 0.8 |
| Acta Neurol Scand | 2 | 0.5 |
| Acta Paediatr | 2 | 0.5 |
| Am J Phys Med Rehabil | 2 | 0.5 |
| Ann Clin Transl Neurol | 2 | 0.5 |
| Arch Clin Neuropsychol | 2 | 0.5 |
| BMJ Case Rep | 2 | 0.5 |
| Childs Nerv Syst | 2 | 0.5 |
| Clin Neurol Neurosurg | 2 | 0.5 |
| Clin Res Cardiol | 2 | 0.5 |
| Epileptic Disord | 2 | 0.5 |
| Eur J Phys Rehabil Med | 2 | 0.5 |
| Eur Neurol | 2 | 0.5 |
| Front Psychiatry | 2 | 0.5 |
| Front Public Health | 2 | 0.5 |
| Int J Environ Res Public Health | 2 | 0.5 |
| Ital J Pediatr | 2 | 0.5 |
| J Am Geriatr Soc | 2 | 0.5 |
| J Child Neurol | 2 | 0.5 |
| J Headache Pain | 2 | 0.5 |
| Mayo Clin Proc | 2 | 0.5 |
| Mol Genet Metab | 2 | 0.5 |
| Mult Scler | 2 | 0.5 |
| Muscle Nerve | 2 | 0.5 |
| Nervenarzt | 2 | 0.5 |
| Pan Afr Med J | 2 | 0.5 |
| Res Dev Disabil | 2 | 0.5 |
| Telemed J E Health | 2 | 0.5 |
| Acta Myol | 1 | 0.3 |
| Acta Neurochir (Wien) | 1 | 0.3 |
| Aging Ment Health | 1 | 0.3 |
| Am J Manag Care | 1 | 0.3 |
| Amyotroph Lateral Scler Frontotemporal Degener | 1 | 0.3 |
| Ann N Y Acad Sci | 1 | 0.3 |
| Ann Phys Rehabil Med | 1 | 0.3 |
| Ann Rheum Dis | 1 | 0.3 |
| Appl Clin Inform | 1 | 0.3 |
| Appl Neuropsychol Adult | 1 | 0.3 |
| Arch Dis Child | 1 | 0.3 |
| Arch Iran Med | 1 | 0.3 |
| Arch Pathol Lab Med | 1 | 0.3 |
| Arch Phys Med Rehabil | 1 | 0.3 |
| Arch Rehab Res Clin Transl | 1 | 0.3 |
| Arq Neuropsiquiatr | 1 | 0.3 |
| Blood Cells Mol Dis | 1 | 0.3 |
| BMC Geriatr | 1 | 0.3 |
| BMJ Health Care Inform | 1 | 0.3 |
| Bone Joint J | 1 | 0.3 |
| Br J Gen Pract | 1 | 0.3 |
| Brain Behav | 1 | 0.3 |
| Brain Behav Immun | 1 | 0.3 |
| Cerebrovasc Dis | 1 | 0.3 |
| Chest | 1 | 0.3 |
| Child Neuropsychol | 1 | 0.3 |
| Circ Cardiovasc Qual Outcomes | 1 | 0.3 |
| Clin Neurophysiol | 1 | 0.3 |
| Clin Neuroradiol | 1 | 0.3 |
| Clin Radiol | 1 | 0.3 |
| CNS Neurosci Ther | 1 | 0.3 |
| Cureus | 1 | 0.3 |
| Dev Med Child Neurol | 1 | 0.3 |
| Disabil Health J | 1 | 0.3 |
| Disabil Rehabil | 1 | 0.3 |
| Emerg Med Australas | 1 | 0.3 |
| Emergencias | 1 | 0.3 |
| Encephale | 1 | 0.3 |
| eNeurologicalSci | 1 | 0.3 |
| Epilepsy Res | 1 | 0.3 |
| Eur Geriatr Med | 1 | 0.3 |
| Eur J Emerg Med | 1 | 0.3 |
| Eur Psychiatry | 1 | 0.3 |
| Eur Respir J | 1 | 0.3 |
| Eur Stroke J | 1 | 0.3 |
| Future Healthc J | 1 | 0.3 |
| IEEE Trans Neural Syst Rehabil Eng | 1 | 0.3 |
| Indian J Ophthalmol | 1 | 0.3 |
| Int J Geriatr Psychiatry | 1 | 0.3 |
| Int J Psychiatry | 1 | 0.3 |
| Int J Rehabil Res | 1 | 0.3 |
| Int J Stroke | 1 | 0.3 |
| Int Psychogeriatr | 1 | 0.3 |
| Intern Emerg Med | 1 | 0.3 |
| J Am Med Dir Assoc | 1 | 0.3 |
| J Clin Neurosci | 1 | 0.3 |
| J Clin Sleep Med | 1 | 0.3 |
| J Cross Cult Gerontol | 1 | 0.3 |
| J Emerg Med | 1 | 0.3 |
| J Glob Health | 1 | 0.3 |
| J Health Serv Res Policy | 1 | 0.3 |
| J Healthc Manag | 1 | 0.3 |
| J Hosp Infect | 1 | 0.3 |
| J Laryngol Otol | 1 | 0.3 |
| J Med Assoc Thai | 1 | 0.3 |
| J Neural Transm (Vienna) | 1 | 0.3 |
| J Neuroimaging | 1 | 0.3 |
| J Neurol Phys Ther | 1 | 0.3 |
| J Neurooncol | 1 | 0.3 |
| J Neuroophthalmol | 1 | 0.3 |
| J Paediatr Child Health | 1 | 0.3 |
| J Pediatr Rehabil Med | 1 | 0.3 |
| J Speech Lang Hear Res | 1 | 0.3 |
| J Telemed Telecare | 1 | 0.3 |
| J Thromb Thrombolysis | 1 | 0.3 |
| JAMA | 1 | 0.3 |
| JAMA Intern Med | 1 | 0.3 |
| JAMA Netw Open | 1 | 0.3 |
| JMIR Hum Factors | 1 | 0.3 |
| Kesmas-National Public Health Journal | 1 | 0.3 |
| Lancet | 1 | 0.3 |
| Lancet Digit Health | 1 | 0.3 |
| Lancet Psychiatry | 1 | 0.3 |
| Lancet Public Health | 1 | 0.3 |
| Medicina (B Aires) | 1 | 0.3 |
| Medicina (Kaunas) | 1 | 0.3 |
| MMWR Morb Mortal Wkly Rep | 1 | 0.3 |
| Mov Disord | 1 | 0.3 |
| N Z Med J | 1 | 0.3 |
| Neuro Oncol | 1 | 0.3 |
| Neuro-Oncol Adv | 1 | 0.3 |
| Neuro-Oncology Practice | 1 | 0.3 |
| Neurodegener Dis Manag | 1 | 0.3 |
| Neurohospitalist | 1 | 0.3 |
| Neurol India | 1 | 0.3 |
| Neurology and Clinical Neuroscience | 1 | 0.3 |
| Pain Physician | 1 | 0.3 |
| Pain Pract | 1 | 0.3 |
| Parkinsonism Relat Disord | 1 | 0.3 |
| Patient Educ Couns | 1 | 0.3 |
| Phys Ther | 1 | 0.3 |
| Postgrad Med J | 1 | 0.3 |
| Pract Neurol | 1 | 0.3 |
| Rheumatol Int | 1 | 0.3 |
| Rheumatology (Oxford) | 1 | 0.3 |
| Scand J Trauma Resusc Emerg Med | 1 | 0.3 |
| Spinal Cord Ser Cases | 1 | 0.3 |
| Stereotact Funct Neurosurg | 1 | 0.3 |
| Sultan Qaboos Univ Med J | 1 | 0.3 |
| Swiss Med Wkly | 1 | 0.3 |
| The Clinical Neuropsychologist | 1 | 0.3 |
| Wiad Lek | 1 | 0.3 |
| Wien Klin Wochenschr | 1 | 0.3 |
| Winsconsin Medical Journal WMJ | 1 | 0.3 |
| Zhonghua Yi Xue Za Zhi | 1 | 0.3 |

**Full list of references:**

- Benaque A, Gurruchaga MJ, Abdelnour C, Hernández I, Cañabate P, Alegret M, Rodríguez I, Rosende-Roca M, Tartari JP, Esteban E, López R, Gil S, Vargas L, Mauleón A, Espinosa A, Ortega G, Sanabria A, Pérez A, Alarcón E, González-Pérez A, Marquié M, Valero S, Tárraga L, Ruiz A, Boada M; Research Center and Memory Clinic, Fundació ACE. Dementia Care in Times of COVID-19: Experience at Fundació ACE in Barcelona, Spain. J Alzheimers Dis. 2020;76(1):33-40. doi: 10.3233/JAD-200547. PMID: 32538856; PMCID: PMC7369075.
- Capozzo R, Zoccolella S, Frisullo ME, Barone R, Dell'Abate MT, Barulli MR, Musio M, Accogli M, Logroscino G. Telemedicine for Delivery of Care in Frontotemporal Lobar Degeneration During COVID-19 Pandemic: Results from Southern Italy. J Alzheimers Dis. 2020;76(2):481-489. doi: 10.3233/JAD-200589. PMID: 32651328.
- Schneider RB, Myers TL, Tarolli CG, Amodeo K, Adams JL, Jensen-Roberts S, Dorsey ER. Remote Administration of the MDS-UPDRS in the Time of COVID-19 and Beyond. J Parkinsons Dis. 2020;10(4):1379-1382. doi: 10.3233/JPD-202121. PMID: 32675421.
- Crisà FM, Leocata F, Arienti VM, Picano M, Berta L, Mainardi HS, Monti AF, Musca F, Colombo S, Palazzi M, La Camera A. Gamma Knife Radiosurgery for Treatment of Brain Metastases during the COVID-19 Outbreak. Stereotact Funct Neurosurg. 2020;98(5):319-323. doi: 10.1159/000510271. Epub 2020 Jul 29. PMID: 32726792; PMCID: PMC7490500.
- Sennott B, Woo K, Hess S, Mitchem D, Klostermann EC, Myrick E, Savica R, Fleisher JE. Novel Outreach Program and Practical Strategies for Patients with Parkinsonism in the COVID-19 Pandemic. J Parkinsons Dis. 2020;10(4):1383-1388. doi: 10.3233/JPD-202156. PMID: 32804103; PMCID: PMC7606485.
- Tarolli CG, Andrzejewski K, Zimmerman GA, Bull M, Goldenthal S, Auinger P, O'Brien M, Dorsey ER, Biglan K, Simuni T. Feasibility, Reliability, and Value of Remote Video-Based Trial Visits in Parkinson's Disease. J Parkinsons Dis. 2020;10(4):1779-1786. doi: 10.3233/JPD-202163. PMID: 32894251.
- Brown EG, Chahine LM, Goldman SM, Korell M, Mann E, Kinel DR, Arnedo V, Marek KL, Tanner CM. The Effect of the COVID-19 Pandemic on People with Parkinson's Disease. J Parkinsons Dis. 2020;10(4):1365-1377. doi: 10.3233/JPD-202249. PMID: 32925107; PMCID: PMC7683050.
- Abdelnour C, Esteban de Antonio E, Pérez-Cordón A, Lafuente A, Buendía M, Pancho A, Jofresa S, Aguilera N, Ibarria M, Cuevas R, Cañada L, Calvet A, Diego S, González-Pérez A, Orellana A, Montrreal L, de Jorge L, Marquié M, Benaque A, Gurruchaga M, Tárraga L, Ruiz A, Boada M; Research Center and Memory Clinic, Fundació ACE. Managing Clinical Trials for Alzheimer's Disease During the COVID-19 Crisis: Experience at Fundació ACE in Barcelona, Spain. J Alzheimers Dis. 2020;77(4):1805-1813. doi: 10.3233/JAD-200750. PMID: 32986671; PMCID: PMC7683041.
- McKenna MC, Al-Hinai M, Bradley D, Doran E, Hunt I, Hutchinson S, Langan Y, O'Rourke D, Qasem R, Redmond J, Troy E, Doherty CP. Patients' Experiences of Remote Neurology Consultations during the COVID-19 Pandemic. Eur Neurol. 2020;83(6):622-625. doi: 10.1159/000511900. Epub 2020 Nov 4. PMID: 33147591; PMCID: PMC7801972.
- Thomas SP, Hardesty CK, Buxton KA, Collins AB, Pruente J, Pham KLD, Sheriko J, McClanahan ME, Inanoglu D, Srinivasan R, Ridnour L, Cooper R, Khurana S, Network TPI. Pediatric intrathecal baclofen management during the COVID-19 pandemic in the US and Canada. J Pediatr Rehabil Med. 2020;13(3):379-384. doi: 10.3233/PRM-200755. PMID: 33164962.
- Zhang LL, Guo YJ, Lin YP, Hu RZ, Yu JP, Yang J, Wang X. Stroke Care in the First Affiliated Hospital of Chengdu Medical College during the COVID-19 Outbreak. Eur Neurol. 2020;83(6):630-635. doi: 10.1159/000513097. Epub 2020 Dec 18. PMID: 33341815; PMCID: PMC7801999.
- Waldman G, Mayeux R, Claassen J, Agarwal S, Willey J, Anderson E, Punzalan P, Lichtcsien R, Bell M, Przedborski S, Ulane C, Roberts K, Williams O, Lassman AB, Lennihan L, Thakur KT. Preparing a neurology department for SARS-CoV-2 (COVID-19): Early experiences at Columbia University Irving Medical Center and the New York Presbyterian Hospital in New York City. Neurology. 2020 May 19;94(20):886-891. doi: 10.1212/WNL.0000000000009519. Epub 2020 Apr 6. PMID: 32253352.
- Perin A, Servadei F, DiMeco F; ‘Hub and Spoke’ Lombardy Neurosurgery Group. May we deliver neuro-oncology in difficult times (e.g. COVID-19)? J Neurooncol. 2020 May;148(1):203-205. doi: 10.1007/s11060-020-03496-7. Epub 2020 Apr 10. PMID: 32277379; PMCID: PMC7148000.
- Majersik JJ, Reddy VK. Acute neurology during the COVID-19 pandemic: Supporting the front line. Neurology. 2020 Jun 16;94(24):1055-1057. doi: 10.1212/WNL.0000000000009564. Epub 2020 Apr 13. PMID: 32284363; PMCID: PMC7455331.
- Phillips NA, Chertkow H, Pichora-Fuller MK, Wittich W. Special Issues on Using the Montreal Cognitive Assessment for telemedicine Assessment During COVID-19. J Am Geriatr Soc. 2020 May;68(5):942-944. doi: 10.1111/jgs.16469. Epub 2020 Apr 15. PMID: 32253754.
- Stillman MD, Capron M, Alexander M, Di Giusto ML, Scivoletto G. COVID-19 and spinal cord injury and disease: results of an international survey. Spinal Cord Ser Cases. 2020 Apr 15;6(1):21. doi: 10.1038/s41394-020-0275-8. PMID: 32296046; PMCID: PMC7156806.
- Bonavita S, Tedeschi G, Atreja A, Lavorgna L. Digital triage for people with multiple sclerosis in the age of COVID-19 pandemic. Neurol Sci. 2020 May;41(5):1007-1009. doi: 10.1007/s10072-020-04391-9. Epub 2020 Apr 17. PMID: 32303856; PMCID: PMC7162735.
- Levy J, Léotard A, Lawrence C, Paquereau J, Bensmail D, Annane D, Delord V, Lofaso F, Bessis S, Prigent H. A model for a ventilator-weaning and early rehabilitation unit to deal with post-ICU impairments following severe COVID-19. Ann Phys Rehabil Med. 2020 Jul;63(4):376-378. doi: 10.1016/j.rehab.2020.04.002. Epub 2020 Apr 18. PMID: 32315800; PMCID: PMC7165266.
- Grandas F, García Domínguez JM, Díaz Otero F; en nombre del Servicio de Neurología del Hospital General Universitario Gregorio Marañón. A neurology department at a tertiary-level hospital during the COVID-19 pandemic. Neurologia. 2020 May;35(4):267-268. English, Spanish. doi: 10.1016/j.nrl.2020.04.005. Epub 2020 Apr 18. PMID: 32364117; PMCID: PMC7166024.
- Giorgi PD, Villa F, Gallazzi E, Debernardi A, Schirò GR, Crisà FM, Talamonti G, D'Aliberti G. The management of emergency spinal surgery during the COVID-19 pandemic in Italy. Bone Joint J. 2020 Jun;102-B(6):671-676. doi: 10.1302/0301-620X.102B6.BJJ-2020-0537. Epub 2020 Apr 23. PMID: 32323563; PMCID: PMC7241059.
- Rodríguez-Pardo J, Fuentes B, Alonso de Leciñana M, Campollo J, Calleja Castaño P, Carneado Ruiz J, Egido Herrero J, García Leal R, Gil Núñez A, Gómez Cerezo JF, Martín Martínez A, Masjuán Vallejo J, Palomino Aguado B, Riera López N, Simón de Las Heras R, Vivancos Mora J, Díez Tejedor E; en nombre del Grupo Multidisciplinar del Plan Ictus Madrid. Acute stroke care during the COVID-19 pandemic. Ictus Madrid Program recommendations. Neurologia. 2020 May;35(4):258-263. English, Spanish. doi: 10.1016/j.nrl.2020.04.008. Epub 2020 Apr 24. PMID: 32364127; PMCID: PMC7180371.
- Wen J, Qi X, Lyon KA, Liang B, Wang X, Feng D, Huang JH. Lessons from China When Performing Neurosurgical Procedures During the Coronavirus Disease 2019 (COVID-19) Pandemic. World Neurosurg. 2020 Jun;138:e955-e960. doi: 10.1016/j.wneu.2020.04.140. Epub 2020 Apr 25. PMID: 32344132; PMCID: PMC7194527.
- Hernando-Requejo V, Huertas-González N, Lapeña-Motilva J, Ogando-Durán G. The epilepsy unit during the COVID-19 epidemic: The role of telemedicine and the effects of confinement on patients with epilepsy. Neurologia. 2020 May;35(4):274-276. English, Spanish. doi: 10.1016/j.nrl.2020.04.014. Epub 2020 Apr 25. PMID: 32364125; PMCID: PMC7183291.
- Sethi NK. EEG during the COVID-19 pandemic: What remains the same and what is different. Clin Neurophysiol. 2020 Jul;131(7):1462. doi: 10.1016/j.clinph.2020.04.007. Epub 2020 Apr 25. PMID: 32388156; PMCID: PMC7182743.
- Agosti E, Giorgianni A, Pradella R, Locatelli D. Coronavirus Disease 2019 (COVID-19) Outbreak: Single-Center Experience in Neurosurgical and Neuroradiologic Emergency Network Tailoring. World Neurosurg. 2020 Jun;138:548-550. doi: 10.1016/j.wneu.2020.04.141. Epub 2020 Apr 27. PMID: 32353537; PMCID: PMC7184971.
- Hernández Ramos FJ, Palomino García A, Jiménez Hernández MD. Neurology during the pandemic. Is COVID-19 changing the organisation of Neurology Departments? Neurologia. 2020 May;35(4):269-271. English, Spanish. doi: 10.1016/j.nrl.2020.04.009. Epub 2020 Apr 27. PMID: 32364118; PMCID: PMC7183930.
- Grazzi L, Rizzoli P. The Adaptation of Management of Chronic Migraine Patients With Medication Overuse to the Suspension of Treatment Protocols During the COVID-19 Pandemic: Lessons From a Tertiary Headache Center in Milan, Italy. Headache. 2020 Jul;60(7):1463-1464. doi: 10.1111/head.13825. Epub 2020 Apr 30. PMID: 32352569; PMCID: PMC7267132.
- Phillips CD, Shatzkes DR, Moonis G, Hsu KA, Doshi A, Filippi CG. From the Eye of the Storm: Multi-Institutional Practical Perspectives on Neuroradiology from the COVID-19 Outbreak in New York City. AJNR Am J Neuroradiol. 2020 Jun;41(6):960-965. doi: 10.3174/ajnr.A6565. Epub 2020 Apr 30. PMID: 32354706; PMCID: PMC7342753.
- Mensah D, Asampong R, Amuna P, Ayanore MA. COVID-19 effects on national health system response to a local epidemic: the case of cerebrospinal meningitis outbreak in Ghana. Pan Afr Med J. 2020 Apr 30;35(Suppl 2):14. doi: 10.11604/pamj.2020.35.2.23138. PMID: 32528625; PMCID: PMC7266470.
- Meschia JF, Barrett KM, Brown RD Jr, Turan TN, Howard VJ, Voeks JH, Lal BK, Howard G, Brott TG. The CREST-2 experience with the evolving challenges of COVID-19: A clinical trial in a pandemic. Neurology. 2020 Jul 7;95(1):29-36. doi: 10.1212/WNL.0000000000009698. Epub 2020 May 1. PMID: 32358216; PMCID: PMC7371383.
- Grossman SN, Han SC, Balcer LJ, Kurzweil A, Weinberg H, Galetta SL, Busis NA. Rapid implementation of virtual neurology in response to the COVID-19 pandemic. Neurology. 2020 Jun 16;94(24):1077-1087. doi: 10.1212/WNL.0000000000009677. Epub 2020 May 1. PMID: 32358217.
- Fazzi E, Galli J. New clinical needs and strategies for care in children with neurodisability during COVID-19. Dev Med Child Neurol. 2020 Jul;62(7):879-880. doi: 10.1111/dmcn.14557. Epub 2020 May 2. PMID: 32358977; PMCID: PMC7267576.
- Ali A. Delay in OnabotulinumtoxinA Treatment During the COVID-19 Pandemic-Perspectives from a Virus Hotspot. Headache. 2020 Jun;60(6):1183-1186. doi: 10.1111/head.13830. Epub 2020 May 2. PMID: 32359098; PMCID: PMC7267266.
- Schirinzi T, Cerroni R, Di Lazzaro G, Liguori C, Scalise S, Bovenzi R, Conti M, Garasto E, Mercuri NB, Pierantozzi M, Pisani A, Stefani A. Self-reported needs of patients with Parkinson's disease during COVID-19 emergency in Italy. Neurol Sci. 2020 Jun;41(6):1373-1375. doi: 10.1007/s10072-020-04442-1. Epub 2020 May 3. PMID: 32363506; PMCID: PMC7196180.
- Schwamm LH, Estrada J, Erskine A, Licurse A. Virtual care: new models of caring for our patients and workforce. Lancet Digit Health. 2020 Jun;2(6):e282-e285. doi: 10.1016/S2589-7500(20)30104-7. Epub 2020 May 6. Erratum in: Lancet Digit Health. 2020 Jun;2(6):e292. PMID: 32382724; PMCID: PMC7202848.
- Li J, Zhang Q, Fang X, Li N, Hu C, Lin Z, Xiong N. Emergent hospital reform in response to outbreak of COVID-19. Brain Behav Immun. 2020 Aug;88:954-955. doi: 10.1016/j.bbi.2020.05.016. Epub 2020 May 6. PMID: 32387512; PMCID: PMC7202825.
- Naccarato M, Scali I, Olivo S, Ajčević M, Buoite Stella A, Furlanis G, Lugnan C, Caruso P, Peratoner A, Cominotto F, Manganotti P. Has COVID-19 played an unexpected "stroke" on the chain of survival? J Neurol Sci. 2020 Jul 15;414:116889. doi: 10.1016/j.jns.2020.116889. Epub 2020 May 6. PMID: 32416370; PMCID: PMC7201240.
- Rai AT, Frei D. A rationale and framework for seeking remote electronic or phone consent approval in endovascular stroke trials - special relevance in the COVID-19 environment and beyond. J Neurointerv Surg. 2020 Jul;12(7):654-657. doi: 10.1136/neurintsurg-2020-016221. Epub 2020 May 7. PMID: 32381522; PMCID: PMC7246108.
- Urbach H, Janssen H, Linn J, Hoffmann T, Tritt S, Weber W, Wiesmann M. Notfällige Neurointerventionen, Covid-19 und Thorax-CT: SOP und Literaturübersicht [Acute Neurointerventions, Covid-19 and Chest-CT: SOP and Literature Review]. Clin Neuroradiol. 2020 Sep;30(3):447-452. German. doi: 10.1007/s00062-020-00911-4. Epub 2020 May 7. PMID: 32382877; PMCID: PMC7204189.
- Hong Z, Li N, Li D, Li J, Li B, Xiong W, Lu L, Li W, Zhou D. Telemedicine During the COVID-19 Pandemic: Experiences From Western China. J Med Internet Res. 2020 May 8;22(5):e19577. doi: 10.2196/19577. PMID: 32349962; PMCID: PMC7212818.
- Wang X, Chen Y, Li Z, Wang D, Wang Y. Providing uninterrupted care during COVID-19 pandemic: experience from Beijing Tiantan Hospital. Stroke Vasc Neurol. 2020 Jun;5(2):180-184. doi: 10.1136/svn-2020-000400. Epub 2020 May 8. PMID: 32385131; PMCID: PMC7246102.
- Agarwal S, Sabadia S, Abou-Fayssal N, Kurzweil A, Balcer LJ, Galetta SL. Training in neurology: Flexibility and adaptability of a neurology training program at the epicenter of COVID-19. Neurology. 2020 Jun 16;94(24):e2608-e2614. doi: 10.1212/WNL.0000000000009675. Epub 2020 May 8. PMID: 32385187.
- Meyer D, Meyer BC, Rapp KS, Modir R, Agrawal K, Hailey L, Mortin M, Lane R, Ranasinghe T, Sorace B, von Kleist TD, Perrinez E, Nabulsi M, Hemmen T. A Stroke Care Model at an Academic, Comprehensive Stroke Center During the 2020 COVID-19 Pandemic. J Stroke Cerebrovasc Dis. 2020 Aug;29(8):104927. doi: 10.1016/j.jstrokecerebrovasdis.2020.104927. Epub 2020 May 8. PMID: 32434728; PMCID: PMC7205687.
- Appireddy R, Jalini S, Shukla G, Boissé Lomax L. Tackling the Burden of Neurological Diseases in Canada with Virtual Care During the COVID-19 Pandemic and Beyond. Can J Neurol Sci. 2020 Sep;47(5):594-597. doi: 10.1017/cjn.2020.92. Epub 2020 May 12. PMID: 32394872; PMCID: PMC7270482.
- Sylaja PN, Srivastava MVP, Shah S, Bhatia R, Khurana D, Sharma A, Pandian JD, Kalia K, Sarmah D, Nair SS, Yavagal DR, Bhattacharya P. The SARS-CoV-2/COVID-19 pandemic and challenges in stroke care in India. Ann N Y Acad Sci. 2020 Aug;1473(1):3-10. doi: 10.1111/nyas.14379. Epub 2020 May 12. PMID: 32396683; PMCID: PMC7273096.
- Capra R, Mattioli F. Tele-health in neurology: an indispensable tool in the management of the SARS-CoV-2 epidemic. J Neurol. 2020 Jul;267(7):1885-1886. doi: 10.1007/s00415-020-09898-x. Epub 2020 May 12. PMID: 32399695; PMCID: PMC7216569.
- Holmes JL, Brake S, Docherty M, Lilford R, Watson S. Emergency ambulance services for heart attack and stroke during UK's COVID-19 lockdown. Lancet. 2020 May 23;395(10237):e93-e94. doi: 10.1016/S0140-6736(20)31031-X. Epub 2020 May 14. PMID: 32416787; PMCID: PMC7255139.
- Siegler JE, Heslin ME, Thau L, Smith A, Jovin TG. Falling stroke rates during COVID-19 pandemic at a comprehensive stroke center. J Stroke Cerebrovasc Dis. 2020 Aug;29(8):104953. doi: 10.1016/j.jstrokecerebrovasdis.2020.104953. Epub 2020 May 14. PMID: 32689621; PMCID: PMC7221408.
- Aledo-Serrano Á, Mingorance A, Jiménez-Huete A, Toledano R, García-Morales I, Anciones C, Gil-Nagel A. Genetic epilepsies and COVID-19 pandemic: Lessons from the caregiver perspective. Epilepsia. 2020 Jun;61(6):1312-1314. doi: 10.1111/epi.16537. Epub 2020 May 18. PMID: 32420620; PMCID: PMC7276740.
- Weinberg MS, Patrick RE, Schwab NA, Owoyemi P, May R, McManus AJ, Gerber J, Harper DG, Arnold SE, Forester B. Clinical Trials and Tribulations in the COVID-19 Era. Am J Geriatr Psychiatry. 2020 Sep;28(9):913-920. doi: 10.1016/j.jagp.2020.05.016. Epub 2020 May 19. PMID: 32507686; PMCID: PMC7236727.
- Kerleroux B, Fabacher T, Bricout N, Moïse M, Testud B, Vingadassalom S, Ifergan H, Janot K, Consoli A, Ben Hassen W, Shotar E, Ognard J, Charbonnier G, L'Allinec V, Guédon A, Bolognini F, Marnat G, Forestier G, Rouchaud A, Pop R, Raynaud N, Zhu F, Cortese J, Chalumeau V, Berge J, Escalard S, Boulouis G; SFNR, the ETIS registry, and the JENI-Research Collaborative. Mechanical Thrombectomy for Acute Ischemic Stroke Amid the COVID-19 Outbreak: Decreased Activity, and Increased Care Delays. Stroke. 2020 Jul;51(7):2012-2017. doi: 10.1161/STROKEAHA.120.030373. Epub 2020 May 20. PMID: 32432994.
- Teo KC, Leung WCY, Wong YK, Liu RKC, Chan AHY, Choi OMY, Kwok WM, Leung KK, Tse MY, Cheung RTF, Tsang AC, Lau KK. Delays in Stroke Onset to Hospital Arrival Time During COVID-19. Stroke. 2020 Jul;51(7):2228-2231. doi: 10.1161/STROKEAHA.120.030105. Epub 2020 May 20. PMID: 32432998; PMCID: PMC7258759.
- Sharma A, Maxwell CR, Farmer J, Greene-Chandos D, LaFaver K, Benameur K. Initial experiences of US neurologists in practice during the COVID-19 pandemic via survey. Neurology. 2020 Aug 4;95(5):215-220. doi: 10.1212/WNL.0000000000009844. Epub 2020 May 21. PMID: 32439820.
- De Marchi F, Cantello R, Ambrosini S, Mazzini L; CANPALS Study Group. Telemedicine and technological devices for amyotrophic lateral sclerosis in the era of COVID-19. Neurol Sci. 2020 Jun;41(6):1365-1367. doi: 10.1007/s10072-020-04457-8. Epub 2020 May 21. PMID: 32440978; PMCID: PMC7240164.
- Roy B, Nowak RJ, Roda R, Khokhar B, Patwa HS, Lloyd T, Rutkove SB. Teleneurology during the COVID-19 pandemic: A step forward in modernizing medical care. J Neurol Sci. 2020 Jul 15;414:116930. doi: 10.1016/j.jns.2020.116930. Epub 2020 May 21. PMID: 32460041; PMCID: PMC7241381.
- López-Bravo A, García-Azorín D, Belvís R, González-Oria C, Latorre G, Santos-Lasaosa S, Guerrero-Peral ÁL. Impact of the COVID-19 pandemic on headache management in Spain: an analysis of the current situation and future perspectives. Neurologia. 2020 Jul-Aug;35(6):372-380. English, Spanish. doi: 10.1016/j.nrl.2020.05.006. Epub 2020 May 21. PMID: 32561333; PMCID: PMC7241344.
- Fuentes B, Alonso de Leciñana M, Calleja-Castaño P, Carneado-Ruiz J, Egido-Herrero J, Gil-Núñez A, Masjuán-Vallejo J, Vivancos-Mora J, Rodríguez-Pardo J, Riera-López N, Ximénez-Carrillo Á, Cruz-Culebras A, Gómez-Escalonilla C, Díez-Tejedor E; en representación de los hospitales del Plan Ictus Madrid. Impact of the COVID-19 pandemic on the organisation of stroke care. Madrid Stroke Care Plan. Neurologia. 2020 Jul-Aug;35(6):363-371. English, Spanish. doi: 10.1016/j.nrl.2020.05.007. Epub 2020 May 21. PMID: 32563566; PMCID: PMC7241395.
- Goodman-Casanova JM, Dura-Perez E, Guzman-Parra J, Cuesta-Vargas A, Mayoral-Cleries F. Telehealth Home Support During COVID-19 Confinement for Community-Dwelling Older Adults With Mild Cognitive Impairment or Mild Dementia: Survey Study. J Med Internet Res. 2020 May 22;22(5):e19434. doi: 10.2196/19434. PMID: 32401215; PMCID: PMC7247465.
- Rudilosso S, Laredo C, Vera V, Vargas M, Renú A, Llull L, Obach V, Amaro S, Urra X, Torres F, Jiménez-Fàbrega FX, Chamorro Á. Acute Stroke Care Is at Risk in the Era of COVID-19: Experience at a Comprehensive Stroke Center in Barcelona. Stroke. 2020 Jul;51(7):1991-1995. doi: 10.1161/STROKEAHA.120.030329. Epub 2020 May 22. PMID: 32438895; PMCID: PMC7258755.
- Guevara C, Villa E, Rosas CS, Diaz V, Naves R. Treating patients with multiple sclerosis during the COVID-19 pandemic: Assessing the expert recommendations. Mult Scler Relat Disord. 2020 Aug;43:102224. doi: 10.1016/j.msard.2020.102224. Epub 2020 May 23. PMID: 32464582; PMCID: PMC7245233.
- Romero-Imbroda J, Reyes-Garrido V, Ciano-Petersen NL, Serrano-Castro PJ. Emergency implantation of a teleneurology service at the neuromuscular unit of Hospital Regional de Málaga during the SARS-CoV-2 pandemic. Neurologia. 2020 Jul-Aug;35(6):415-417. English, Spanish. doi: 10.1016/j.nrl.2020.05.008. Epub 2020 May 23. PMID: 32571553; PMCID: PMC7245281.
- Ford T, Curiale G, Nguyen TN, Aparicio H, Hamlyn EK, Gangadhara S, Cervantes-Arslanian AM, Greer D, Romero JR, Shulman JG. Optimization of resources and modifications in acute ischemic stroke care in response to the global COVID-19 pandemic. J Stroke Cerebrovasc Dis. 2020 Aug;29(8):104980. doi: 10.1016/j.jstrokecerebrovasdis.2020.104980. Epub 2020 May 23. PMID: 32689645; PMCID: PMC7245329.
- Monti S, Delvino P, Bellis E, Milanesi A, Brandolino F, Montecucco C. Impact of delayed diagnoses at the time of COVID-19: increased rate of preventable bilateral blindness in giant cell arteritis. Ann Rheum Dis. 2020 Dec;79(12):1658-1659. doi: 10.1136/annrheumdis-2020-217915. Epub 2020 May 24. PMID: 32448783.
- Yang B, Wang T, Chen J, Chen Y, Wang Y, Gao P, Li G, Chen F, Li L, Wang Z, Zhang H, Song H, Ma Q, Jiao L. Impact of the COVID-19 pandemic on the process and outcome of thrombectomy for acute ischemic stroke. J Neurointerv Surg. 2020 Jul;12(7):664-668. doi: 10.1136/neurintsurg-2020-016177. Epub 2020 May 25. PMID: 32451358; PMCID: PMC7276247.
- Kossoff EH, Turner Z, Adams J, Bessone SK, Avallone J, McDonald TJW, Diaz-Arias L, Barron BJ, Vizthum D, Cervenka MC. Ketogenic diet therapy provision in the COVID-19 pandemic: Dual-center experience and recommendations. Epilepsy Behav. 2020 Oct;111:107181. doi: 10.1016/j.yebeh.2020.107181. Epub 2020 May 25. PMID: 32512472; PMCID: PMC7247448.
- Bres Bullrich M, Fridman S, Mandzia JL, Mai LM, Khaw A, Vargas Gonzalez JC, Bagur R, Sposato LA. COVID-19: Stroke Admissions, Emergency Department Visits, and Prevention Clinic Referrals. Can J Neurol Sci. 2020 Sep;47(5):693-696. doi: 10.1017/cjn.2020.101. Epub 2020 May 26. PMID: 32450927; PMCID: PMC7324648.
- Schirmer CM, Ringer AJ, Arthur AS, Binning MJ, Fox WC, James RF, Levitt MR, Tawk RG, Veznedaroglu E, Walker M, Spiotta AM; Endovascular Research Group (ENRG). Delayed presentation of acute ischemic strokes during the COVID-19 crisis. J Neurointerv Surg. 2020 Jul;12(7):639-642. doi: 10.1136/neurintsurg-2020-016299. Epub 2020 May 28. PMID: 32467244; PMCID: PMC7295853.
- Montaner J, Barragán-Prieto A, Pérez-Sánchez S, Escudero-Martínez I, Moniche F, Sánchez-Miura JA, Ruiz-Bayo L, González A. Break in the Stroke Chain of Survival due to COVID-19. Stroke. 2020 Aug;51(8):2307-2314. doi: 10.1161/STROKEAHA.120.030106. Epub 2020 May 29. PMID: 32466738; PMCID: PMC7282408.
- Sarti D, De Salvatore M, Gazzola S, Pantaleoni C, Granocchio E. So far so close: an insight into smart working and telehealth reorganization of a Language and Learning Disorders Service in Milan during COVID-19 pandemic. Neurol Sci. 2020 Jul;41(7):1659-1662. doi: 10.1007/s10072-020-04481-8. Epub 2020 May 29. PMID: 32472517; PMCID: PMC7257356.
- Diestro JDB, Li YM, Parra-Fariñas C, Sarma D, Bharatha A, Marotta TR, Spears J. Letter to the Editor 'Aneurysmal Subarachnoid Hemorrhage: Collateral Damage of COVID?'. World Neurosurg. 2020 Jul;139:744-745. doi: 10.1016/j.wneu.2020.05.206. Epub 2020 May 29. PMID: 32474100; PMCID: PMC7256541.
- Grote L, McNicholas WT, Hedner J; ESADA collaborators. Sleep apnoea management in Europe during the COVID-19 pandemic: data from the European Sleep Apnoea Database (ESADA). Eur Respir J. 2020 Jun 18;55(6):2001323. doi: 10.1183/13993003.01323-2020. PMID: 32366489; PMCID: PMC7236823.
- Cilia R, Mancini F, Bloem BR, Eleopra R. Telemedicine for parkinsonism: A two-step model based on the COVID-19 experience in Milan, Italy. Parkinsonism Relat Disord. 2020 Jun;75:130-132. doi: 10.1016/j.parkreldis.2020.05.038. Epub 2020 Jun 10. PMID: 32723588; PMCID: PMC7286232.
- Mauri E, Abati E, Musumeci O, Rodolico C, D'Angelo MG, Mirabella M, Lucchini M, Bello L, Pegoraro E, Maggi L, Manneschi L, Gemelli C, Grandis M, Zuppa A, Massucco S, Benedetti L, Caponnetto C, Schenone A, Prelle A, Previtali SC, Scarlato M, D'Amico A, Bertini E, Pennisi EM, De Giglio L, Pane M, Mercuri E, Mongini T, Ricci F, Berardinelli A, Astrea G, Lenzi S, Battini R, Ricci G, Torri F, Siciliano G, Santorelli FM, Ariatti A, Filosto M, Passamano L, Politano L, Scutifero M, Tonin P, Fossati B, Panicucci C, Bruno C, Ravaglia S, Monforte M, Tasca G, Ricci E, Petrucci A, Santoro L, Ruggiero L, Barp A, Albamonte E, Sansone V, Gagliardi D, Costamagna G, Govoni A, Magri F, Brusa R, Velardo D, Meneri M, Sciacco M, Corti S, Bresolin N, Moroni I, Messina S, Di Muzio A, Nigro V, Liguori R, Antonini G, Toscano A, Minetti C, Comi GP; Italian Association of Myology. Estimating the impact of COVID-19 pandemic on services provided by Italian Neuromuscular Centers: an Italian Association of Myology survey of the acute phase. Acta Myol. 2020 Jun 1;39(2):57-66. doi: 10.36185/2532-1900-008. PMID: 32904925; PMCID: PMC7460733.
- Pop R, Quenardelle V, Hasiu A, Mihoc D, Sellal F, Dugay MH, Lebedinsky PA, Schluck E, LA Porta A, Courtois S, Gheoca R, Wolff V, Beaujeux R. Impact of the COVID-19 outbreak on acute stroke pathways - insights from the Alsace region in France. Eur J Neurol. 2020 Sep;27(9):1783-1787. doi: 10.1111/ene.14316. Epub 2020 Jun 3. PMID: 32399995; PMCID: PMC7273043.
- Frisullo G, De Belvis AG, Marca GD, Angioletti C, Calabresi P. Stroke integrated care pathway during COVID-19 pandemic. Neurol Sci. 2020 Jul;41(7):1673-1675. doi: 10.1007/s10072-020-04480-9. Epub 2020 Jun 3. PMID: 32495186; PMCID: PMC7267753.
- Pasarikovski CR, da Costa L. The Impact of the Covid-19 Pandemic on Stroke Volume. Can J Neurol Sci. 2020 Nov;47(6):847-848. doi: 10.1017/cjn.2020.116. Epub 2020 Jun 4. PMID: 32493522; PMCID: PMC7327147.
- Andrews JA, Berry JD, Baloh RH, Carberry N, Cudkowicz ME, Dedi B, Glass J, Maragakis NJ, Miller TM, Paganoni S, Rothstein JD, Shefner JM, Simmons Z, Weiss MD, Bedlack RS. Amyotrophic lateral sclerosis care and research in the United States during the COVID-19 pandemic: Challenges and opportunities. Muscle Nerve. 2020 Aug;62(2):182-186. doi: 10.1002/mus.26989. Epub 2020 Jun 5. PMID: 32445195; PMCID: PMC7283687.
- Conde-Blanco E, Centeno M, Tio E, Muriana D, García-Peñas JJ, Serrano P, Nagel AG, Serratosa J, Jiménez ÁP, Toledo M, Donaire A, Manzanares I, Betrán O, Carreño M. Emergency implementation of telemedicine for epilepsy in Spain: Results of a survey during SARS-CoV-2 pandemic. Epilepsy Behav. 2020 Oct;111:107211. doi: 10.1016/j.yebeh.2020.107211. Epub 2020 Jun 5. PMID: 32540769; PMCID: PMC7274642.
- De Silva DA, Tan IF, Thilarajah S. A protocol for acute stroke unit care during the COVID-19 pandemic. J Stroke Cerebrovasc Dis. 2020 Sep;29(9):105009. doi: 10.1016/j.jstrokecerebrovasdis.2020.105009. Epub 2020 Jun 5. PMID: 32807424; PMCID: PMC7274571.
- Dann L, Fitzsimons J, Gorman KM, Hourihane J, Okafor I. Disappearing act: COVID-19 and paediatric emergency department attendances. Arch Dis Child. 2020 Aug;105(8):810-811. doi: 10.1136/archdischild-2020-319654. Epub 2020 Jun 9. PMID: 32518141; PMCID: PMC7316106.
- Rametta SC, Fridinger SE, Gonzalez AK, Xian J, Galer PD, Kaufman M, Prelack MS, Sharif U, Fitzgerald MP, Melamed SE, Malcolm MP, Kessler SK, Stephenson DJ, Banwell BL, Abend NS, Helbig I. Analyzing 2,589 child neurology telehealth encounters necessitated by the COVID-19 pandemic. Neurology. 2020 Sep 1;95(9):e1257-e1266. doi: 10.1212/WNL.0000000000010010. Epub 2020 Jun 9. PMID: 32518152; PMCID: PMC7538222.
- Bertamino M, Cornaglia S, Zanetti A, Di Rocco A, Ronchetti A, Signa S, Severino M, Moretti P; Gaslini Stroke Study Group. Impact on rehabilitation programs during COVID-19 containment for children with pediatric and perinatal stroke. Eur J Phys Rehabil Med. 2020 Oct;56(5):692-694. doi: 10.23736/S1973-9087.20.06407-2. Epub 2020 Jun 9. PMID: 32519529.
- Buonomo A, Brescia Morra V, Zappulo E, Lanzillo R, Gentile I, Montella E, Triassi M, Palladino R, Moccia M. COVID-19 prevention and multiple sclerosis management: The SAFE pathway for the post-peak. Mult Scler Relat Disord. 2020 Sep;44:102282. doi: 10.1016/j.msard.2020.102282. Epub 2020 Jun 10. PMID: 32554288; PMCID: PMC7283048.
- Khot UN, Reimer AP, Brown A, Hustey FM, Hussain MS, Kapadia SR, Svensson LG. Impact of COVID-19 Pandemic on Critical Care Transfers for ST-Segment-Elevation Myocardial Infarction, Stroke, and Aortic Emergencies. Circ Cardiovasc Qual Outcomes. 2020 Aug;13(8):e006938. doi: 10.1161/CIRCOUTCOMES.120.006938. Epub 2020 Jun 11. PMID: 32524835.
- Capozzo R, Zoccolella S, Musio M, Barone R, Accogli M, Logroscino G. Telemedicine is a useful tool to deliver care to patients with Amyotrophic Lateral Sclerosis during COVID-19 pandemic: results from Southern Italy. Amyotroph Lateral Scler Frontotemporal Degener. 2020 Nov;21(7-8):542-548. doi: 10.1080/21678421.2020.1773502. Epub 2020 Jun 12. PMID: 32530314.
- Diegoli H, Magalhães PSC, Martins SCO, Moro CHC, França PHC, Safanelli J, Nagel V, Venancio VG, Liberato RB, Longo AL. Decrease in Hospital Admissions for Transient Ischemic Attack, Mild, and Moderate Stroke During the COVID-19 Era. Stroke. 2020 Aug;51(8):2315-2321. doi: 10.1161/STROKEAHA.120.030481. Epub 2020 Jun 12. PMID: 32530738; PMCID: PMC7302100.
- Sweid A, Jabbour P, Tjoumakaris S. Letter to the Editor: Incidence of Acute Ischemic Stroke and Rate of Mechanical Thrombectomy During the COVID-19 Pandemic in a Large Tertiary Care Telemedicine Network. World Neurosurg. 2020 Aug;140:491-492. doi: 10.1016/j.wneu.2020.06.053. Epub 2020 Jun 13. PMID: 32544621; PMCID: PMC7293475.
- Huang JF, Greenway MRF, Nasr DM, Chukwudelunzu FE Sr, Demaerschalk BM, O'Carroll CB, Nord CA, Pahl EA, Barrett KM, Williams LN. Telestroke in the Time of COVID-19: The Mayo Clinic Experience. Mayo Clin Proc. 2020 Aug;95(8):1704-1708. doi: 10.1016/j.mayocp.2020.06.007. Epub 2020 Jun 13. PMID: 32753143; PMCID: PMC7293444.
- McGinley MP, Ontaneda D, Wang Z, Weber M, Shook S, Stanton M, Bermel R. Teleneurology as a Solution for Outpatient Care During the COVID-19 Pandemic. Telemed J E Health. 2020 Dec;26(12):1537-1539. doi: 10.1089/tmj.2020.0137. Epub 2020 Jun 16. PMID: 32552509; PMCID: PMC7757521.
- Onteddu SR, Nalleballe K, Sharma R, Brown AT. Underutilization of health care for strokes during the COVID-19 outbreak. Int J Stroke. 2020 Jul;15(5):NP9-NP10. doi: 10.1177/1747493020934362. Epub 2020 Jun 18. PMID: 32478606.
- Uchino K, Kolikonda MK, Brown D, Kovi S, Collins D, Khawaja Z, Buletko AB, Russman AN, Hussain MS. Decline in Stroke Presentations During COVID-19 Surge. Stroke. 2020 Aug;51(8):2544-2547. doi: 10.1161/STROKEAHA.120.030331. Epub 2020 Jun 18. PMID: 32716818; PMCID: PMC7309646.
- Sastre-Garriga J, Tintoré M, Montalban X. Keeping standards of multiple sclerosis care through the COVID-19 pandemic. Mult Scler. 2020 Sep;26(10):1153-1156. doi: 10.1177/1352458520931785. Epub 2020 Jun 19. PMID: 32552382.
- Co COC, Yu JRT, Macrohon-Valdez MC, Laxamana LC, De Guzman VPE, Berroya-Moreno RMM, Mariano MM, Rivera PPDP, Racpan-Cauntay JLM, Ilano KCS, Trias EC, Domingo AMC, Marcelo AVB, Pineda-Franks MCC. Acute stroke care algorithm in a private tertiary hospital in the Philippines during the COVID-19 pandemic: A third world country experience. J Stroke Cerebrovasc Dis. 2020 Sep;29(9):105059. doi: 10.1016/j.jstrokecerebrovasdis.2020.105059. Epub 2020 Jun 20. PMID: 32807464; PMCID: PMC7305875.
- Barlinn K, Siepmann T, Pallesen LP, Winzer S, Sedghi A, Schroettner P, Hochauf-Stange K, Prakapenia A, Moustafa H, de With K, Linn J, Reichmann H, Barlinn J, Puetz V. Universal laboratory testing for SARS-CoV-2 in hyperacute stroke during the COVID-19 pandemic. J Stroke Cerebrovasc Dis. 2020 Sep;29(9):105061. doi: 10.1016/j.jstrokecerebrovasdis.2020.105061. Epub 2020 Jun 20. PMID: 32807466; PMCID: PMC7305910.
- Agarwal S, Scher E, Rossan-Raghunath N, Marolia D, Butnar M, Torres J, Zhang C, Kim S, Sanger M, Humbert K, Tanweer O, Shapiro M, Raz E, Nossek E, Nelson PK, Riina HA, de Havenon A, Wachs M, Farkas J, Tiwari A, Arcot K, Parella DT, Liff J, Wu T, Wittman I, Caldwell R, Frontera J, Lord A, Ishida K, Yaghi S. Acute stroke care in a New York City comprehensive stroke center during the COVID-19 pandemic. J Stroke Cerebrovasc Dis. 2020 Sep;29(9):105068. doi: 10.1016/j.jstrokecerebrovasdis.2020.105068. Epub 2020 Jun 20. PMID: 32807471; PMCID: PMC7305900.
- Strasser S, Miskolczi L, Cunha J, Justynski L. COVID-19 Impact on Stroke Presentations. J Stroke Cerebrovasc Dis. 2020 Oct;29(10):105077. doi: 10.1016/j.jstrokecerebrovasdis.2020.105077. Epub 2020 Jun 25. PMID: 32912524; PMCID: PMC7315973.
- Lange SJ, Ritchey MD, Goodman AB, Dias T, Twentyman E, Fuld J, Schieve LA, Imperatore G, Benoit SR, Kite-Powell A, Stein Z, Peacock G, Dowling NF, Briss PA, Hacker K, Gundlapalli AV, Yang Q. Potential Indirect Effects of the COVID-19 Pandemic on Use of Emergency Departments for Acute Life-Threatening Conditions - United States, January-May 2020. MMWR Morb Mortal Wkly Rep. 2020 Jun 26;69(25):795-800. doi: 10.15585/mmwr.mm6925e2. PMID: 32584802; PMCID: PMC7316316.
- Patel PD, Kelly KA, Reynolds RA, Turer RW, Salwi S, Rosenbloom ST, Bonfield CM, Naftel RP. Tracking the Volume of Neurosurgical Care During the Coronavirus Disease 2019 Pandemic. World Neurosurg. 2020 Oct;142:e183-e194. doi: 10.1016/j.wneu.2020.06.176. Epub 2020 Jun 27. PMID: 32599201; PMCID: PMC7319935.
- Perry R, Banaras A, Werring DJ, Simister R. What has caused the fall in stroke admissions during the COVID-19 pandemic? J Neurol. 2020 Dec;267(12):3457-3458. doi: 10.1007/s00415-020-10030-2. Epub 2020 Jun 29. PMID: 32601757; PMCID: PMC7322387.
- Furlanis G, Ajčević M, Naccarato M, Caruso P, Scali I, Lugnan C, Buoite Stella A, Manganotti P. e-Health vs COVID-19: home patient telemonitoring to maintain TIA continuum of care. Neurol Sci. 2020 Aug;41(8):2023-2024. doi: 10.1007/s10072-020-04524-0. Epub 2020 Jun 30. PMID: 32607851; PMCID: PMC7324905.
- Deshmukh AV, Badakere A, Sheth J, Bhate M, Kulkarni S, Kekunnaya R. Pivoting to teleconsultation for paediatric ophthalmology and strabismus: Our experience during COVID-19 times. Indian J Ophthalmol. 2020 Jul;68(7):1387-1391. doi: 10.4103/ijo.IJO_1675_20. PMID: 32587172; PMCID: PMC7574089.
- Yang L, Brown-Johnson CG, Miller-Kuhlmann R, Kling SMR, Saliba-Gustafsson EA, Shaw JG, Gold CA, Winget M. Accelerated launch of video visits in ambulatory neurology during COVID-19: Key lessons from the Stanford experience. Neurology. 2020 Aug 18;95(7):305-311. doi: 10.1212/WNL.0000000000010015. Epub 2020 Jul 1. PMID: 32611634.
- Hajdu SD, Pittet V, Puccinelli F, Ben Hassen W, Ben Maacha M, Blanc R, Bracco S, Broocks G, Bartolini B, Casseri T, Clarençon F, Naggara O, Eugène F, Ferré JC, Guédon A, Houdart E, Krings T, Lehmann P, Limbucci N, Machi P, Macho J, Mandruzzato N, Nappini S, Nawka MT, Nicholson P, Marto JP, Pereira V, Correia MA, Pinho-E-Melo T, Nuno Ramos J, Raz E, Ferreira P, Reis J, Shapiro M, Shotar E, van Horn N, Piotin M, Saliou G. Acute Stroke Management During the COVID-19 Pandemic: Does Confinement Impact Eligibility for Endovascular Therapy? Stroke. 2020 Aug;51(8):2593-2596. doi: 10.1161/STROKEAHA.120.030794. Epub 2020 Jul 1. PMID: 32716828; PMCID: PMC7340133.
- Prasad S, Holla VV, Neeraja K, Surisetti BK, Kamble N, Yadav R, Pal PK. Impact of Prolonged Lockdown due to COVID-19 in Patients with Parkinson's Disease. Neurol India. 2020 Jul-Aug;68(4):792-795. doi: 10.4103/0028-3886.293472. PMID: 32859814.
- Yaeger KA, Fifi JT, Lara-Reyna J, Rossitto C, Ladner T, Yim B, Hardigan T, Maragkos GA, Shigematsu T, Majidi S, Mocco J. Initial Stroke Thrombectomy Experience in New York City during the COVID-19 Pandemic. AJNR Am J Neuroradiol. 2020 Aug;41(8):1357-1360. doi: 10.3174/ajnr.A6652. Epub 2020 Jul 2. PMID: 32616582; PMCID: PMC7658874.
- Dyson EW, Craven CL, Tisdall MM, James GA. The impact of social distancing on pediatric neurosurgical emergency referrals during the COVID-19 pandemic: a prospective observational cohort study. Childs Nerv Syst. 2020 Sep;36(9):1821-1823. doi: 10.1007/s00381-020-04783-4. Epub 2020 Jul 3. PMID: 32621006; PMCID: PMC7333974.
- Viswanathan S. Management of Idiopathic CNS inflammatory diseases during the COVID-19 pandemic: Perspectives and strategies for continuity of care from a South East Asian Center with limited resources. Mult Scler Relat Disord. 2020 Sep;44:102353. doi: 10.1016/j.msard.2020.102353. Epub 2020 Jul 3. PMID: 32653804; PMCID: PMC7341969.
- Frisullo G, Brunetti V, Di Iorio R, Broccolini A, Caliandro P, Monforte M, Morosetti R, Piano C, Pilato F, Calabresi P, Della Marca G; STROKE TEAM Collaborators. Effect of lockdown on the management of ischemic stroke: an Italian experience from a COVID hospital. Neurol Sci. 2020 Sep;41(9):2309-2313. doi: 10.1007/s10072-020-04545-9. Epub 2020 Jul 6. PMID: 32632635; PMCID: PMC7338130.
- Mateen FJ, Rezaei S, Alakel N, Gazdag B, Kumar AR, Vogel A. Impact of COVID-19 on U.S. and Canadian neurologists' therapeutic approach to multiple sclerosis: a survey of knowledge, attitudes, and practices. J Neurol. 2020 Dec;267(12):3467-3475. doi: 10.1007/s00415-020-10045-9. Epub 2020 Jul 7. PMID: 32638107; PMCID: PMC7339100.
- Vaitheswaran S, Lakshminarayanan M, Ramanujam V, Sargunan S, Venkatesan S. Experiences and Needs of Caregivers of Persons With Dementia in India During the COVID-19 Pandemic-A Qualitative Study. Am J Geriatr Psychiatry. 2020 Nov;28(11):1185-1194. doi: 10.1016/j.jagp.2020.06.026. Epub 2020 Jul 7. PMID: 32736918; PMCID: PMC7340037.
- Hsiao J, Sayles E, Antzoulatos E, Stanton RJ, Sucharew H, Broderick JP, Demel SL, Flaherty ML, Grossman AW, Kircher C, Kreitzer N, Peariso K, Prestigiacomo CJ, Shirani P, Walsh KB, Lampton H, Adeoye O, Khatri P. Effect of COVID-19 on Emergent Stroke Care: A Regional Experience. Stroke. 2020 Sep;51(9):e2111-e2114. doi: 10.1161/STROKEAHA.120.030499. Epub 2020 Jul 8. PMID: 32639860; PMCID: PMC7359904.
- Mathiesen T, Arraez M, Asser T, Balak N, Barazi S, Bernucci C, Bolger C, Broekman MLD, Demetriades AK, Feldman Z, Fontanella MM, Foroglou N, Lafuente J, Maier AD, Meyer B, Niemelä M, Roche PH, Sala F, Samprón N, Sandvik U, Schaller K, Thome C, Thys M, Tisell M, Vajkoczy P, Visocchi M; EANS Ethico-legal committee. A snapshot of European neurosurgery December 2019 vs. March 2020: just before and during the Covid-19 pandemic. Acta Neurochir (Wien). 2020 Sep;162(9):2221-2233. doi: 10.1007/s00701-020-04482-8. Epub 2020 Jul 8. PMID: 32642834; PMCID: PMC7343382.
- Qureshi AI, Siddiq F, French BR, Gomez CR, Jani V, Hassan AE, Suri MFK. Effect of COVID-19 Pandemic on Mechanical Thrombectomy for Acute Ischemic Stroke Treatment in United States. J Stroke Cerebrovasc Dis. 2020 Oct;29(10):105140. doi: 10.1016/j.jstrokecerebrovasdis.2020.105140. Epub 2020 Jul 11. PMID: 32912573; PMCID: PMC7834710.
- Esenwa C, Parides MK, Labovitz DL. The effect of COVID-19 on stroke hospitalizations in New York City. J Stroke Cerebrovasc Dis. 2020 Oct;29(10):105114. doi: 10.1016/j.jstrokecerebrovasdis.2020.105114. Epub 2020 Jul 13. PMID: 32912527; PMCID: PMC7355321.
- Hewitt KC, Loring DW. Emory university telehealth neuropsychology development and implementation in response to the COVID-19 pandemic. Clin Neuropsychol. 2020 Oct-Nov;34(7-8):1352-1366. doi: 10.1080/13854046.2020.1791960. Epub 2020 Jul 14. PMID: 32660335.
- Andrade-Campos M, Escuder-Azuara B, de Frutos LL, Serrano-Gonzalo I, Giraldo P; GEEDL; FEETEG; AEEFEG. Direct and indirect effects of the SARS-CoV-2 pandemic on Gaucher Disease patients in Spain: Time to reconsider home-based therapies? Blood Cells Mol Dis. 2020 Nov;85:102478. doi: 10.1016/j.bcmd.2020.102478. Epub 2020 Jul 14. PMID: 32688219; PMCID: PMC7358160.
- Alkhotani A, Siddiqui MI, Almuntashri F, Baothman R. The effect of COVID-19 pandemic on seizure control and self-reported stress on patient with epilepsy. Epilepsy Behav. 2020 Nov;112:107323. doi: 10.1016/j.yebeh.2020.107323. Epub 2020 Jul 14. PMID: 32712565; PMCID: PMC7359799.
- Wirrell EC, Grinspan ZM, Knupp KG, Jiang Y, Hammeed B, Mytinger JR, Patel AD, Nabbout R, Specchio N, Cross JH, Shellhaas RA. Care Delivery for Children With Epilepsy During the COVID-19 Pandemic: An International Survey of Clinicians. J Child Neurol. 2020 Nov;35(13):924-933. doi: 10.1177/0883073820940189. Epub 2020 Jul 15. PMID: 32666891; PMCID: PMC7364331.
- Panda PK, Dawman L, Panda P, Sharawat IK. Feasibility and effectiveness of teleconsultation in children with epilepsy amidst the ongoing COVID-19 pandemic in a resource-limited country. Seizure. 2020 Oct;81:29-35. doi: 10.1016/j.seizure.2020.07.013. Epub 2020 Jul 18. PMID: 32712376; PMCID: PMC7368411.
- Rinkel LA, Prick JCM, Slot RER, Sombroek NMA, Burggraaff J, Groot AE, Emmer BJ, Roos YBWEM, Brouwer MC, van den Berg-Vos RM, Majoie CBLM, Beenen LFM, van de Beek D, Visser MC, van Schaik SM, Coutinho JM. Impact of the COVID-19 outbreak on acute stroke care. J Neurol. 2021 Feb;268(2):403-408. doi: 10.1007/s00415-020-10069-1. Epub 2020 Jul 20. PMID: 32691235; PMCID: PMC7370633.
- Ikenberg B, Hemmer B, Dommasch M, Kanz KG, Wunderlich S, Knier B. Code Stroke Patient Referral by Emergency Medical Services During the Public COVID-19 Pandemic Lockdown. J Stroke Cerebrovasc Dis. 2020 Nov;29(11):105175. doi: 10.1016/j.jstrokecerebrovasdis.2020.105175. Epub 2020 Jul 21. PMID: 33066900; PMCID: PMC7373060.
- Asadi-Pooya AA, Farazdaghi M, Bazrafshan M. Impacts of the COVID-19 pandemic on Iranian patients with epilepsy. Acta Neurol Scand. 2020 Oct;142(4):392-395. doi: 10.1111/ane.13310. Epub 2020 Jul 22. PMID: 32632917; PMCID: PMC7362008.
- Kamdar HA, Senay B, Mainali S, Lee V, Gulati DK, Greene-Chandos D, Hinduja A, Strohm T. Clinician's Perception of Practice Changes for Stroke During the COVID-19 Pandemic. J Stroke Cerebrovasc Dis. 2020 Oct;29(10):105179. doi: 10.1016/j.jstrokecerebrovasdis.2020.105179. Epub 2020 Jul 22. PMID: 32912564; PMCID: PMC7375301.
- Riccio E, Pieroni M, Limoneglli G, Pisani A. Impact of COVID-19 pandemic on patients with Fabry disease: An Italian experience. Mol Genet Metab. 2020 Sep-Oct;131(1-2):124-125. doi: 10.1016/j.ymgme.2020.07.008. Epub 2020 Jul 28. PMID: 32741663; PMCID: PMC7386205.
- Adebayo PB, Jusabani A, Mukhtar M, Zehri AA. The changing trend of teleconsultations during COVID-19 era at a tertiary facility in Tanzania. Pan Afr Med J. 2020 Jul 28;35(Suppl 2):125. doi: 10.11604/pamj.supp.2020.35.2.24977. PMID: 33282080; PMCID: PMC7687499.
- Correa DJ, Labovitz DL, Milstein MJ, Monderer R, Haut SR. Folding a neuroscience center into streamlined COVID-19 response teams: Lessons in origami. Neurology. 2020 Sep 29;95(13):583-592. doi: 10.1212/WNL.0000000000010542. Epub 2020 Jul 30. PMID: 32732292.
- Dressler D, Adib Saberi F. Botulinum toxin therapy in the SARS-CoV-2 pandemic: patient perceptions from a German cohort. J Neural Transm (Vienna). 2020 Sep;127(9):1271-1274. doi: 10.1007/s00702-020-02235-6. Epub 2020 Jul 30. PMID: 32734554; PMCID: PMC7391233.
- La Corte E, Palandri G. Letter to the Editor: COVID-19 and the Neurosurgical Treatment of Idiopathic Normal Pressure Hydrocephalus: Shall We Continue to Postpone "Non-emergent" Surgical Procedures? World Neurosurg. 2020 Sep;141:578-579. doi: 10.1016/j.wneu.2020.06.242. Epub 2020 Jul 30. PMID: 32741739; PMCID: PMC7392589.
- Jasne AS, Chojecka P, Maran I, Mageid R, Eldokmak M, Zhang Q, Nystrom K, Vlieks K, Askenase M, Petersen N, Falcone GJ, Wira CR 3rd, Lleva P, Zeevi N, Narula R, Amin H, Navaratnam D, Loomis C, Hwang DY, Schindler J, Hebert R, Matouk C, Krumholz HM, Spudich S, Sheth KN, Sansing LH, Sharma R. Stroke Code Presentations, Interventions, and Outcomes Before and During the COVID-19 Pandemic. Stroke. 2020 Sep;51(9):2664-2673. doi: 10.1161/STR.0000000000000347. Epub 2020 Jul 31. PMID: 32755347; PMCID: PMC7446978.
- Giamello JD, Abram S, Bernardi S, Lauria G. The emergency department in the COVID-19 era. Who are we missing? Eur J Emerg Med. 2020 Aug;27(4):305-306. doi: 10.1097/MEJ.0000000000000718. PMID: 32345851; PMCID: PMC7202118.
- Granata T, Bisulli F, Arzimanoglou A, Rocamora R. Did the COVID-19 pandemic silence the needs of people with epilepsy? Epileptic Disord. 2020 Aug 1;22(4):439-442. doi: 10.1684/epd.2020.1175. PMID: 32759092; PMCID: PMC7537265.
- Oseran AS, Nash D, Kim C, Moisuk S, Lai PY, Pyhtila J, Sequist TD, Wasfy JH. Changes in hospital admissions for urgent conditions during COVID-19 pandemic. Am J Manag Care. 2020 Aug;26(8):327-328. doi: 10.37765/ajmc.2020.43837. PMID: 32835458.
- Jafarzadeh-Esfehani R, Mirzaei Fard M, Habibi Hatam-Ghale F, Rezaei Kalat A, Fathi A, Shariati M, Sadr-Nabavi A, Miri R, Bidkhori HR, Aelami MH. Telemedicine and Computer-Based Technologies during Coronavirus Disease 2019 Infection; A Chance to Educate and Diagnose. Arch Iran Med. 2020 Aug 1;23(8):561-563. doi: 10.34172/aim.2020.60. PMID: 32894969.
- Wahezi SE, Duarte RA, Yerra S, Thomas MA, Pujar B, Sehgal N, Argoff C, Manchikanti L, Gonzalez D, Jain R, Kim CH, Hossack M, Senthelal S, Jain A, Leo N, Shaparin N, Wong D, Wong A, Nguyen K, Singh JR, Grieco G, Patel A, Kinon MD, Kaye AD. Telemedicine During COVID-19 and Beyond: A Practical Guide and Best Practices Multidisciplinary Approach for the Orthopedic and Neurologic Pain Physical Examination. Pain Physician. 2020 Aug;23(4S):S205-S238. Erratum in: Pain Physician. 2020 Nov;23(6):647. PMID: 32942812.
- Katsanos AH, de Sa Boasquevisque D, Al-Qarni MA, Shawawrah M, McNicoll-Whiteman R, Gould L, Van Adel B, Sahlas DJ, Ng KKH, Perera K, Sharma M, Oczkowski W, Pikula A, Shoamanesh A, Catanese L. In-Hospital Delays for Acute Stroke Treatment Delivery During the COVID-19 Pandemic. Can J Neurol Sci. 2021 Jan;48(1):59-65. doi: 10.1017/cjn.2020.170. Epub 2020 Aug 3. PMID: 32741386; PMCID: PMC7533482.
- Studart-Neto A, Guedes BF, Tuma RLE, Camelo Filho AE, Kubota GT, Iepsen BD, Moreira GP, Rodrigues JC, Ferrari MMH, Carra RB, Spera RR, Oku MHM, Terrim S, Lopes CCB, Passos Neto CEB, Fiorentino MD, DE Souza JCC, Baima JPS, DA Silva TFF, Moreno CAM, Silva AMS, Heise CO, MendonÇa RH, Fortini I, Smid J, Adoni T, GonÇalves MRR, Pereira SLA, Pinto LF, Gomes HR, Zanoteli E, Brucki SMD, Conforto AB, Castro LHM, Nitrini R. Neurological consultations and diagnoses in a large, dedicated COVID-19 university hospital. Arq Neuropsiquiatr. 2020 Aug 3;78(8):494-500. doi: 10.1590/0004-282x20200089. PMID: 32756734.
- Sorbara M, Graviotto HG, Lage-Ruiz GM, Turizo-Rodriguez CM, Sotelo-López LA, Serra A, Gagliardi C, Heinemann G, Martinez P, Ces-Magliano F, Serrano CM. COVID-19 and the forgotten pandemic: follow-up of neurocognitive disorders during lockdown in Argentina. Neurologia. 2021 Jan-Feb;36(1):9-15. English, Spanish. doi: 10.1016/j.nrl.2020.07.015. Epub 2020 Aug 3. PMID: 32921515; PMCID: PMC7396892.
- Li L, Liu G, Xu W, Zhang Y, He M. Effects of Internet Hospital Consultations on Psychological Burdens and Disease Knowledge During the Early Outbreak of COVID-19 in China: Cross-Sectional Survey Study. J Med Internet Res. 2020 Aug 4;22(8):e19551. doi: 10.2196/19551. PMID: 32687061; PMCID: PMC7427983.
- Schwarz V, Mahfoud F, Lauder L, Reith W, Behnke S, Smola S, Rissland J, Pfuhl T, Scheller B, Böhm M, Ewen S. Decline of emergency admissions for cardiovascular and cerebrovascular events after the outbreak of COVID-19. Clin Res Cardiol. 2020 Dec;109(12):1500-1506. doi: 10.1007/s00392-020-01688-9. Epub 2020 Aug 4. PMID: 32749557; PMCID: PMC7399595.
- Seiffert M, Brunner FJ, Remmel M, Thomalla G, Marschall U, L'Hoest H, Acar L, Debus ES, Blankenberg S, Gerloff C, Behrendt CA. Temporal trends in the presentation of cardiovascular and cerebrovascular emergencies during the COVID-19 pandemic in Germany: an analysis of health insurance claims. Clin Res Cardiol. 2020 Dec;109(12):1540-1548. doi: 10.1007/s00392-020-01723-9. Epub 2020 Aug 4. PMID: 32749558; PMCID: PMC7402080.
- Gittins M, Ashton C, Holden N, Cross S, Meadipudi S, Kawafi K, Burger I, Rickard S, Vail A, Molloy J, Smith CJ. Environmental Factors and Hyperacute Stroke Care Activity During the COVID-19 Pandemic: An Interrupted Time-Series Analysis. J Stroke Cerebrovasc Dis. 2020 Nov;29(11):105229. doi: 10.1016/j.jstrokecerebrovasdis.2020.105229. Epub 2020 Aug 4. PMID: 32828638; PMCID: PMC7402099.
- Cummings C, Almallouhi E, Al Kasab S, Spiotta AM, Holmstedt CA. Blacks Are Less Likely to Present With Strokes During the COVID-19 Pandemic: Observations From the Buckle of the Stroke Belt. Stroke. 2020 Oct;51(10):3107-3111. doi: 10.1161/STROKEAHA.120.031121. Epub 2020 Aug 5. PMID: 32755454; PMCID: PMC7434003.
- Ding L, She Q, Chen F, Chen Z, Jiang M, Huang H, Li Y, Liao C. The Internet Hospital Plus Drug Delivery Platform for Health Management During the COVID-19 Pandemic: Observational Study. J Med Internet Res. 2020 Aug 6;22(8):e19678. doi: 10.2196/19678. PMID: 32716892; PMCID: PMC7419153.
- Nguyen-Huynh MN, Tang XN, Vinson DR, Flint AC, Alexander JG, Meighan M, Burnett M, Sidney S, Klingman JG. Acute Stroke Presentation, Care, and Outcomes in Community Hospitals in Northern California During the COVID-19 Pandemic. Stroke. 2020 Oct;51(10):2918-2924. doi: 10.1161/STROKEAHA.120.031099. Epub 2020 Aug 7. PMID: 32762619; PMCID: PMC7434008.
- Lai FH, Yan EW, Yu KK, Tsui WS, Chan DT, Yee BK. The Protective Impact of Telemedicine on Persons With Dementia and Their Caregivers During the COVID-19 Pandemic. Am J Geriatr Psychiatry. 2020 Nov;28(11):1175-1184. doi: 10.1016/j.jagp.2020.07.019. Epub 2020 Aug 8. PMID: 32873496; PMCID: PMC7413846.
- Power K, McCrea Z, White M, Breen A, Dunleavy B, O'Donoghue S, Jacquemard T, Lambert V, El-Naggar H, Delanty N, Doherty C, Fitzsimons M. The development of an epilepsy electronic patient portal: Facilitating both patient empowerment and remote clinician-patient interaction in a post-COVID-19 world. Epilepsia. 2020 Sep;61(9):1894-1905. doi: 10.1111/epi.16627. Epub 2020 Aug 10. PMID: 32668026; PMCID: PMC7404863.
- Moss BP, Mahajan KR, Bermel RA, Hellisz K, Hua LH, Hudec T, Husak S, McGinley MP, Ontaneda D, Wang Z, Weber M, Tagliani P, Cárdenas-Robledo S, Zabalza A, Arrambide G, Carbonell-Mirabent P, Rodríguez-Barranco M, Sastre-Garriga J, Tintore M, Montalban X, Douglas M, Ogbuokiri E, Aravidis B, Cohen JA, Mowry EM, Fitzgerald KC. Multiple sclerosis management during the COVID-19 pandemic. Mult Scler. 2020 Sep;26(10):1163-1171. doi: 10.1177/1352458520948231. Epub 2020 Aug 10. PMID: 32772807; PMCID: PMC7424611.
- Fargen KM, Leslie-Mazwi TM, Klucznik RP, Wolfe SQ, Brown P, Ansari SA, Dabus G, Spiotta AM, Mokin M, Hassan AE, Liebeskind D, Welch BG, Siddiqui AH, Hirsch JA. The professional and personal impact of the coronavirus pandemic on US neurointerventional practices: a nationwide survey. J Neurointerv Surg. 2020 Oct;12(10):927-931. doi: 10.1136/neurintsurg-2020-016513. Epub 2020 Aug 11. PMID: 32788389; PMCID: PMC7421723.
- von Wrede R, Moskau-Hartmann S, Baumgartner T, Helmstaedter C, Surges R. Counseling of people with epilepsy via telemedicine: Experiences at a German tertiary epilepsy center during the COVID-19 pandemic. Epilepsy Behav. 2020 Nov;112:107298. doi: 10.1016/j.yebeh.2020.107298. Epub 2020 Aug 12. PMID: 32801068; PMCID: PMC7422810.
- D'Haeseleer M, Eelen P, Sadeghi N, D'Hooghe MB, Van Schependom J, Nagels G. Feasibility of Real Time Internet-Based Teleconsultation in Patients With Multiple Sclerosis: Interventional Pilot Study. J Med Internet Res. 2020 Aug 13;22(8):e18178. doi: 10.2196/18178. PMID: 32447274; PMCID: PMC7453329.
- Gioia LC, Poppe AY, Laroche R, Dacier-Falque T, Sévigny I, Daneault N, Deschaintre Y, Jacquin G, Stapf C, Odier C. Streamlined Poststroke Treatment Order Sets During the SARS-CoV-2 Pandemic: Simplifying While Not Compromising Care. Stroke. 2020 Oct;51(10):3115-3118. doi: 10.1161/STROKEAHA.120.031008. Epub 2020 Aug 13. PMID: 32790493; PMCID: PMC7446994.
- Russi CS, Heaton HA, Demaerschalk BM. Emergency Medicine Telehealth for COVID-19: Minimize Front-Line Provider Exposure and Conserve Personal Protective Equipment. Mayo Clin Proc. 2020 Oct;95(10):2065-2068. doi: 10.1016/j.mayocp.2020.07.025. Epub 2020 Aug 13. PMID: 33012337; PMCID: PMC7425764.
- Erakovic J, Milikic D, Radulovic L, Perunicic S, Idrizovic Z, Roganovic M. Reorganization of multiple sclerosis health care system in Clinical Centre of Montenegro during the COVID-19 pandemic. eNeurologicalSci. 2020 Dec;21:100263. doi: 10.1016/j.ensci.2020.100263. Epub 2020 Aug 15. PMID: 32835119; PMCID: PMC7428433.
- Price SJ, Joannides A, Plaha P, Afshari FT, Albanese E, Barua NU, Chan HW, Critchley G, Flannery T, Fountain DM, Mathew RK, Piper RJ, Poon MT, Rajaraman C, Rominiyi O, Smith S, Solomou G, Solth A, Surash S, Wykes V, Watts C, Bulbeck H, Hutchinson P, Jenkinson MD; COVID-CNSMDT study group. Impact of COVID-19 pandemic on surgical neuro-oncology multi-disciplinary team decision making: a national survey (COVID-CNSMDT Study). BMJ Open. 2020 Aug 16;10(8):e040898. doi: 10.1136/bmjopen-2020-040898. PMID: 32801210; PMCID: PMC7430412.
- Lord AS, Lombardi N, Evans K, Deveaux D, Douglas E, Mansfield L, Zakin E, Jakubowska-Sadowska K, Grayson K, Omari M, Yaghi S, Humbert K, Sanger M, Kim S, Boffa M, Szuchumacher M, Jongeling A, Vazquez B, Berberi N, Kwon P, Locascio G, Chervinsky A, Frontera J, Zhou T, Kahn DE, Abou-Fayssal N. Keeping the team together: Transformation of an inpatient neurology service at an urban, multi-ethnic, safety net hospital in New York City during COVID-19. Clin Neurol Neurosurg. 2020 Oct;197:106156. doi: 10.1016/j.clineuro.2020.106156. Epub 2020 Aug 17. PMID: 32877768; PMCID: PMC7430288.
- Neece C, McIntyre LL, Fenning R. Examining the impact of COVID-19 in ethnically diverse families with young children with intellectual and developmental disabilities. J Intellect Disabil Res. 2020 Oct;64(10):739-749. doi: 10.1111/jir.12769. Epub 2020 Aug 18. PMID: 32808424; PMCID: PMC7461180.
- Sinha R, Anand V, Gupta J, Singh S, Gulati S. Infantile spasms and COVID-19: Challenges and solutions in resource-limited settings. Epilepsy Res. 2020 Nov;167:106441. doi: 10.1016/j.eplepsyres.2020.106441. Epub 2020 Aug 18. PMID: 32877881; PMCID: PMC7434386.
- Cheli M, Dinoto A, Olivo S, Tomaselli M, Stokelj D, Cominotto F, Brigo F, Manganotti P. SARS-CoV-2 pandemic and epilepsy: The impact on emergency department attendances for seizures. Seizure. 2020 Nov;82:23-26. doi: 10.1016/j.seizure.2020.08.008. Epub 2020 Aug 22. PMID: 32979601; PMCID: PMC7442554.
- Zaagsma M, Volkers KM, Swart EAK, Schippers AP, Van Hove G. The use of online support by people with intellectual disabilities living independently during COVID-19. J Intellect Disabil Res. 2020 Oct;64(10):750-756. doi: 10.1111/jir.12770. Epub 2020 Aug 24. PMID: 32830390; PMCID: PMC7461443.
- Al Kasab S, Almallouhi E, Alawieh A, Levitt MR, Jabbour P, Sweid A, Starke RM, Saini V, Wolfe SQ, Fargen KM, Arthur AS, Goyal N, Pandhi A, Fragata I, Maier I, Matouk C, Grossberg JA, Howard BM, Kan P, Hafeez M, Schirmer CM, Crowley RW, Joshi KC, Tjoumakaris SI, Chowdry S, Ares W, Ogilvy C, Gomez-Paz S, Rai AT, Mokin M, Guerrero W, Park MS, Mascitelli JR, Yoo A, Williamson R, Grande AW, Crosa RJ, Webb S, Psychogios MN, Ducruet AF, Holmstedt CA, Ringer AJ, Spiotta AM; STAR collaborators. International experience of mechanical thrombectomy during the COVID-19 pandemic: insights from STAR and ENRG. J Neurointerv Surg. 2020 Nov;12(11):1039-1044. doi: 10.1136/neurintsurg-2020-016671. Epub 2020 Aug 25. PMID: 32843359; PMCID: PMC7453763.
- Cabona C, Deleo F, Marinelli L, Audenino D, Arnaldi D, Rossi F, Di Giacomo R, Buffoni C, Rosa GJ, Didato G, Arboscello E, de Curtis M, Villani F. Epilepsy course during COVID-19 pandemic in three Italian epilepsy centers. Epilepsy Behav. 2020 Nov;112:107375. doi: 10.1016/j.yebeh.2020.107375. Epub 2020 Aug 25. PMID: 32858368; PMCID: PMC7445187.
- Hewitt KC, Rodgin S, Loring DW, Pritchard AE, Jacobson LA. Transitioning to telehealth neuropsychology service: Considerations across adult and pediatric care settings. Clin Neuropsychol. 2020 Oct-Nov;34(7-8):1335-1351. doi: 10.1080/13854046.2020.1811891. Epub 2020 Aug 26. PMID: 32842849.
- Hammers DB, Stolwyk R, Harder L, Cullum CM. A survey of international clinical teleneuropsychology service provision prior to and in the context of COVID-19. Clin Neuropsychol. 2020 Oct-Nov;34(7-8):1267-1283. doi: 10.1080/13854046.2020.1810323. Epub 2020 Aug 26. Erratum in: Clin Neuropsychol. 2020 Sep 12;:1. PMID: 32844714.
- Duncan C, Macleod AD. Video consultations in ordinary and extraordinary times. Pract Neurol. 2020 Oct;20(5):396-403. doi: 10.1136/practneurol-2020-002579. Epub 2020 Aug 29. PMID: 32862137.
- Cerulli Irelli E, Orlando B, Cocchi E, Morano A, Fattapposta F, Di Piero V, Toni D, Ciardi MR, Giallonardo AT, Fabbrini G, Berardelli A, Di Bonaventura C. The potential impact of enhanced hygienic measures during the COVID-19 outbreak on hospital-acquired infections: A pragmatic study in neurological units. J Neurol Sci. 2020 Nov 15;418:117111. doi: 10.1016/j.jns.2020.117111. Epub 2020 Aug 29. PMID: 32892033; PMCID: PMC7833504.
- Kuchenbuch M, D'Onofrio G, Wirrell E, Jiang Y, Dupont S, Grinspan ZM, Auvin S, Wilmshurst JM, Arzimanoglou A, Cross JH, Specchio N, Nabbout R. An accelerated shift in the use of remote systems in epilepsy due to the COVID-19 pandemic. Epilepsy Behav. 2020 Nov;112:107376. doi: 10.1016/j.yebeh.2020.107376. Epub 2020 Aug 31. PMID: 32882627; PMCID: PMC7457939.
- Prada V, Bellone E, Schenone A, Grandis M. The suspected SARS-Cov-2 infection in a Charcot-Marie-Tooth patient undergoing postsurgical rehabilitation: the value of telerehabilitation for evaluation and continuing treatment. Int J Rehabil Res. 2020 Sep;43(3):285-286. doi: 10.1097/MRR.0000000000000418. PMID: 32317558; PMCID: PMC7273849.
- Moss HE, Lai KE, Ko MW. Survey of Telehealth Adoption by Neuro-ophthalmologists During the COVID-19 Pandemic: Benefits, Barriers, and Utility. J Neuroophthalmol. 2020 Sep;40(3):346-355. doi: 10.1097/WNO.0000000000001051. PMID: 32639269; PMCID: PMC7382419.
- Elmaraghi S, Rao VK, Garland CB, Marcus BC, Mount DL. University of Wisconsin Guidelines for Treatment of Facial Trauma While Minimizing the Risk of COVID-19 Transmission. WMJ. 2020 Sep;119(3):202-204. PMID: 33091290.
- Silven AV, Petrus AHJ, Villalobos-Quesada M, Dirikgil E, Oerlemans CR, Landstra CP, Boosman H, van Os HJA, Blanker MH, Treskes RW, Bonten TN, Chavannes NH, Atsma DE, Teng YKO. Telemonitoring for Patients With COVID-19: Recommendations for Design and Implementation. J Med Internet Res. 2020 Sep 2;22(9):e20953. doi: 10.2196/20953. PMID: 32833660; PMCID: PMC7473766.
- Jayakumar N, Kennion O, Villabona AR, Paranathala M, Holliman D. Neurosurgical Referral Patterns During the Coronavirus Disease 2019 Pandemic: A United Kingdom Experience. World Neurosurg. 2020 Dec;144:e414-e420. doi: 10.1016/j.wneu.2020.08.162. Epub 2020 Sep 2. PMID: 32890845; PMCID: PMC7467101.
- Garnero M, Del Sette M, Assini A, Beronio A, Capello E, Cabona C, Reni L, Serrati C, Bandini F, Granata A, Pesce G, Mancardi GL, Uccelli A, Schenone A, Benedetti L. COVID-19-related and not related Guillain-Barré syndromes share the same management pitfalls during lock down: The experience of Liguria region in Italy. J Neurol Sci. 2020 Nov 15;418:117114. doi: 10.1016/j.jns.2020.117114. Epub 2020 Sep 2. PMID: 32947089; PMCID: PMC7462770.
- Dowlatshahi D, Stotts G, Bourgoin A, Gocan S, Dunn L, Powell J, Silver FL, Walker G, Yogendrakumar V, Fahed R, Blacquiere D, Shamy M. Decreased Stroke Presentation Rates at a Comprehensive Stroke Center during COVID-19. Can J Neurol Sci. 2021 Jan;48(1):118-121. doi: 10.1017/cjn.2020.193. Epub 2020 Sep 3. PMID: 32878659; PMCID: PMC7578633.
- Altschul DJ, Haranhalli N, Esenwa C, Unda SR, Garza Ramos R, Dardick J, Fernandez-Torres J, Toma A, Labovitz D, Cheng N, Lee SK, Brook A, Zampolin R. The Impact of COVID-19 on Emergent Large-Vessel Occlusion: Delayed Presentation Confirmed by ASPECTS. AJNR Am J Neuroradiol. 2020 Dec;41(12):2271-2273. doi: 10.3174/ajnr.A6800. Epub 2020 Sep 3. PMID: 32883669.
- Lubansu A, Assamadi M, Barrit S, Dembour V, Yao G, El Hadwe S, De Witte O. COVID-19 Impact on Neurosurgical Practice: Lockdown Attitude and Experience of a European Academic Center. World Neurosurg. 2020 Dec;144:e380-e388. doi: 10.1016/j.wneu.2020.08.168. Epub 2020 Sep 3. PMID: 32891850; PMCID: PMC7470722.
- Tejada Meza H, Lambea Gil Á, Sancho Saldaña A, Martínez-Zabaleta M, Garmendia Lopetegui E, López-Cancio Martínez E, Castañón Apilánez M, Herrera Isasi M, Marta Enguita J, Gómez-Vicente B, Arenillas JF, Arenaza Basterrechea N, Timiraos Fernández JJ, Sánchez Herrero J, Maciñeiras Montero JL, Castellanos Rodrigo M, Fernández-Coud D, Casado Menéndez I, Temprano Fernández MT, Freijo M, Luna A, Palacio Portilla EJ, Jiménez López Y, Rodríguez-Castro E, Rodríguez-Yáñez M, Tejada García J, Beltrán Rodríguez I, Julián-Villaverde F, Moreno García MP, Trejo Gabriel-Galán JM, Echavarría Iñiguez A, Pérez Lázaro C, Navarro Pérez MP, Marta Moreno J; NORDICTUS Investigators. Impact of COVID-19 outbreak in reperfusion therapies of acute ischaemic stroke in northwest Spain. Eur J Neurol. 2020 Dec;27(12):2491-2498. doi: 10.1111/ene.14467. Epub 2020 Sep 4. PMID: 32761981; PMCID: PMC7436392.
- Hagebusch P, Naujoks F, Rouchi H, Schindelin I, Schweigkofler U. Decline in emergency medical service missions during the COVID-19 pandemic: results from the fifth largest city in Germany. Intern Emerg Med. 2020 Nov;15(8):1609-1611. doi: 10.1007/s11739-020-02482-1. Epub 2020 Sep 6. PMID: 32893329; PMCID: PMC7474953.
- Collison M, Beiting KJ, Walker J, Huisingh-Scheetz M, Pisano J, Chia S, Marrs R, Landon E, Levine S, Gleason LJ. Three-Tiered COVID-19 Cohorting Strategy and Implications for Memory-Care. J Am Med Dir Assoc. 2020 Nov;21(11):1560-1562. doi: 10.1016/j.jamda.2020.09.001. Epub 2020 Sep 6. PMID: 33138937; PMCID: PMC7474901.
- Sanchez-Larsen A, Gonzalez-Villar E, Díaz-Maroto I, Layos-Romero A, Martínez-Martín Á, Alcahut-Rodriguez C, Grande-Martin A, Sopelana-Garay D. Influence of the COVID-19 outbreak in people with epilepsy: Analysis of a Spanish population (EPICOVID registry). Epilepsy Behav. 2020 Nov;112:107396. doi: 10.1016/j.yebeh.2020.107396. Epub 2020 Sep 7. PMID: 32911299; PMCID: PMC7476448.
- Giebel C, Hanna K, Cannon J, Eley R, Tetlow H, Gaughan A, Komuravelli A, Shenton J, Rogers C, Butchard S, Callaghan S, Limbert S, Rajagopal M, Ward K, Shaw L, Whittington R, Hughes M, Gabbay M. Decision-making for receiving paid home care for dementia in the time of COVID-19: a qualitative study. BMC Geriatr. 2020 Sep 9;20(1):333. doi: 10.1186/s12877-020-01719-0. PMID: 32900360; PMCID: PMC7478902.
- Van Hees S, Siewe Fodjo JN, Wijtvliet V, Van den Bergh R, Faria de Moura Villela E, da Silva CF, Weckhuysen S, Colebunders R. Access to healthcare and prevalence of anxiety and depression in persons with epilepsy during the COVID-19 pandemic: A multicountry online survey. Epilepsy Behav. 2020 Nov;112:107350. doi: 10.1016/j.yebeh.2020.107350. Epub 2020 Sep 10. PMID: 32920373; PMCID: PMC7481834.
- Ahmed ZM, Khalil MF, Kohail AM, Eldesouky IF, Elkady A, Shuaib A. The Prevalence and Predictors of Post-Stroke Depression and Anxiety During COVID-19 Pandemic. J Stroke Cerebrovasc Dis. 2020 Dec;29(12):105315. doi: 10.1016/j.jstrokecerebrovasdis.2020.105315. Epub 2020 Sep 10. PMID: 32958396; PMCID: PMC7834239.
- Wahlster S, Sharma M, Lewis AK, Patel PV, Hartog CS, Jannotta G, Blissitt P, Kross EK, Kassebaum NJ, Greer DM, Curtis JR, Creutzfeldt CJ. The Coronavirus Disease 2019 Pandemic's Effect on Critical Care Resources and Health-Care Providers: A Global Survey. Chest. 2021 Feb;159(2):619-633. doi: 10.1016/j.chest.2020.09.070. Epub 2020 Sep 11. PMID: 32926870; PMCID: PMC7484703.
- Mitra B, Mitchell RD, Cloud GC, Stub D, Nguyen M, Nanayakkara S, Miller JP, M O'Reilly G, Smit V, Cameron PA. Presentations of stroke and acute myocardial infarction in the first 28 days following the introduction of State of Emergency restrictions for COVID-19. Emerg Med Australas. 2020 Dec;32(6):1040-1045. doi: 10.1111/1742-6723.13621. Epub 2020 Sep 12. PMID: 32833297; PMCID: PMC7461453.
- Casares M, Wombles C, Skinner HJ, Westerveld M, Gireesh ED. Telehealth perceptions in patients with epilepsy and providers during the COVID-19 pandemic. Epilepsy Behav. 2020 Nov;112:107394. doi: 10.1016/j.yebeh.2020.107394. Epub 2020 Sep 12. PMID: 32932153.
- Monin JK, Ali T, Syed S, Piechota A, Lepore M, Mourgues C, Gaugler JE, Marottoli R, David D. Family Communication in Long-Term Care During a Pandemic: Lessons for Enhancing Emotional Experiences. Am J Geriatr Psychiatry. 2020 Dec;28(12):1299-1307. doi: 10.1016/j.jagp.2020.09.008. Epub 2020 Sep 12. PMID: 33004262; PMCID: PMC7486818.
- Alexopoulos P, Novotni A, Novotni G, Vorvolakos T, Vratsista A, Konsta A, Kaprinis S, Konstantinou A, Bonotis K, Katirtzoglou E, Siarkos K, Bekri ES, Kokkoris I, Como A, Gournellis R, Stoyanov DS, Politis A. Old age mental health services in Southern Balkans: Features, geospatial distribution, current needs, and future perspectives. Eur Psychiatry. 2020 Sep 14;63(1):e88. doi: 10.1192/j.eurpsy.2020.85. PMID: 32921324; PMCID: PMC7576530.
- Krieger SC. A neurologist in the COVID unit: Reflections on a redeployment. Neurology. 2020 Nov 10;95(19):877-880. doi: 10.1212/WNL.0000000000010850. Epub 2020 Sep 14. PMID: 32928972.
- Drunat O, Roche J, Kohler S, Julien V, Pascal S, Lenoir H, Soto-Martin M, Lepetit A, Volpe-Gillot L, Leclercq V, Romdhani M, Koskas P, Lebert F. What the COVID-19 pandemic entails for the management of patients with behavioral and psychological symptoms of dementia: experience in France. Int Psychogeriatr. 2020 Nov;32(11):1361-1364. doi: 10.1017/S1041610220003567. Epub 2020 Sep 15. PMID: 32930087; PMCID: PMC7573453.
- García-Moncó JC, Cabrera-Muras A, Collía-Fernández A, Erburu-Iriarte M, Rodrigo-Armenteros P, Oyarzun-Irazu I, Martínez-Condor D, Bilbao-González A, Carmona-Abellán M, Caballero-Romero I, Gómez-Beldarrain M. Neurological reasons for consultation and hospitalization during the COVID-19 pandemic. Neurol Sci. 2020 Nov;41(11):3031-3038. doi: 10.1007/s10072-020-04714-w. Epub 2020 Sep 15. PMID: 32935157; PMCID: PMC7491981.
- Ghoreishi A, Arsang-Jang S, Sabaa-Ayoun Z, Yassi N, Sylaja PN, Akbari Y, Divani AA, Biller J, Phan T, Steinwender S, Silver B, Zand R, Basri HB, Iqbal OM, Ranta A, Ruland S, Macri E, Ma H, Nguyen TN, Abootalebi S, Gupta A, Alet M, Lattanzi S, Desai M, Gagliardi RJ, Girotra T, Inoue M, Yoshimoto T, Isaac CF, Mayer SA, Morovatdar N, Nilanont Y, Nobleza COS, Saber H, Kamenova S, Kondybayeva A, Krupinski J, Siegler JE, Stranges S, Torbey MT, Yorio D, Zurrú MC, Rubinos CA, Shahripour RB, Borhani-Haghighi A, Napoli MD, Azarpazhooh MR. Stroke Care Trends During COVID-19 Pandemic in Zanjan Province, Iran. From the CASCADE Initiative: Statistical Analysis Plan and Preliminary Results. J Stroke Cerebrovasc Dis. 2020 Dec;29(12):105321. doi: 10.1016/j.jstrokecerebrovasdis.2020.105321. Epub 2020 Sep 16. PMID: 33069086; PMCID: PMC7494258.
- Low PH, Mangat MS, Liew DNS, Wong ASH. Neurosurgical Services in the Northern Zone of Sarawak in Malaysia: The Way Forward Amid the COVID-19 Pandemic. World Neurosurg. 2020 Dec;144:e710-e713. doi: 10.1016/j.wneu.2020.09.045. Epub 2020 Sep 17. PMID: 32949798; PMCID: PMC7494498.
- Bajunaid K, Alatar A, Alqurashi A, Alkutbi M, Alzahrani AH, Sabbagh AJ, Alobaid A, Barnawi A, Alferayan AA, Alkhani AM, Bin Salamah A, Sheikh BY, Alotaibi FE, Alabbas F, Farrash F, Al-Jehani HM, Alhabib H, Alnaami I, Altweijri I, Khoja I, Taha M, Alzahrani M, Bafaquh MS, Binmahfoodh M, Algahtany MA, Al-Rashed S, Raza SM, Elwatidy S, Alomar SA, Al-Issawi W, Khormi YH, Ammar A, Al-Habib A, Baeesa SS, Ajlan A. The longitudinal impact of COVID-19 pandemic on neurosurgical practice. Clin Neurol Neurosurg. 2020 Nov;198:106237. doi: 10.1016/j.clineuro.2020.106237. Epub 2020 Sep 17. PMID: 33002677; PMCID: PMC7497779.
- Jillella DV, Janocko NJ, Nahab F, Benameur K, Greene JG, Wright WL, Obideen M, Rangaraju S. Ischemic stroke in COVID-19: An urgent need for early identification and management. PLoS One. 2020 Sep 18;15(9):e0239443. doi: 10.1371/journal.pone.0239443. PMID: 32946512; PMCID: PMC7500690.
- Leung WCY, Lau EHY, Kwan P, Chang RS. Impact of COVID-19 on seizure-related emergency attendances and hospital admissions - A territory-wide observational study. Epilepsy Behav. 2021 Feb;115:107497. doi: 10.1016/j.yebeh.2020.107497. Epub 2020 Sep 21. PMID: 33317939; PMCID: PMC7505596.
- Williams R, Jenkins DA, Ashcroft DM, Brown B, Campbell S, Carr MJ, Cheraghi-Sohi S, Kapur N, Thomas O, Webb RT, Peek N. Diagnosis of physical and mental health conditions in primary care during the COVID-19 pandemic: a retrospective cohort study. Lancet Public Health. 2020 Oct;5(10):e543-e550. doi: 10.1016/S2468-2667(20)30201-2. Epub 2020 Sep 23. PMID: 32979305; PMCID: PMC7511209.
- Taddei M, Bulgheroni S. Facing the real time challenges of the COVID-19 emergency for child neuropsychology service in Milan. Res Dev Disabil. 2020 Dec;107:103786. doi: 10.1016/j.ridd.2020.103786. Epub 2020 Sep 23. PMID: 33007533; PMCID: PMC7510541.
- Al-Hashel JY, Ismail II. Impact of coronavirus disease 2019 (COVID-19) pandemic on patients with migraine: a web-based survey study. J Headache Pain. 2020 Sep 24;21(1):115. doi: 10.1186/s10194-020-01183-6. PMID: 32972360; PMCID: PMC7513457.
- Sun S, Folarin AA, Ranjan Y, Rashid Z, Conde P, Stewart C, Cummins N, Matcham F, Dalla Costa G, Simblett S, Leocani L, Lamers F, Sørensen PS, Buron M, Zabalza A, Guerrero Pérez AI, Penninx BW, Siddi S, Haro JM, Myin-Germeys I, Rintala A, Wykes T, Narayan VA, Comi G, Hotopf M, Dobson RJ; RADAR-CNS Consortium. Using Smartphones and Wearable Devices to Monitor Behavioral Changes During COVID-19. J Med Internet Res. 2020 Sep 25;22(9):e19992. doi: 10.2196/19992. PMID: 32877352; PMCID: PMC7527031.
- Elmonem MA, Belanger-Quintana A, Bordugo A, Boruah R, Cortès-Saladelafont E, Endrakanti M, Giraldo P, Grünert SC, Gupta N, Kabra M, Knerr I, Krämer J, Kuster A, Levtchenko E, Ngu LH, Rovira-Remisa MM, Sass JO, Sykut-Cegielska J, Tummolo A, van den Heuvel LP. The impact of COVID-19 pandemic on the diagnosis and management of inborn errors of metabolism: A global perspective. Mol Genet Metab. 2020 Nov;131(3):285-288. doi: 10.1016/j.ymgme.2020.09.004. Epub 2020 Sep 25. PMID: 33004274; PMCID: PMC7518833.
- Wang J, Chaudhry SA, Tahsili-Fahadan P, Altaweel LR, Bashir S, Bahiru Z, Fang Y, Qureshi AI. The impact of COVID-19 on acute ischemic stroke admissions: Analysis from a community-based tertiary care center. J Stroke Cerebrovasc Dis. 2020 Dec;29(12):105344. doi: 10.1016/j.jstrokecerebrovasdis.2020.105344. Epub 2020 Sep 25. PMID: 33049464; PMCID: PMC7518171.
- Saleem T, Sheikh N, Abbasi MH, Javed I, Khawar MB. COVID-19 containment and its unrestrained impact on epilepsy management in resource-limited areas of Pakistan. Epilepsy Behav. 2020 Nov;112:107476. doi: 10.1016/j.yebeh.2020.107476. Epub 2020 Sep 25. PMID: 33181895; PMCID: PMC7518832.
- Quinn L, Macpherson C, Long K, Shah H. Promoting Physical Activity via Telehealth in People With Parkinson Disease: The Path Forward After the COVID-19 Pandemic? Phys Ther. 2020 Sep 28;100(10):1730-1736. doi: 10.1093/ptj/pzaa128. PMID: 32734298; PMCID: PMC7454884.
- Wang X, Ouyang M, Carcel C, Chen C, Sun L, Yang J, Zhang Y, Chen G, You S, Cao Y, Ma L, Hu X, Sui Y, Anderson C, Song L, Wang Y, Wang D. Impact of COVID-2019 on stroke services in China: survey from the Chinese Stroke Association. Stroke Vasc Neurol. 2020 Dec;5(4):323-330. doi: 10.1136/svn-2020-000514. Epub 2020 Sep 28. PMID: 32989012; PMCID: PMC7523176.
- Ohara N, Imamura H, Adachi H, Hara Y, Hosoda K, Kimura H, Kuwayama K, Mizowaki T, Motooka Y, Nakashima K, Shinoda N, Takamoto T, Ueno Y, Yamaura I, Yanagihara C, Yoshida Y, Kawamoto M, Sakai N. Stroke Systems of Care During the COVID-19 Epidemic in Kobe City. J Stroke Cerebrovasc Dis. 2020 Dec;29(12):105343. doi: 10.1016/j.jstrokecerebrovasdis.2020.105343. Epub 2020 Sep 30. PMID: 33039766; PMCID: PMC7526598.
- Turan TN, Meschia JF, Chimowitz MI, Roldan A, LeMatty T, Luke S, Breathitt L, Eiland R, Foley J, Brott TG. Mitigating the effects of COVID-19 pandemic on controlling vascular risk factors among participants in a carotid stenosis trial. J Stroke Cerebrovasc Dis. 2020 Dec;29(12):105362. doi: 10.1016/j.jstrokecerebrovasdis.2020.105362. Epub 2020 Sep 30. PMID: 33071206; PMCID: PMC7524666.
- Paliwal PR, Tan BYQ, Leow AST, Sibi S, Chor DWP, Chin AXY, Yau YW, Cross GB, Wong LYH, Chia MLJ, Quak Z, Chua CYK, Tang DKK, Zune ET, Hung J, Goh Y, Jing M, Gopinathan A, Yang C, Ahmad A, Khoo DXL, Lee CM, Seet RCS, Sharma VK, Teoh HL, Yeo LLL, Chan BPL. Impact of the COVID-19 pandemic on hyperacute stroke treatment: experience from a comprehensive stroke centre in Singapore. J Thromb Thrombolysis. 2020 Oct;50(3):596-603. doi: 10.1007/s11239-020-02225-1. PMID: 32661758; PMCID: PMC7358288.
- Klingner CC, Brodoehl S, Wagner F, Berrouschot J, Günther A, Witte OW, Klingner CM. Effektivität, Effizienz und Sicherheit der Schlaganfall-Telemedizin in Zeiten der Corona-Pandemie : Der „Fall“ Thüringen [Effectiveness, efficiency and safety of stroke telemedicine in times of the coronavirus pandemic : The "case" Thuringia]. Nervenarzt. 2020 Oct;91(10):946-951. German. doi: 10.1007/s00115-020-00970-5. PMID: 32747988; PMCID: PMC7397963.
- Avelino PR, Menezes KKP, Nascimento LR, Tenório RA, Cândido GN, Christovão IS, Teixeira-Salmela LF. Validation of the Telephone-Based Application of the ABILHAND for Assessment of Manual Ability After Stroke. J Neurol Phys Ther. 2020 Oct;44(4):256-260. doi: 10.1097/NPT.0000000000000326. PMID: 32815891.
- Hassan A, Mari Z, Gatto EM, Cardozo A, Youn J, Okubadejo N, Bajwa JA, Shalash A, Fujioka S, Aldaajani Z, Cubo E; International Telemedicine Study Group. Global Survey on Telemedicine Utilization for Movement Disorders During the COVID-19 Pandemic. Mov Disord. 2020 Oct;35(10):1701-1711. doi: 10.1002/mds.28284. PMID: 32833273; PMCID: PMC7461376.
- Lee KD, Lee SB, Lim JK, Kang YM, Kim IB, Moon HJ, Lee WJ. Providing essential clinical care for non-COVID-19 patients in a Seoul metropolitan acute care hospital amidst ongoing treatment of COVID-19 patients. J Hosp Infect. 2020 Dec;106(4):673-677. doi: 10.1016/j.jhin.2020.09.031. Epub 2020 Oct 1. PMID: 33011308; PMCID: PMC7528870.
- Ojetti V, Covino M, Brigida M, Petruzziello C, Saviano A, Migneco A, Candelli M, Franceschi F. Non-COVID Diseases during the Pandemic: Where Have All Other Emergencies Gone? Medicina (Kaunas). 2020 Oct 1;56(10):512. doi: 10.3390/medicina56100512. PMID: 33019514; PMCID: PMC7599851.
- Krysl D, Beniczky S, Franceschetti S, Arzimanoglou A. The COVID-19 outbreak and approaches to performing EEG in Europe. Epileptic Disord. 2020 Oct 1;22(5):548-554. doi: 10.1684/epd.2020.1208. PMID: 33095171; PMCID: PMC7753285.
- Altman RL, Anstett T, Simpson JR, Del Pino-Jones A, Lin CT, Pell J. Ambulatory Clinician's Guide to Inpatient Service: An Innovative Rapid Onboarding Strategy for the COVID-19 Pandemic. Appl Clin Inform. 2020 Oct;11(5):802-806. doi: 10.1055/s-0040-1719042. Epub 2020 Dec 2. PMID: 33264802; PMCID: PMC7710441.
- Kumar A. Experience of video consultation during the COVID-19 pandemic in elderly population for Parkinson's disease and movement disorders. Postgrad Med J. 2021 Feb;97(1144):117-118. doi: 10.1136/postgradmedj-2020-138846. Epub 2020 Oct 2. PMID: 33008959; PMCID: PMC7548126.
- Semprino M, Fasulo L, Fortini S, Martorell Molina CI, González L, Ramos PA, Martínez C, Caraballo R. Telemedicine, drug-resistant epilepsy, and ketogenic dietary therapies: A patient survey of a pediatric remote-care program during the COVID-19 pandemic. Epilepsy Behav. 2020 Nov;112:107493. doi: 10.1016/j.yebeh.2020.107493. Epub 2020 Oct 3. PMID: 33181913; PMCID: PMC7532773.
- Bechstein M, Elsheikh S, Wodarg F, Taschner CA, Hanning U, Buhk JH, McDonough R, Goebell E, Fiehler J, Bester M. Interhospital teleproctoring of endovascular intracranial aneurysm treatment using a dedicated live-streaming technology: first experiences during the COVID-19 pandemic. BMJ Case Rep. 2020 Oct 4;13(10):e016722. doi: 10.1136/bcr-2020-016722. PMID: 33012707; PMCID: PMC7536784.
- Kabir ZN, Boström AM, Konradsen H. In Conversation with a Frontline Worker in a Care Home in Sweden during the COVID-19 Pandemic. J Cross Cult Gerontol. 2020 Dec;35(4):493-500. doi: 10.1007/s10823-020-09415-7. Epub 2020 Oct 5. PMID: 33015728; PMCID: PMC7533166.
- Livingston G, Rostamipour H, Gallagher P, Kalafatis C, Shastri A, Huzzey L, Liu K, Sommerlad A, Marston L. Prevalence, management, and outcomes of SARS-CoV-2 infections in older people and those with dementia in mental health wards in London, UK: a retrospective observational study. Lancet Psychiatry. 2020 Dec;7(12):1054-1063. doi: 10.1016/S2215-0366(20)30434-X. Epub 2020 Oct 5. PMID: 33031760; PMCID: PMC7535621.
- Davico C, Marcotulli D, Lux C, Calderoni D, Terrinoni A, Di Santo F, Ricci F, Vittorini R, Amianto F, Urbino A, Ferrara M, Vitiello B. Where have the children with epilepsy gone? An observational study of seizure-related accesses to emergency department at the time of COVID-19. Seizure. 2020 Dec;83:38-40. doi: 10.1016/j.seizure.2020.09.025. Epub 2020 Oct 5. PMID: 33080483; PMCID: PMC7534601.
- Nkodo JA, Camus V, Fougère B. Ethical Issues in the Management of Patients With Behavioral and Psychological Symptoms of Dementia During COVID-19 Containment: Examples From Institutions in France. Am J Geriatr Psychiatry. 2020 Dec;28(12):1332-1333. doi: 10.1016/j.jagp.2020.10.001. Epub 2020 Oct 6. PMID: 33077342; PMCID: PMC7537620.
- Dhiman S, Sahu PK, Reed WR, Ganesh GS, Goyal RK, Jain S. Impact of COVID-19 outbreak on mental health and perceived strain among caregivers tending children with special needs. Res Dev Disabil. 2020 Dec;107:103790. doi: 10.1016/j.ridd.2020.103790. Epub 2020 Oct 6. PMID: 33091712; PMCID: PMC7538124.
- Mag Uidhir F, Bathula R, Sivagnanaratnam A, Abdul-Saheb M, Devine J, Cohen DL. Impact of COVID-19 on Stroke Caseload in a Major Hyperacute Stroke Unit. J Stroke Cerebrovasc Dis. 2020 Dec;29(12):105383. doi: 10.1016/j.jstrokecerebrovasdis.2020.105383. Epub 2020 Oct 6. PMID: 33099122; PMCID: PMC7538088.
- Willems LM, Balcik Y, Noda AH, Siebenbrodt K, Leimeister S, McCoy J, Kienitz R, Kiyose M, Reinecke R, Schäfer JH, Zöllner JP, Bauer S, Rosenow F, Strzelczyk A. SARS-CoV-2-related rapid reorganization of an epilepsy outpatient clinic from personal appointments to telemedicine services: A German single-center experience. Epilepsy Behav. 2020 Nov;112:107483. doi: 10.1016/j.yebeh.2020.107483. Epub 2020 Oct 6. PMID: 33181898; PMCID: PMC7537633.
- Lun R, Walker G, Daham Z, Ramsay T, Portela de Oliveira E, Kassab M, Fahed R, Quateen A, Lesiuk H, P Dos Santos M, Drake B. Transition to virtual appointments for interventional neuroradiology due to the COVID-19 pandemic: a survey of satisfaction. J Neurointerv Surg. 2020 Dec;12(12):1153-1156. doi: 10.1136/neurintsurg-2020-016805. Epub 2020 Oct 7. PMID: 33028673.
- Howley F, O'Doherty L, McEniff N, O'Riordan R. Late presentation of 'Lemierre's syndrome': how a delay in seeking healthcare and reduced access to routine services resulted in widely disseminated *Fusobacterium necrophorum* infection during the global COVID-19 pandemic. BMJ Case Rep. 2020 Oct 10;13(10):e239269. doi: 10.1136/bcr-2020-239269. PMID: 33040042; PMCID: PMC7549461.
- Abbasi J. Portable MRI-Coming to the ICU? JAMA. 2020 Oct 13;324(14):1386. doi: 10.1001/jama.2020.19612. PMID: 33048138.
- Carpinelli Mazzi M, Iavarone A, Musella C, De Luca M, de Vita D, Branciforte S, Coppola A, Scarpa R, Raimondo S, Sorrentino S, Lualdi F, Postiglione A. Time of isolation, education and gender influence the psychological outcome during COVID-19 lockdown in caregivers of patients with dementia. Eur Geriatr Med. 2020 Dec;11(6):1095-1098. doi: 10.1007/s41999-020-00413-z. Epub 2020 Oct 14. PMID: 33052535; PMCID: PMC7556578.
- Moo LR. Home Video Visits: Two-Dimensional View of the Geriatric 5 M's. J Am Geriatr Soc. 2020 Nov;68(11):2425-2427. doi: 10.1111/jgs.16843. Epub 2020 Oct 15. PMID: 32930388.
- Zhao J, Wang Y, Fisher M, Liu R. Slower recovery of outpatient clinics than inpatient services for stroke and other neurological diseases after COVID-19 Pandemic. CNS Neurosci Ther. 2020 Dec;26(12):1322-1326. doi: 10.1111/cns.13459. Epub 2020 Oct 15. PMID: 33058536; PMCID: PMC7675482.
- Loman M, Vogt E, Miller L, Landsman R, Duong P, Kasten J, DeFrancisco D, Koop J, Heffelfinger A. "How to" operate a pediatric neuropsychology practice during the COVID-19 pandemic: Real tips from one practice's experience. Child Neuropsychol. 2021 Feb;27(2):251-279. doi: 10.1080/09297049.2020.1830962. Epub 2020 Oct 16. PMID: 33059534.
- Tailby C, Collins AJ, Vaughan DN, Abbott DF, O'Shea M, Helmstaedter C, Jackson GD. Teleneuropsychology in the time of COVID-19: The experience of The Australian Epilepsy Project. Seizure. 2020 Dec;83:89-97. doi: 10.1016/j.seizure.2020.10.005. Epub 2020 Oct 16. PMID: 33120327; PMCID: PMC7561524.
- Murdin L, Saman Y, Rea P. The remote neuro-otology assessment - managing dizziness in the coronavirus disease 2019 era. J Laryngol Otol. 2020 Dec;134(12):1120-1122. doi: 10.1017/S0022215120002273. Epub 2020 Oct 21. PMID: 33081869; PMCID: PMC7642955.
- Alnajashi H, Jabbad R. Behavioral practices of patients with multiple sclerosis during Covid-19 pandemic. PLoS One. 2020 Oct 22;15(10):e0241103. doi: 10.1371/journal.pone.0241103. PMID: 33091088; PMCID: PMC7580932.
- Palladino F, Merolla E, Solimeno M, de Leva MF, Lenta S, Di Mita O, Bonadies A, Striano P, Tipo V, Varone A. Is Covid-19 lockdown related to an increase of accesses for seizures in the emergency department? An observational analysis of a paediatric cohort in the Southern Italy. Neurol Sci. 2020 Dec;41(12):3475-3483. doi: 10.1007/s10072-020-04824-5. Epub 2020 Oct 23. PMID: 33095368; PMCID: PMC7582024.
- D'Anna L, Sheikh A, Bathula R, Elmamoun S, Oppong A, Singh R, Redwood R, Janssen J, Banerjee S, Vasileiadis E. Decreasing referrals to transient ischaemic attack clinics during the COVID-19 outbreak: results from a multicentre cross-sectional survey. BMJ Open. 2020 Oct 23;10(10):e041514. doi: 10.1136/bmjopen-2020-041514. PMID: 33099501; PMCID: PMC7590351.
- Montagnon R, Rouffilange L, Agard G, Benner P, Cazes N, Renard A. Impact of the COVID-19 Pandemic on Emergency Department Use: Focus on Patients Requiring Urgent Revascularization. J Emerg Med. 2021 Feb;60(2):229-236. doi: 10.1016/j.jemermed.2020.09.042. Epub 2020 Oct 28. PMID: 33129611; PMCID: PMC7598350.
- Wong SYS, Zhang D, Sit RWS, Yip BHK, Chung RY, Wong CKM, Chan DCC, Sun W, Kwok KO, Mercer SW. Impact of COVID-19 on loneliness, mental health, and health service utilisation: a prospective cohort study of older adults with multimorbidity in primary care. Br J Gen Pract. 2020 Oct 29;70(700):e817-e824. doi: 10.3399/bjgp20X713021. PMID: 32988955; PMCID: PMC7523921.
- Kristoffersen ES, Faiz KW, Sandset EC, Storstein AM, Stefansen S, Winsvold BS, Hansen JM. Hospital-based headache care during the Covid-19 pandemic in Denmark and Norway. J Headache Pain. 2020 Oct 29;21(1):128. doi: 10.1186/s10194-020-01195-2. Erratum in: J Headache Pain. 2020 Nov 16;21(1):132. PMID: 33121445; PMCID: PMC7594963.
- Henriksen J, Kolognizak T, Houghton T, Cherne S, Zhen D, Cimino PJ, Latimer CS, Scherpelz KP, Yoda RA, Alpers CE, Chhieng DF, Keene CD, Gonzalez-Cuyar LF. Rapid Validation of Telepathology by an Academic Neuropathology Practice During the COVID-19 Pandemic. Arch Pathol Lab Med. 2020 Nov 1;144(11):1311-1320. doi: 10.5858/arpa.2020-0372-SA. PMID: 32551815.
- Tenforde AS, Borgstrom H, Polich G, Steere H, Davis IS, Cotton K, O'Donnell M, Silver JK. Outpatient Physical, Occupational, and Speech Therapy Synchronous Telemedicine: A Survey Study of Patient Satisfaction with Virtual Visits During the COVID-19 Pandemic. Am J Phys Med Rehabil. 2020 Nov;99(11):977-981. doi: 10.1097/PHM.0000000000001571. PMID: 32804713; PMCID: PMC7526401.
- Esper GJ, Sweeney RL, Winchell E, Duffell JM, Kier SC, Lukens HW, Krupinski EA. Rapid Systemwide Implementation of Outpatient Telehealth in Response to the COVID-19 Pandemic. J Healthc Manag. 2020 Nov-Dec;65(6):443-452. doi: 10.1097/JHM-D-20-00131. PMID: 33074968; PMCID: PMC7671820.
- Klein SJ, Bellmann R, Dejaco H, Eschertzhuber S, Fries D, Furtwängler W, Gasteiger L, Hasibeder W, Helbok R, Hochhold C, Hofer S, Kirchmair L, Krismer C, Ladner E, Lehner GF, Mathis S, Mayr A, Mittermayr M, Peer A, Preuß Hernández C, Reitter B, Ströhle M, Swoboda M, Thomé C, Joannidis M. Structured ICU resource management in a pandemic is associated with favorable outcome in critically ill COVID‑19 patients. Wien Klin Wochenschr. 2020 Nov;132(21-22):653-663. doi: 10.1007/s00508-020-01764-0. Epub 2020 Nov 10. PMID: 33170333; PMCID: PMC7653454.
- Mehta J, Yates T, Smith P, Henderson D, Winteringham G, Burns A. Rapid implementation of Microsoft Teams in response to COVID-19: one acute healthcare organisation's experience. BMJ Health Care Inform. 2020 Nov;27(3):e100209. doi: 10.1136/bmjhci-2020-100209. PMID: 33177050; PMCID: PMC7661347.
- Ballesta-Martínez S, Navarro-Pérez MP, Espinosa-Rueda J, Marín-Gracia M, García-Noain JA, Muñoz-Farjas E. Care of neurology patients in a hospital emergency department during the lockdown period for COVID-19: a comparative analysis. Emergencias. 2020 Nov;32(6):442-444. Spanish, English. PMID: 33275371.
- Alsaffar H, Almamari W, Al Futaisi A. Telemedicine in the Era of COVID-19 and Beyond: A new horizon. Sultan Qaboos Univ Med J. 2020 Nov;20(4):e277-e279. doi: 10.18295/squmj.2020.20.04.001. Epub 2020 Dec 21. PMID: 33414930; PMCID: PMC7757927.
- Naveen R, Sundaram TG, Agarwal V, Gupta L. Teleconsultation experience with the idiopathic inflammatory myopathies: a prospective observational cohort study during the COVID-19 pandemic. Rheumatol Int. 2021 Jan;41(1):67-76. doi: 10.1007/s00296-020-04737-8. Epub 2020 Nov 4. PMID: 33150493; PMCID: PMC7640991.
- Subotic A, Pricop DF, Josephson CB, Patten SB, Smith EE, Roach P; Calgary Comprehensive Epilepsy Program Collaborators. Examining the impacts of the COVID-19 pandemic on the well-being and virtual care of patients with epilepsy. Epilepsy Behav. 2020 Dec;113:107599. doi: 10.1016/j.yebeh.2020.107599. Epub 2020 Nov 4. PMID: 33238236.
- Balestrini S, Koepp MJ, Gandhi S, Rickman HM, Shin GY, Houlihan CF, Anders-Cannon J, Silvennoinen K, Xiao F, Zagaglia S, Hudgell K, Ziomek M, Haimes P, Sampson A, Parker A, Helen Cross J, Pardington R, Nastouli E, Swanton C; Crick COVID Consortium (CCC), Sander JW, Sisodiya SM; ChAlfont keepS vulnerAble People safe (ASAP) Consortium:. Clinical outcomes of COVID-19 in long-term care facilities for people with epilepsy. Epilepsy Behav. 2021 Feb;115:107602. doi: 10.1016/j.yebeh.2020.107602. Epub 2020 Nov 5. PMID: 33279440; PMCID: PMC7643621.
- Provenzi L, Grumi S, Gardani A, Aramini V, Dargenio E, Naboni C, Vacchini V, Borgatti R; Engaging with Families through On-line Rehabilitation for Children during the Emergency (EnFORCE) Group. Italian parents welcomed a telehealth family-centred rehabilitation programme for children with disability during COVID-19 lockdown. Acta Paediatr. 2021 Jan;110(1):194-196. doi: 10.1111/apa.15636. Epub 2020 Nov 6. PMID: 33098116.
- Watson N, Kurudzhu H, Green A, Summers D, Smith C, Pal S. Application of telehealth for comprehensive Creutzfeldt-Jakob disease surveillance in the United Kingdom. J Neurol Sci. 2021 Jan 15;420:117221. doi: 10.1016/j.jns.2020.117221. Epub 2020 Nov 7. PMID: 33223148.
- Grumi S, Provenzi L, Gardani A, Aramini V, Dargenio E, Naboni C, Vacchini V, Borgatti R; Engaging with Families through On-line Rehabilitation for Children during the Emergency (EnFORCE) Group. Rehabilitation services lockdown during the COVID-19 emergency: the mental health response of caregivers of children with neurodevelopmental disabilities. Disabil Rehabil. 2021 Jan;43(1):27-32. doi: 10.1080/09638288.2020.1842520. Epub 2020 Nov 10. PMID: 33167738.
- Sharawat IK, Panda PK. Caregiver Satisfaction and Effectiveness of Teleconsultation in Children and Adolescents With Migraine During the Ongoing COVID-19 Pandemic. J Child Neurol. 2021 Mar;36(4):296-303. doi: 10.1177/0883073820968653. Epub 2020 Nov 10. PMID: 33170754.
- Weiss EF, Malik R, Santos T, Ceide M, Cohen J, Verghese J, Zwerling JL. Telehealth for the cognitively impaired older adult and their caregivers: lessons from a coordinated approach. Neurodegener Dis Manag. 2021 Feb;11(1):83-89. doi: 10.2217/nmt-2020-0041. Epub 2020 Nov 10. PMID: 33172352; PMCID: PMC7659596.
- Manning BL, Harpole A, Harriott EM, Postolowicz K, Norton ES. Taking Language Samples Home: Feasibility, Reliability, and Validity of Child Language Samples Conducted Remotely With Video Chat Versus In-Person. J Speech Lang Hear Res. 2020 Dec 14;63(12):3982-3990. doi: 10.1044/2020_JSLHR-20-00202. Epub 2020 Nov 13. PMID: 33186507.
- Conde Blanco E, Manzanares I, Centeno M, Khawaja M, Betrán O, Donaire A, Carreño M. Epilepsy and lockdown: A survey of patients normally attending a Spanish centre. Acta Neurol Scand. 2021 Feb;143(2):206-209. doi: 10.1111/ane.13354. Epub 2020 Nov 17. PMID: 32990951; PMCID: PMC7646661.
- Prawiroharjo P, Pangeran D, Supriawan H, Lastri D, Mayza A, Zairinal RA, Dewi AR, Asmaniar F, Ramli Y. Increasing Traumatic Brain Injury Incidence during COVID-19 Pandemic in the Emergency Department of Cipto Mangunkusumo National General Hospital-A National Referral Hospital in Indonesia. Neurology. 2020 Nov 17;95(12 Suppl 2):S11. doi: 10.1212/01.wnl.0000719968.10580.81. PMID: 33199570.
- Smarrazzo A, Mariani R, Valentini F, Lombardi MH, Sinibaldi S, Peschiaroli E, Papa RE, Campana A. Three-fold increase in admissions for paediatric febrile convulsions during COVID-19 pandemic could indicate alternative virus symptoms. Acta Paediatr. 2021 Mar;110(3):939-940. doi: 10.1111/apa.15653. Epub 2020 Nov 18. PMID: 33145819.
- Lacritz LH, Carlew AR, Livingstone J, Bailey KC, Parker A, Diaz A. Patient Satisfaction with Telephone Neuropsychological Assessment. Arch Clin Neuropsychol. 2020 Nov 19;35(8):1240-1248. doi: 10.1093/arclin/acaa097. PMID: 33124648; PMCID: PMC7665292.
- Robles MC, Corches CL, Bradford M, Rice TS, Sukul D, Springer MV, Bailey S, Oliver A, Skolarus LE. Understanding and Informing Community Emergency Cardiovascular Disease Preparedness during the COVID-19 Pandemic: Stroke Ready. J Stroke Cerebrovasc Dis. 2021 Feb;30(2):105479. doi: 10.1016/j.jstrokecerebrovasdis.2020.105479. Epub 2020 Nov 19. PMID: 33246207; PMCID: PMC7674014.
- Mahawish KM, Watson I. Functional stroke-like presentations in the time of COVID-19. N Z Med J. 2020 Nov 20;133(1525):123-126. PMID: 33223556.
- Paolucci M, Biguzzi S, Cordici F, Lotti EM, Morresi S, Romoli M, Strumia S, Terlizzi R, Vidale S, Menarini M, Ruggiero M, Valentino A, Longoni M. Impact of COVID-19 pandemic on acute stroke care: facing an epidemiological paradox with a paradigm shift. Neurol Sci. 2021 Feb;42(2):399-406. doi: 10.1007/s10072-020-04914-4. Epub 2020 Nov 21. PMID: 33222101; PMCID: PMC7680213.
- Lima MC, Sander M, Dos Santos Lunardi M, Ribeiro LC, Rieger DK, Lin K, Moreira JD. Challenges in telemedicine for adult patients with drug-resistant epilepsy undergoing ketogenic diet treatment during the COVID-19 pandemic in the public healthcare system in Brazil. Epilepsy Behav. 2020 Dec;113:107529. doi: 10.1016/j.yebeh.2020.107529. Epub 2020 Nov 21. PMID: 33232896.
- Si Y, Sun L, Sun H, Niu Y, Mo Q. Epilepsy management during epidemic: A preliminary observation from western China. Epilepsy Behav. 2020 Dec;113:107528. doi: 10.1016/j.yebeh.2020.107528. Epub 2020 Nov 22. PMID: 33238237; PMCID: PMC7680647.
- Qureshi AI, Agunbiade S, Huang W, Akhtar IN, Abraham MG, Akhtar N, Al-Mufti F, Aytac E, Balgetir F, Grigoryan M, Gomez CR, Hassan AE, Jani V, Janjua NA, Jiao L, Khatri R, Kirmani JF, Kobayashi A, Kozak O, Lee J, Lobanova I, Mansour OY, Maud A, Mazighi M, Piotin M, Rodriguez GJ, Siddiq F, Suri MFK, Tekle WG. Changes in Neuroendovascular Procedural Volume During the COVID-19 Pandemic: An International Multicenter Study. J Neuroimaging. 2021 Jan;31(1):171-179. doi: 10.1111/jon.12803. Epub 2020 Nov 23. PMID: 33227167; PMCID: PMC7753603.
- Trivisano M, Specchio N, Pietrafusa N, Calabrese C, Ferretti A, Ricci R, Renzetti T, Raponi M, Vigevano F. Impact of COVID-19 pandemic on pediatric patients with epilepsy - The caregiver perspective. Epilepsy Behav. 2020 Dec;113:107527. doi: 10.1016/j.yebeh.2020.107527. Epub 2020 Nov 24. PMID: 33242768; PMCID: PMC7683298.
- Gul ZB, Atakli HD. Effect of the COVID-19 pandemic on drug compliance and stigmatization in patients with epilepsy. Epilepsy Behav. 2021 Jan;114(Pt A):107610. doi: 10.1016/j.yebeh.2020.107610. Epub 2020 Nov 24. PMID: 33243679; PMCID: PMC7685058.
- Postiglione E, Pizza F, Ingravallo F, Vignatelli L, Filardi M, Mangiaruga A, Antelmi E, Moresco M, Oriolo C, Pagotto U, Plazzi G. Impact of COVID-19 pandemic lockdown on narcolepsy type 1 management. Brain Behav. 2021 Jan;11(1):e01955. doi: 10.1002/brb3.1955. Epub 2020 Nov 28. PMID: 33247632; PMCID: PMC7744913.
- Pasca L, Zanaboni MP, Grumi S, Totaro M, Ballante E, Varesio C, De Giorgis V. Impact of COVID-19 pandemic in pediatric patients with epilepsy with neuropsychiatric comorbidities: A telemedicine evaluation. Epilepsy Behav. 2021 Feb;115:107519. doi: 10.1016/j.yebeh.2020.107519. Epub 2020 Nov 28. PMID: 33257293; PMCID: PMC7695947.
- Thorpe J, Ashby S, Hallab A, Ding D, Andraus M, Dugan P, Perucca P, Costello D, French JA, O'Brien TJ, Depondt C, Andrade DM, Sengupta R, Delanty N, Jette N, Newton CR, Brodie MJ, Devinsky O, Helen Cross J, Sander JW, Hanna J, Sen A; COVID-19 and Epilepsy (COV-E) Study Group. Evaluating risk to people with epilepsy during the COVID-19 pandemic: Preliminary findings from the COV-E study. Epilepsy Behav. 2021 Feb;115:107658. doi: 10.1016/j.yebeh.2020.107658. Epub 2020 Nov 28. PMID: 33341393; PMCID: PMC7698680.
- Puteikis K, Jasionis A, Mameniškienė R. Recalling the COVID-19 lockdown: Insights from patients with epilepsy. Epilepsy Behav. 2021 Feb;115:107573. doi: 10.1016/j.yebeh.2020.107573. Epub 2020 Nov 29. PMID: 33268021; PMCID: PMC7700727.
- Rosengard JL, Donato J, Ferastraoaru V, Zhao D, Molinero I, Boro A, Gursky J, Correa DJ, Galanopoulou AS, Hung C, Legatt AD, Patel P, Rubens E, Moshé SL, Haut S. Seizure control, stress, and access to care during the COVID-19 pandemic in New York City: The patient perspective. Epilepsia. 2021 Jan;62(1):41-50. doi: 10.1111/epi.16779. Epub 2020 Nov 30. PMID: 33258109; PMCID: PMC7753328.
- Grazzi L, Rizzoli P, Andrasik F. Effectiveness of mindfulness by smartphone, for patients with chronic migraine and medication overuse during the Covid-19 emergency. Neurol Sci. 2020 Dec;41(Suppl 2):461-462. doi: 10.1007/s10072-020-04659-0. PMID: 32794128; PMCID: PMC7426009.
- Sham L, Ciccone O, Patel AA. The COVID-19 pandemic and Community Health Workers: An opportunity to maintain delivery of care and education for families of children with epilepsy in Zambia. J Glob Health. 2020 Dec;10(2):020329. doi: 10.7189/jogh.10.020329. PMID: 33110529; PMCID: PMC7561215.
- Guo R, Shao X, Zhang C, Qian X. Sparse Adaptive Graph Convolutional Network for Leg Agility Assessment in Parkinson's Disease. IEEE Trans Neural Syst Rehabil Eng. 2020 Dec;28(12):2837-2848. doi: 10.1109/TNSRE.2020.3039297. Epub 2021 Jan 28. PMID: 33211661.
- Rath L, Bui MV, Ellis J, Carey J, Baker J, Taylor L, Fernando H, Taylor N, Savage P, Richards J, Zhong M, Kalincik T, Skibina O, Wesselingh R, Nguyen AL, Monif M, Butzkueven H, van der Walt A. Fast and safe: Optimising multiple sclerosis infusions during COVID-19 pandemic. Mult Scler Relat Disord. 2021 Jan;47:102642. doi: 10.1016/j.msard.2020.102642. Epub 2020 Dec 1. PMID: 33321356.
- Wang Y, Li B, Liu L. Telemedicine Experience in China: Our Response to the Pandemic and Current Challenges. Front Public Health. 2020 Dec 2;8:549669. doi: 10.3389/fpubh.2020.549669. PMID: 33425827; PMCID: PMC7793949.
- Kuitunen I, Ponkilainen VT, Launonen AP, Reito A, Hevonkorpi TP, Paloneva J, Mattila VM. The effect of national lockdown due to COVID-19 on emergency department visits. Scand J Trauma Resusc Emerg Med. 2020 Dec 4;28(1):114. doi: 10.1186/s13049-020-00810-0. PMID: 33276799; PMCID: PMC7716110.
- Wanigasinghe J, Jayawickrama A, Hewawitharana G, Munasinghe J, Weeraratne CT, Ratnayake P, Wijesekara DS, Fernando S, Rupasinghe P. Experience during COVID-19 lockdown and self-managing strategies among caregivers of children with epilepsy: A study from low middle income country. Seizure. 2021 Jan;84:112-115. doi: 10.1016/j.seizure.2020.12.001. Epub 2020 Dec 8. PMID: 33321430.
- Saliba-Gustafsson EA, Miller-Kuhlmann R, Kling SMR, Garvert DW, Brown-Johnson CG, Lestoquoy AS, Verano MR, Yang L, Falco-Walter J, Shaw JG, Asch SM, Gold CA, Winget M. Rapid Implementation of Video Visits in Neurology During COVID-19: Mixed Methods Evaluation. J Med Internet Res. 2020 Dec 9;22(12):e24328. doi: 10.2196/24328. PMID: 33245699; PMCID: PMC7732357.
- Bertran Recasens B, Povedano Panadés M, Rubio MA. Impact of the COVID-19 pandemic on a cohort of ALS patients in Catalonia. Neurologia. 2021 Mar;36(2):187-189. English, Spanish. doi: 10.1016/j.nrl.2020.12.001. Epub 2020 Dec 11. PMID: 33454120; PMCID: PMC7833722.
- Dozières-Puyravel B, Auvin S. Usefulness, limitations, and parental opinion about teleconsultation for rare pediatric epilepsies. Epilepsy Behav. 2021 Feb;115:107656. doi: 10.1016/j.yebeh.2020.107656. Epub 2020 Dec 13. PMID: 33317938.
- Stafstrom CE, Sun LR, Kossoff EH, Dabrowski AK, Singhi S, Kelley SA. Diagnosing and managing childhood absence epilepsy by telemedicine. Epilepsy Behav. 2021 Feb;115:107404. doi: 10.1016/j.yebeh.2020.107404. Epub 2020 Dec 13. PMID: 33323339.
- Moalong KMC, Espiritu AI, Fernandez MLL, Jamora RDG. Treatment gaps and challenges in epilepsy care in the Philippines. Epilepsy Behav. 2021 Feb;115:107491. doi: 10.1016/j.yebeh.2020.107491. Epub 2020 Dec 13. PMID: 33323340.
- Smith M, Nakamoto M, Crocker J, Tiffany Morden F, Liu K, Ma E, Chong A, Van N, Vajjala V, Carrazana E, Viereck J, Liow K. Early impact of the COVID-19 pandemic on outpatient migraine care in Hawaii: Results of a quality improvement survey. Headache. 2021 Jan;61(1):149-156. doi: 10.1111/head.14030. Epub 2020 Dec 14. PMID: 33316097.
- Chen Y, Xia F, Li Y, Li H, Ma L, Hu X, You C. Changes in Characteristics, Treatment and Outcome in Patients with Hemorrhagic Stroke During COVID-19. J Stroke Cerebrovasc Dis. 2021 Mar;30(3):105536. doi: 10.1016/j.jstrokecerebrovasdis.2020.105536. Epub 2020 Dec 15. PMID: 33338705.
- Luther E, Burks J, Eichberg DG, Basil G, Berry K, Lu V, Shah A, Kaur G, Ivan M, Komotar R. Neuro-oncology practice guidelines from a high-volume surgeon at the COVID-19 epicenter. J Clin Neurosci. 2021 Mar;85:1-5. doi: 10.1016/j.jocn.2020.12.012. Epub 2020 Dec 16. PMID: 33581778; PMCID: PMC7834482.
- Banks J, Corrigan D, Grogan R, El-Naggar H, White M, Doran E, Synnott C, Fitzsimons M, Delanty N, Doherty CP. LoVE in a time of CoVID: Clinician and patient experience using telemedicine for chronic epilepsy management. Epilepsy Behav. 2021 Feb;115:107675. doi: 10.1016/j.yebeh.2020.107675. Epub 2020 Dec 17. PMID: 33342712.
- Carai A, Locatelli F, Mastronuzzi A. Delayed referral of pediatric brain tumors during COVID-19 pandemic. Neuro Oncol. 2020 Dec 18;22(12):1884-1886. doi: 10.1093/neuonc/noaa159. PMID: 32623465; PMCID: PMC7454759.
- Philip P, Dupuy L, Morin CM, de Sevin E, Bioulac S, Taillard J, Serre F, Auriacombe M, Micoulaud-Franchi JA. Smartphone-Based Virtual Agents to Help Individuals With Sleep Concerns During COVID-19 Confinement: Feasibility Study. J Med Internet Res. 2020 Dec 18;22(12):e24268. doi: 10.2196/24268. PMID: 33264099; PMCID: PMC7752183.
- Alonso R, Carvajal R, Boaventura M, Galleguillos L. Experience of South American MS and/or NMOSD experts in practice during the COVID-19 pandemic: Focus on Telemedicine. Mult Scler Relat Disord. 2021 Feb;48:102702. doi: 10.1016/j.msard.2020.102702. Epub 2020 Dec 19. PMID: 33360914; PMCID: PMC7749658.
- Li Y, Emmett CD, Cobbaert M, Sanders DB, Juel VC, Hobson-Webb LD, Massey JM, Gable KL, Raja SM, Gonzalez NL, Guptill JT. Knowledge and perceptions of the COVID-19 pandemic among patients with myasthenia gravis. Muscle Nerve. 2021 Mar;63(3):357-364. doi: 10.1002/mus.27130. Epub 2020 Dec 20. PMID: 33280141.
- Schneider RB, Omberg L, Macklin EA, Daeschler M, Bataille L, Anthwal S, Myers TL, Baloga E, Duquette S, Snyder P, Amodeo K, Tarolli CG, Adams JL, Callahan KF, Gottesman J, Kopil CM, Lungu C, Ascherio A, Beck JC, Biglan K, Espay AJ, Tanner C, Oakes D, Shoulson I, Novak D, Kayson E, Ray Dorsey E, Mangravite L, Schwarzschild MA, Simuni T; Parkinson Study Group AT-HOME PD Investigators. Design of a virtual longitudinal observational study in Parkinson's disease (AT-HOME PD). Ann Clin Transl Neurol. 2021 Feb;8(2):308-320. doi: 10.1002/acn3.51236. Epub 2020 Dec 22. PMID: 33350601; PMCID: PMC7886038.
- Ma L, Chen Y, Chen XL. [Management protocol and outcome assessment of ruptured intracranial aneurysm emergency surgery during coronavirus disease 2019 pandemic]. Zhonghua Yi Xue Za Zhi. 2020 Dec 22;100(47):3768-3774. Chinese. doi: 10.3760/cma.j.cn112137-20200814-02385. PMID: 33379841.
- Kristoffersen ES, Sandset EC, Winsvold BS, Faiz KW, Storstein AM. Experiences of telemedicine in neurological out-patient clinics during the COVID-19 pandemic. Ann Clin Transl Neurol. 2021 Feb;8(2):440-447. doi: 10.1002/acn3.51293. Epub 2020 Dec 30. PMID: 33377609; PMCID: PMC7886029.
- Raith EP, Luoma AMV, Earl M, Dalal M, Fairley S, Fox F, Hunt K, Willett C, Reddy U. Repurposing a Neurocritical Care Unit for the Management of Severely Ill Patients With COVID-19: A Retrospective Evaluation. J Neurosurg Anesthesiol. 2021 Jan;33(1):77-81. doi: 10.1097/ANA.0000000000000727. PMID: 32815827.
- Johnson KG, Sullivan SS, Nti A, Rastegar V, Gurubhagavatula I. The impact of the COVID-19 pandemic on sleep medicine practices. J Clin Sleep Med. 2021 Jan 1;17(1):79-87. doi: 10.5664/jcsm.8830. PMID: 32964828; PMCID: PMC7849634.
- Chowdhury T, Rizk AA, Daniels AH, Al Azazi E, Sharma D, Venkatraghavan L. Management of Acute Ischemic Stroke in the Interventional Neuroradiology Suite During the COVID-19 Pandemic: A Global Survey. J Neurosurg Anesthesiol. 2021 Jan;33(1):44-50. doi: 10.1097/ANA.0000000000000734. PMID: 33048865.
- Rajan S, Bebawy J, Avitsian R, Lee CZ, Rath G, Luoma A, Bilotta F, Pierce JT, Kofke WA. The Impact of the Global SARS-CoV-2 (COVID-19) Pandemic on Neuroanesthesiology Fellowship Programs Worldwide and the Potential Future Role for ICPNT Accreditation. J Neurosurg Anesthesiol. 2021 Jan;33(1):82-86. doi: 10.1097/ANA.0000000000000738. PMID: 33075035.
- Tavanaei R, Yazdani KO, Akhlaghpasand M, Zali A, Oraee-Yazdani S. Changed pattern of hospital admission in stroke during COVID-19 pandemic period in Iran: a retrospective study. Neurol Sci. 2021 Feb;42(2):445-453. doi: 10.1007/s10072-020-05030-z. Epub 2021 Jan 4. PMID: 33394194; PMCID: PMC7780589.
- Bravata DM, Perkins AJ, Myers LJ, Arling G, Zhang Y, Zillich AJ, Reese L, Dysangco A, Agarwal R, Myers J, Austin C, Sexson A, Leonard SJ, Dev S, Keyhani S. Association of Intensive Care Unit Patient Load and Demand With Mortality Rates in US Department of Veterans Affairs Hospitals During the COVID-19 Pandemic. JAMA Netw Open. 2021 Jan 4;4(1):e2034266. doi: 10.1001/jamanetworkopen.2020.34266. PMID: 33464319; PMCID: PMC7816100.
- Aly A, Pettorini B. COVID-19 lockdown presented a chance to evaluate emergency referrals to paediatric neurosurgical unit: a prospective cohort study. Childs Nerv Syst. 2021 Mar;37(3):729-732. doi: 10.1007/s00381-020-04985-w. Epub 2021 Jan 6. PMID: 33404729; PMCID: PMC7787122.
- Luo W, Li J, Li Z, Luo X, Chen M, Cai C. Effects of the COVID-19 pandemic on reperfusion therapy for acute ischemic stroke patients in Huizhou City, China. Neurol Sci. 2021 Feb;42(2):467-473. doi: 10.1007/s10072-020-04938-w. Epub 2021 Jan 7. PMID: 33409830; PMCID: PMC7787931.
- Valerio Pascua F, Diaz O, Medina R, Contreras B, Mistroff J, Espinosa D, Sekhon A, Paz Handal D, Pineda E, Vargas Pineda M, Pineda H, Diaz M, Lewis AS, Hesse H, Castro Lainez MT, Stevens ML, Sierra-Hoffman M, Ontai SC, VanBuren V. A multi-mechanism approach reduces length of stay in the ICU for severe COVID-19 patients. PLoS One. 2021 Jan 7;16(1):e0245025. doi: 10.1371/journal.pone.0245025. PMID: 33411780; PMCID: PMC7790264.
- Reilly C, Muggeridge A, Cross JH. The perceived impact of COVID-19 and associated restrictions on young people with epilepsy in the UK: Young people and caregiver survey. Seizure. 2021 Feb;85:111-114. doi: 10.1016/j.seizure.2020.12.024. Epub 2021 Jan 8. PMID: 33453591; PMCID: PMC7791316.
- Kapsner LA, Kampf MO, Seuchter SA, Gruendner J, Gulden C, Mate S, Mang JM, Schüttler C, Deppenwiese N, Krause L, Zöller D, Balig J, Fuchs T, Fischer P, Haverkamp C, Holderried M, Mayer G, Stenzhorn H, Stolnicu A, Storck M, Storf H, Zohner J, Kohlbacher O, Strzelczyk A, Schüttler J, Acker T, Boeker M, Kaisers UX, Kestler HA, Prokosch HU. Reduced Rate of Inpatient Hospital Admissions in 18 German University Hospitals During the COVID-19 Lockdown. Front Public Health. 2021 Jan 13;8:594117. doi: 10.3389/fpubh.2020.594117. PMID: 33520914; PMCID: PMC7838458.
- Giebel C, Sutcliffe C, Darlington-Pollock F, Green MA, Akpan A, Dickinson J, Watson J, Gabbay M. Health Inequities in the Care Pathways for People Living with Young- and Late-Onset Dementia: From Pre-COVID-19 to Early Pandemic. Int J Environ Res Public Health. 2021 Jan 14;18(2):686. doi: 10.3390/ijerph18020686. PMID: 33466948; PMCID: PMC7831042.
- Meyding-Lamadé U, Bassa B, Tibitanzl P, Davtyan A, Lamadé EK, Craemer EM. Telerehabilitation: von der virtuellen Welt zur Realität – Medizin im 21. Jahrhundert : Videogestützte Therapie in Zeiten von COVID-19 [Telerehabilitation: from the virtual world to reality-Medicine in the twenty-first century : Video-assisted treatment in times of COVID-19]. Nervenarzt. 2021 Feb;92(2):127-136. German. doi: 10.1007/s00115-020-01058-w. Epub 2021 Jan 18. PMID: 33459797; PMCID: PMC7812715.
- Wong A, Bhyat R, Srivastava S, Boissé Lomax L, Appireddy R. Patient Care During the COVID-19 Pandemic: Use of Virtual Care. J Med Internet Res. 2021 Jan 21;23(1):e20621. doi: 10.2196/20621. PMID: 33326410; PMCID: PMC7822645.
- Raucci U, Musolino AM, Di Lallo D, Piga S, Barbieri MA, Pisani M, Rossi FP, Reale A, Ciofi Degli Atti ML, Villani A, Raponi M. Impact of the COVID-19 pandemic on the Emergency Department of a tertiary children's hospital. Ital J Pediatr. 2021 Jan 29;47(1):21. doi: 10.1186/s13052-021-00976-y. PMID: 33514391; PMCID: PMC7844808.
- Bhambhvani HP, Rodrigues AJ, Yu JS, Carr JB 2nd, Hayden Gephart M. Hospital Volumes of 5 Medical Emergencies in the COVID-19 Pandemic in 2 US Medical Centers. JAMA Intern Med. 2021 Feb 1;181(2):272-274. doi: 10.1001/jamainternmed.2020.3982. PMID: 33104161; PMCID: PMC7589046.
- Gupta L, Lilleker JB, Agarwal V, Chinoy H, Aggarwal R. COVID-19 and myositis - unique challenges for patients. Rheumatology (Oxford). 2021 Feb 1;60(2):907-910. doi: 10.1093/rheumatology/keaa610. PMID: 33175137; PMCID: PMC7717379.
- Kristoffersen ES, Winsvold BS, Sandset EC, Storstein AM, Faiz KW. Experiences, distress and burden among neurologists in Norway during the COVID-19 pandemic. PLoS One. 2021 Feb 4;16(2):e0246567. doi: 10.1371/journal.pone.0246567. PMID: 33539418; PMCID: PMC7861439.
- Di Lorito C, Duff C, Rogers C, Tuxworth J, Bell J, Fothergill R, Wilkinson L, Bosco A, Howe L, O'Brien R, Godfrey M, Dunlop M, van der Wardt V, Booth V, Logan P, Cowley A, Harwood RH. Tele-Rehabilitation for People with Dementia during the COVID-19 Pandemic: A Case-Study from England. Int J Environ Res Public Health. 2021 Feb 10;18(4):1717. doi: 10.3390/ijerph18041717. PMID: 33578949.
- Mercuri E, Zampino G, Morsella A, Pane M, Onesimo R, Angioletti C, Valentini P, Rendeli C, Ruggiero A, Nanni L, Chiaretti A, Vento G, Korn D, Meneschincheri E, Sergi P, Scambia G, Ricciardi W, Cambieri A, De Belvis AG. Contactless: a new personalised telehealth model in chronic pediatric diseases and disability during the COVID-19 era. Ital J Pediatr. 2021 Feb 12;47(1):29. doi: 10.1186/s13052-021-00975-z. PMID: 33579344; PMCID: PMC7880513.
- Nora M, Giannarelli M, Zicchinella C, Mammi P, Ranza E, Brianti R. An Experience of Multiple Sclerosis Telerehabilitation During the COVID-19 Pandemic. Am J Phys Med Rehabil. 2021 Mar 1;100(3):214. doi: 10.1097/PHM.0000000000001679. PMID: 33433113.
- Rosales JS, Rodriguez-Perez MS, Ameriso SF. Efecto de la pandemia COVID-19 y la cuarentena en el número de consultas, subtipos y tratamiento del accidente cerebrovascular en un centro neurológico de Argentina [Effect of the COVID-19 pandemic and preventive social isolation measures on the number of outpatient visits, hospitalizations and treatment of cerebrovascular accident in a neurological center in Argentina]. Medicina (B Aires). 2020;80 Suppl 6:65-70. Spanish. PMID: 33481735.
- Romero-Imbroda J, Reyes-Garrido V, Ciano-Petersen NL, Serrano-Castro PJ. Emergency implantation of a teleneurology service at the neuromuscular unit of Hospital Regional de Málaga during the SARS-CoV-2 pandemic. Neurologia. 2020 Jul-Aug;35(6):415-417. English, Spanish. doi: 10.1016/j.nrl.2020.05.008. Epub 2020 May 23. PMID: 32571553; PMCID: PMC7245281.
- Bobo E, Lin L, Acquaviva E, Caci H, Franc N, Gamon L, Picot MC, Pupier F, Speranza M, Falissard B, Purper-Ouakil D. Comment les enfants et adolescents avec le trouble déficit d’attention/hyperactivité (TDAH) vivent-ils le confinement durant la pandémie COVID-19 ? [How do children and adolescents with Attention Deficit Hyperactivity Disorder (ADHD) experience lockdown during the COVID-19 outbreak?]. Encephale. 2020 Jun;46(3S):S85-S92. French. doi: 10.1016/j.encep.2020.05.011. Epub 2020 Jun 7. PMID: 32522407; PMCID: PMC7276130.
- Levinson AJ, Bousfield J, Douglas W, Ayers S, Sztramko R. A Novel Educational Prescription Web-Based Application to Support Education for Caregivers of People Living With Dementia: Development and Usability Study With Clinicians. JMIR Hum Factors. 2020 Dec 4;7(4):e23904. doi: 10.2196/23904. PMID: 33275103; PMCID: PMC7748956.
- Giebel C, Lord K, Cooper C, Shenton J, Cannon J, Pulford D, Shaw L, Gaughan A, Tetlow H, Butchard S, Limbert S, Callaghan S, Whittington R, Rogers C, Komuravelli A, Rajagopal M, Eley R, Watkins C, Downs M, Reilly S, Ward K, Corcoran R, Bennett K, Gabbay M. A UK survey of COVID-19 related social support closures and their effects on older people, people with dementia, and carers. Int J Geriatr Psychiatry. 2021 Mar;36(3):393-402. doi: 10.1002/gps.5434. Epub 2020 Sep 25. PMID: 32946619; PMCID: PMC7536967.
- Gedde MH, Husebo BS, Erdal A, Puaschitz NG, Vislapuu M, Angeles RC, Berge LI. Access to and interest in assistive technology for home-dwelling people with dementia during the COVID-19 pandemic (PAN.DEM). Int Rev Psychiatry. 2021 Jan 8:1-8. doi: 10.1080/09540261.2020.1845620. Epub ahead of print. PMID: 33416012.
- Hoyer C, Ebert A, Huttner HB, Puetz V, Kallmünzer B, Barlinn K, Haverkamp C, Harloff A, Brich J, Platten M, Szabo K. Acute Stroke in Times of the COVID-19 Pandemic: A Multicenter Study. Stroke. 2020 Jul;51(7):2224-2227. doi: 10.1161/STROKEAHA.120.030395. Epub 2020 Jun 9. PMID: 32516064.
- Hautz WE, Sauter TC, Exadakytlos AK, Krummrey G, Schauber S, Müller M. Barriers to seeking emergency care during the COVID-19 pandemic may lead to higher morbidity and mortality - a retrospective study from a Swiss university hospital. Swiss Med Wkly. 2020 Aug 11;150:w20331. doi: 10.4414/smw.2020.20331. PMID: 32799308.
- Gali K, Joshi S, Hueneke S, Katzenbach A, Radecki L, Calabrese T, Fletcher L, Trandafir C, Wilson C, Goyal M, Wusthoff CJ, Le Pichon JB, Corvalan R, Golson A, Hardy J, Smith M, Cook E, Bonkowsky JL. Barriers, access and management of paediatric epilepsy with telehealth. J Telemed Telecare. 2020 Nov 12:1357633X20969531. doi: 10.1177/1357633X20969531. Epub ahead of print. PMID: 33183129.
- Caze T, Dorsman KA, Carlew AR, Diaz A, Bailey KC. Can You Hear Me Now? Telephone-Based Teleneuropsychology Improves Utilization Rates in Underserved Populations. Arch Clin Neuropsychol. 2020 Nov 19;35(8):1234-1239. doi: 10.1093/arclin/acaa098. PMID: 33210720.
- Jeste S, Hyde C, Distefano C, Halladay A, Ray S, Porath M, Wilson RB, Thurm A. Changes in access to educational and healthcare services for individuals with intellectual and developmental disabilities during COVID-19 restrictions. J Intellect Disabil Res. 2020 Sep 17. doi: 10.1111/jir.12776. Epub ahead of print. PMID: 32939917.
- Giebel C, Pulford D, Cooper C, Lord K, Shenton J, Cannon J, Shaw L, Tetlow H, Limbert S, Callaghan S, Whittington R, Rogers C, Komuravelli A, Rajagopal M, Eley R, Downs M, Reilly S, Ward K, Gaughan A, Butchard S, Beresford J, Watkins C, Bennett K, Gabbay M. COVID-19-related social support service closures and mental well-being in older adults and those affected by dementia: a UK longitudinal survey. BMJ Open. 2021 Jan 17;11(1):e045889. doi: 10.1136/bmjopen-2020-045889. PMID: 33455941; PMCID: PMC7813330.
- Daniel M Fountain, Rory J Piper, Michael T C Poon, Georgios Solomou, Paul M Brennan, Yasir A Chowdhury, Francesca Colombo, Tarek Elmoslemany, Frederick G Ewbank, Paul L Grundy, Md Tanvir Hasan, Molly Hilling, Peter J Hutchinson, Konstantina Karabatsou, Angelos G Kolias, Nathan J McSorley, Christopher P Millward, Isaac Phang, Puneet Plaha, Stephen J Price, Ola Rominiyi, William Sage, Syed Shumon, Ines L Silva, Stuart J Smith, Surash Surash, Simon Thomson, Jun Yi Lau, Colin Watts, Michael D Jenkinson, on behalf of the British Neurosurgical Trainee Research Collaborative (BNTRC), CovidNeuroOnc: a UK multi-centre, prospective cohort study of the impact of the COVID-19 pandemic on the neuro-oncology service, Neuro-Oncology Advances, 2021;, vdab014, <https://doi.org/10.1093/noajnl/vdab014>
- Santamato A, Facciorusso S, Spina S, Cinone N, Avvantaggiato C, Santoro L, Ciritella C, Smania N, Picelli A, Gasperini G, Molteni F, Baricich A, Fiore P. Discontinuation of botulinum neurotoxin type-A treatment during COVID-19 pandemic: an Italian survey in post stroke and traumatic brain injury patients living with spasticity. Eur J Phys Rehabil Med. 2020 Dec 2. doi: 10.23736/S1973-9087.20.06478-3. Epub ahead of print. PMID: 33263248.
- Spalletta G, Porcari DE, Banaj N, Ciullo V, Palmer K. Effects of COVID-19 Infection Control Measures on Appointment Cancelation in an Italian Outpatient Memory Clinic. Front Psychiatry. 2020 Nov 30;11:599844. doi: 10.3389/fpsyt.2020.599844. PMID: 33329152; PMCID: PMC7733990.
- Barguilla A, Fernández-Lebrero A, Estragués-Gázquez I, García-Escobar G, Navalpotro-Gómez I, Manero RM, Puente-Periz V, Roquer J, Puig-Pijoan A. Effects of COVID-19 Pandemic Confinement in Patients With Cognitive Impairment. Front Neurol. 2020 Nov 24;11:589901. doi: 10.3389/fneur.2020.589901. PMID: 33329337; PMCID: PMC7732426.
- Miller WR, Von Gaudecker J, Tanner A, Buelow JM. Epilepsy self-management during a pandemic: Experiences of people with epilepsy. Epilepsy Behav. 2020 Oct;111:107238. doi: 10.1016/j.yebeh.2020.107238. Epub 2020 Jun 25. PMID: 32593874; PMCID: PMC7316066.
- Sharma R, Kuohn LR, Weinberger DM, Warren JL, Sansing LH, Jasne A, Falcone G, Dhand A, Sheth KN. Excess Cerebrovascular Mortality in the United States During the COVID-19 Pandemic. Stroke. 2021 Jan;52(2):563-572. doi: 10.1161/STROKEAHA.120.031975. Epub 2021 Jan 12. PMID: 33430638; PMCID: PMC7834664.
- Abejón D, Monzón EM, Deer T, Hagedorn JM, Araujo R, Abad C, Rios A, Zamora A, Vallejo R. How to Restart the Interventional Activity in the COVID-19 Era: The Experience of a Private Pain Unit in Spain. Pain Pract. 2020 Nov;20(8):820-828. doi: 10.1111/papr.12951. Epub 2020 Oct 9. PMID: 32969188; PMCID: PMC7536921.
- Bova SM, Basso M, Bianchi MF, Savaré L, Ferrara G, Mura E, Redaelli MG, Olivieri I, Veggiotti P; Milan COVID-19 and Child Neurology Study Group. Impact of COVID-19 lockdown in children with neurological disorders in Italy. Disabil Health J. 2020 Dec 16:101053. doi: 10.1016/j.dhjo.2020.101053. Epub ahead of print. PMID: 33358228; PMCID: PMC7832678.
- Millevert C, Van Hees S, Siewe Fodjo JN, Wijtvliet V, Faria de Moura Villela E, Rosso B, Gil-Nagel A, Weckhuysen S, Colebunders R. Impact of COVID-19 on the lives and psychosocial well-being of persons with epilepsy during the third trimester of the pandemic: Results from an international, online survey. Epilepsy Behav. 2021 Jan 29;116:107800. doi: 10.1016/j.yebeh.2021.107800. Epub ahead of print. PMID: 33571838.
- Rathore C, Baheti N, Bansal AR, Jabeen SA, Gopinath S, Jagtap S, Patil S, Suryaprabha T, Jayalakshmi S, Ravat S, Nayak DS, Prakash S, Rana K, Jaiswal SK, Khan FR, Murthy JM, Radhakrishnan K. Impact of COVID-19 pandemic on epilepsy practice in India: A tripartite survey. Seizure. 2021 Jan 8;86:60-67. doi: 10.1016/j.seizure.2020.12.025. Epub ahead of print. PMID: 33550135; PMCID: PMC7837209.
- Kwan J, Brown M, Bentley P, Brown Z, D'Anna L, Hall C, Halse O, Jamil S, Jenkins H, Kalladka D, Patel M, Rane N, Singh A, Taylor E, Venter M; Lobotesis, Banerjee S. Impact of COVID-19 Pandemic on a Regional Stroke Thrombectomy Service in the United Kingdom. Cerebrovasc Dis. 2020 Dec 11:1-7. doi: 10.1159/000512603. Epub ahead of print. PMID: 33311017; PMCID: PMC7801959.
- Giebel C, Cannon J, Hanna K, Butchard S, Eley R, Gaughan A, Komuravelli A, Shenton J, Callaghan S, Tetlow H, Limbert S, Whittington R, Rogers C, Rajagopal M, Ward K, Shaw L, Corcoran R, Bennett K, Gabbay M. Impact of COVID-19 related social support service closures on people with dementia and unpaid carers: a qualitative study. Aging Ment Health. 2020 Sep 21:1-8. doi: 10.1080/13607863.2020.1822292. Epub ahead of print. PMID: 32954794.
- Zhao J, Li H, Kung D, Fisher M, Shen Y, Liu R. Impact of the COVID-19 Epidemic on Stroke Care and Potential Solutions. Stroke. 2020 Jul;51(7):1996-2001. doi: 10.1161/STROKEAHA.120.030225. Epub 2020 May 20. PMID: 32432997; PMCID: PMC7258753.
- Manacorda T, Bandiera P, Terzuoli F, Ponzio M, Brichetto G, Zaratin P, Bezzini D, Battaglia MA. Impact of the COVID-19 pandemic on persons with multiple sclerosis: Early findings from a survey on disruptions in care and self-reported outcomes. J Health Serv Res Policy. 2020 Dec 18:1355819620975069. doi: 10.1177/1355819620975069. Epub ahead of print. PMID: 33337256.
- Vollmuth C, Miljukov O, Abu-Mugheisib M, Angermaier A, Barlinn J, Busetto L, Grau AJ, Guenther A, Gumbinger C, Hubert N, Hüttemann K, Klingner C, Naumann M, Palm F, Remi J, Rücker V, Schessl J, Schlachetzki F, Schuppner R, Schwab S, Schwartz A, Trommer A, Urbanek C, Volbers B, Weber J, Wojciechowski C, Worthmann H, Zickler P, Heuschmann PU, Haeusler KG, Hubert GJ. Impact of the COVID-19 pandemic on stroke teleconsultations in Germany in the first half of 2020. Eur J Neurol. 2021 Feb 22. doi: 10.1111/ene.14787. Epub ahead of print. PMID: 33619788.
- Masi A, Mendoza Diaz A, Tully L, Azim SI, Woolfenden S, Efron D, Eapen V. Impact of the COVID-19 pandemic on the well-being of children with neurodevelopmental disabilities and their parents. J Paediatr Child Health. 2021 Jan 10. doi: 10.1111/jpc.15285. Epub ahead of print. PMID: 33426739.
- Marra DE, Hoelzle JB, Davis JJ, Schwartz ES. Initial changes in neuropsychologists clinical practice during the COVID-19 pandemic: A survey study. Clin Neuropsychol. 2020 Oct-Nov;34(7-8):1251-1266. doi: 10.1080/13854046.2020.1800098. Epub 2020 Jul 29. PMID: 32723158.
- Nagaratnam K, Harston G, Flossmann E, Canavan C, Geraldes RC, Edwards C. Innovative use of artificial intelligence and digital communication in acute stroke pathway in response to COVID-19. Future Healthc J. 2020 Jun;7(2):169-173. doi: 10.7861/fhj.2020-0034. PMID: 32550287; PMCID: PMC7296572.
- Delussi M, Gentile E, Coppola G, Prudenzano AMP, Rainero I, Sances G, Abagnale C, Caponnetto V, De Cesaris F, Frattale I, Guaschino E, Marcinnò A, Ornello R, Pistoia F, Putortì A, Roca ME, Roveta F, Lupi C, Trojano M, Pierelli F, Geppetti P, Sacco S, de Tommaso M. Investigating the Effects of COVID-19 Quarantine in Migraine: An Observational Cross-Sectional Study From the Italian National Headache Registry (RICe). Front Neurol. 2020 Nov 10;11:597881. doi: 10.3389/fneur.2020.597881. PMID: 33240213; PMCID: PMC7683429.
- Aguiar de Sousa D, van der Worp HB, Caso V, Cordonnier C, Strbian D, Ntaios G, Schellinger PD, Sandset EC; European Stroke Organisation. Maintaining stroke care in Europe during the COVID-19 pandemic: Results from an international survey of stroke professionals and practice recommendations from the European Stroke Organisation. Eur Stroke J. 2020 Sep;5(3):230-236. doi: 10.1177/2396987320933746. Epub 2020 Jun 10. PMID: 33072876; PMCID: PMC7538757.
- McConachie D, McConachie N, White P, Crossley R, Izzath W. Mechanical thrombectomy for acute ischaemic stroke during the COVID-19 pandemic: changes to UK practice and lessons learned. Clin Radiol. 2020 Oct;75(10):795.e7-795.e13. doi: 10.1016/j.crad.2020.07.001. Epub 2020 Jul 10. PMID: 32682524; PMCID: PMC7351427.
- Suarez-Cedeno G, Pantelyat A, Mils K, Murthy M, Alshaikh J, Rosenthal L, Bang J, Moukheiber E. Movement Disorders Virtual Fellowship Training in Times of Coronavirus Disease 2019: A Single-Center Experience. Telemed J E Health. 2021 Jan 27. doi: 10.1089/tmj.2020.0419. Epub ahead of print. PMID: 33512273.
- Rai AT, Leslie-Mazwi TM, Fargen KM, Pandey AS, Dabus G, Hassan AE, Fraser JF, Hirsch JA, Gupta R, Hanel R, Yoo AJ, Bozorgchami H, Fiorella D, Mocco J, Arthur AS, Zaidat O, Siddiqui AH. Neuroendovascular clinical trials disruptions due to COVID-19. Potential future challenges and opportunities. J Neurointerv Surg. 2020 Sep;12(9):831-835. doi: 10.1136/neurintsurg-2020-016502. Epub 2020 Jun 30. PMID: 32606103; PMCID: PMC7371488.
- Courtney E, Blackburn D, Reuber M. Neurologists' perceptions of utilising tele-neurology to practice remotely during the COVID-19 pandemic. Patient Educ Couns. 2021 Jan 2:S0738-3991(20)30691-1. doi: 10.1016/j.pec.2020.12.027. Epub ahead of print. PMID: 33478853.
- Cuffaro L, Carvalho V, Di Liberto G, Klinglehoefer L, Sauerbier A, Garcia-Azorin D, Tábuas-Pereira M, Vashchenko N, Moro E, Bassetti CLA. Neurology training and research in the COVID-19 pandemic: a survey of the Resident and Research Fellow Section of the European Academy of Neurology. Eur J Neurol. 2020 Dec 23. doi: 10.1111/ene.14696. Epub ahead of print. PMID: 33368826.
- Cavallieri F, Sireci F, Fioravanti V, Toschi G, Rispoli V, Antonelli F, Costantini M, Ghirotto L, Valzania F. Parkinson's disease patients' needs during the COVID-19 pandemic in a red zone: A framework analysis of open-ended survey questions. Eur J Neurol. 2021 Jan 18. doi: 10.1111/ene.14745. Epub ahead of print. PMID: 33460507.
- Banerjee D, Vajawat B, Varshney P, Rao TS. Perceptions, Experiences, and Challenges of Physicians Involved in Dementia Care During the COVID-19 Lockdown in India: A Qualitative Study. Front Psychiatry. 2021 Jan 20;11:615758. doi: 10.3389/fpsyt.2020.615758. PMID: 33551877; PMCID: PMC7854902.
- Khosravani H, Rajendram P, Notario L, Chapman MG, Menon BK. Protected Code Stroke: Hyperacute Stroke Management During the Coronavirus Disease 2019 (COVID-19) Pandemic. Stroke. 2020 Jun;51(6):1891-1895. doi: 10.1161/STROKEAHA.120.029838. Epub 2020 Apr 1. PMID: 32233980; PMCID: PMC7258750.
- Yadala S, Nalleballe K, Sharma R, Lotia M, Kapoor N, Veerapaneni KD, Kovvuru S, Onteddu S. Resident Education During COVID-19 Pandemic: Effectiveness of Virtual Electroencephalogram Learning. Cureus. 2020 Oct 22;12(10):e11094. doi: 10.7759/cureus.11094. PMID: 33110712; PMCID: PMC7581218.
- Malec JF, Salisbury DB, Anders D, Dennis L, Groff AR, Johnson M, Murphy MP, Smith GT. Response to the COVID-19 Pandemic Among Posthospital Brain Injury Rehabilitation Providers. Arch Phys Med Rehabil. 2021 Mar;102(3):549-555. doi: 10.1016/j.apmr.2020.10.137. Epub 2020 Nov 27. PMID: 33253694; PMCID: PMC7695439.
- Kucap M, Nadolny K, Ładny JR, Zyśko D, Gałązkowski R, Gąsior M, Kraska W. RETROSPECTIVE ANALYSIS OF INTERVENTIONS PERFORMED BY EMERGENCY MEDICAL TEAMS IN POLAND BEFORE AND DURING THE SARS COV-2 PANDEMIC. Wiad Lek. 2020;73(8):1659-1662. PMID: 33055329.
- Alonso de Leciñana M, Castellanos M, Ayo-Martín Ó, Morales A; Stroke Group - Spanish Society of Neurology. Stroke care during the COVID-19 outbreak in Spain: the experience of Spanish stroke units. Stroke Vasc Neurol. 2020 Dec 4:svn-2020-000678. doi: 10.1136/svn-2020-000678. Epub ahead of print. PMID: 33277364; PMCID: PMC7722359.
- Taimkao S, Tiamkao S. Tele-neurology during the COVID-19 pandemic as a solution for bridging the healthcare Gap. J Med Assoc Thai 2021;104(2):94-96.
- Fonkem E, Gatson NTN, Tadipatri R, Cole S, Azadi A, Sanchez M, Stefanowicz E, Notes A.Telemedicine review in neuro-oncology: comparative experiential analysis for Barrow Neurological Institute and Geisinger Health during the 2020 COVID-19 pandemic. Neuro-Oncology Practice 2020.
- Rochette AD, Rahman-Filipiak A, Spencer RJ, Marshall D, Stelmokas JE. Teleneuropsychology practice survey during COVID-19 within the United States. Appl Neuropsychol Adult. 2021 Jan 20:1-11. doi: 10.1080/23279095.2021.1872576. Epub ahead of print. PMID: 33471555.
- Mostacci B, Licchetta L, Cacciavillani C, Di Vito L, Ferri L, Menghi V, Stipa C, Avoni P, Provini F, Muccioli L, Vignatelli L, Mazzoni S, Tinuper P, Bisulli F. The Impact of the COVID-19 Pandemic on People With Epilepsy. An Italian Survey and a Global Perspective. Front Neurol. 2020 Dec 18;11:613719. doi: 10.3389/fneur.2020.613719. PMID: 33391172; PMCID: PMC7775598.
